# Tracing the structural evolution of eukaryotic ATP binding cassette transporter superfamily

**DOI:** 10.1038/srep16724

**Published:** 2015-11-18

**Authors:** Jie Xiong, Jinmei Feng, Dongxia Yuan, Jun Zhou, Wei Miao

**Affiliations:** 1 Key Laboratory of Aquatic Biodiversity and Conservation, Institute of Hydrobiology, Chinese Academy of Sciences, Wuhan, 430072, PR China; 2 Department of Organismic and Evolutionary Biology, Harvard University, MA 02138, USA

## Abstract

The ATP binding cassette (ABC) transporters superfamily is one of the largest classes of membrane proteins. The core of the ABC transporter protein is composed of transmembrane domains (TMDs) and nucleotide binding domains (NBD). Eukaryotes ABC transporters are classified into seven main families (ABCA to ABCG) based on sequence similarity and domain organizations. With different domain number and domain organizations, eukaryote ABC transporters show diverse structures: the single structure (NBD or TMD), the ABC2 structure (NBD-NBD), the half structure (TMD-NBD or NBD-TMD) and the full structure (TMD-NBD-TMD-NBD or NBD-TMD-NBD-TMD). However, studies on how various ABC transporter gene structures evolved is still absent. Therefore, in this study, we comprehensively investigated the structural evolution of eukaryotic ABC transporters. The seven eukaryote ABC transporter families (A to G) fell into three groups: A&G group, B,C&D group and E&F group. There were at least four times the number of NBD and TMD fusion events in the origin of the half structure transporter. Two fusion modes were found in the full and ABC2 structure origination. Based on these findings, we present a putative structural evolutionary path of eukaryote ABC transporters that will increase our understanding on their origin, divergence and function.

The movement of molecules into and out of cells is mediated by cell membranes, in many cases by specialized membrane proteins known as transporters. Integral membrane ATP Binding Cassette (ABC) transporters are ubiquitous in all three domains of life and form one of the largest classes of membrane proteins^1^,^2^. Through the hydrolysis of ATP to generate energy, ABC transporters move a wide variety of substrates across membranes, including ions, sugars, amino acids, polypeptides, toxic metabolites, xenobiotics, and even drugs and toxins. A typical structure of eukaryotic ABC transporters consists of two conserved domains: a transmembrane domain (TMD) and a nucleotide binding domain (NBD)^3^. TMDs typically contain several transmembrane helices, and NBDs have several conserved motifs: Walker A motif, Q loop, Signature motif and Walker B motif^1^,^4^. According to domain organizations and primary sequence homology, eukaryotic ABC transporters have been classified into seven families, from ABCA to ABCG^5^,^6^,^7^,^8^,^9^,^10^,^11^,^12^. Members of the seven ABC transporter families are widely distributed in eukaryotes. In the ABC transporter superfamily, the sizes of the genes vary drastically across species, and can possess more than a hundred copies in some organisms such as the brassicacean *Arabidopsis thaliana*^13^ and the ciliate *Tetrahymena thermophila*^14^. Despite being called transporters, two eukaryotic ABC transporter families (ABCE and ABCF) do not function as transporters but are involved in other cellular processes including ribonuclease inhibition and translational control^15^,^16^, mainly because they no longer carry TMD domains. The structures of ABC transporters are diverse owing to their different domain compositions. A transporter protein with NBD-TMD-NBD-TMD or TMD-NBD-TMD-NBD organization is designated as a full transporter (or full structure)^17^; A half transporter (or half structure) is composed of only one TMD and one NBD, with either NBD-TMD or TMD-NBD organization. Some transporters encoded with only a single TMD or NBD are defined as single domain structure. In non-transporter ABC proteins, only NBDs are present at both the N- and the C-terminus. Such cases with NBD-NBD organization is referred to as the ABC2 structure.

Most previous work on ABC transporter genes focused on either function^18^ or phylogeny^7^,^10^,^11^,^13^,^14^,^19^. Two large-scale analyses of ABC transporters were conducted across multiple kingdoms. One study investigated ABC transporters in 20 model organisms with the objective of identifying the function of proteins based on sequence similarity using a simple method known as the “function transfer rule”^20^. The other study described two large novel clusters of bacterial multidrug transporters related to the eukaryotic ABCB and ABCC families, and proposed an evolutionary hypothesis that NBDs of many transporter families derive from the common ancestor of prokaryotes and eukaryotes^21^. Nevertheless, a comprehensive analysis of how various ABC transporter gene structures evolved is still absent.

The rapidly accumulating genomic data of diverse species have provided an excellent opportunity to infer the structural evolution of this ancient gene superfamily. Here, we investigated the phylogeny of the ABC transporter superfamily using proteins from diverse eukaryote kingdoms, analyzed domain features, and proposed a detailed path that traces the evolution of the diverse structures of the ABC transporter superfamily.

## Results

### Distribution of ABC transporters

In the present study, ABC transporters were identified in 79 evolutionarily diverse eukaryotic genomes (Table S1) that are representatives of eight eukaryotes lineages - alveolata, amoebozoa, euglenozoa, fungi, metazoa, fornicata, choanoflagellida and viridiplantae, and 2302 prokaryote genomes (Table S2) including those of both bacteria and archaea. The ABC transporter is a large superfamily with many families. Proteins in different families may have low sequence similarities thus increasing the difficulty to identify these proteins. To avoid this, members in all known eukaryotic ABC transporter families were used as seed proteins and searched against all the selected genomes by BLASTP using a very relaxed E-value cutoff (10). All the BLASTP hits (ABC transporter candidates) were then scanned using more sensitive profile-based domain searching. Proteins with an NBD domain were regarded as ABC transporters. Domain compositions and gene structures were also investigated.

**Figure 1:**
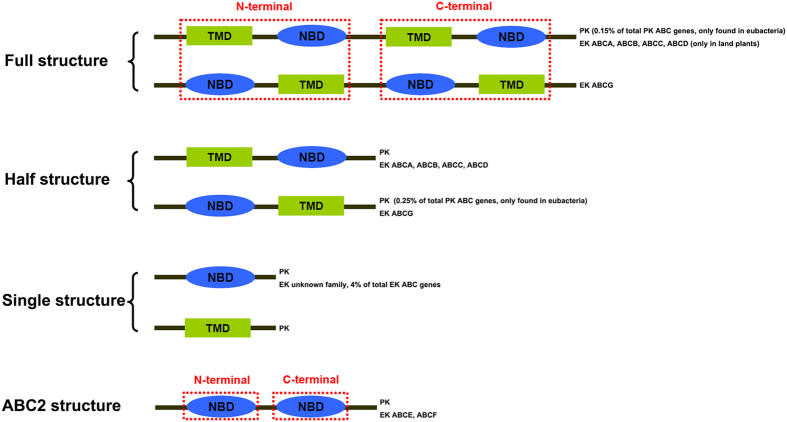
Illustration of the different structures in the ABC transporter superfamily. Full structure represents the ABC transporter gene that encodes two TMDs and two NBDs; Half structure represents the ABC transporter gene that encodes one TMD and one NBD; Single structure represents the ABC transporter gene with a single TMD or NBD; ABC2 structure represents the ABC transporter gene with only two NBDs. The families possessing certain structures are listed on the right.

We found that, in prokaryotes, the ABC genes mainly encode TMD or NBD single structure, followed by TMD-NBD half structure and ABC2 NBD-NBD structure. We also discovered NBD-TMD half structure and TMD-NBD-TMD-NBD full structure (Fig. 1). Our findings are consistent with previous studies that prokaryotic ABC proteins predominantly contain single and half structures. By contrast, eukaryotes mainly contain half and full structures (Fig. 1). It is therefore likely that ABC transporters have undergone an evolutionary path from simple to complex structures (i.e. from single to half or ABC2 structure, then from half to full structure).

All seven eukaryote ABC transporter families are present in eukaryotic species (Table S1) suggesting they may have originated before the last eukaryotic common ancestor (LECA). TMD-NBD half structure was found in ABCA, ABCB and ABCD families, and TMD-NBD-TMD-NBD full structure was found in ABCA, ABCB and ABCC families. However, both NBD-TMD half structure and NBD-TMD-NBD-TMD full structure were only found in the ABCG family. NBD-NBD ABC2 structure was only detected in ABCE and ABCF families (Fig. 1 and Table S1). Finally, there were sporadic genes showing half or single structures in the ABCC family, single structures in ABCE, ABCF and unknown families (no high-score blast hit found in ABCA-ABCG family), and full structure in the ABCD family (Fig. 1 and Table S1). Additional manual verification of these sporadically detected genes suggests that these structures were more likely derived from either incomplete genome assembly or gene mis-prediction (Table S1), except for the ABCD full structure transporters. ABCD full structure transporters were only found in three land plants (*Physcomitrella patens*, *Arabidopsis thaliana* and *Oryza sativa* Japonica Group) among the 79 eukaryotes (Table S3 and Fig. 1). We further checked the ABCD genes in the NCBI plant Refseq database, a pool of viridiplantae (green algae + land plants) genes, ftp://ftp.ncbi.nih.gov/refseq/release/plant/, and found that ABCD full structure transporters are present only in land plants but not in green algae. Such limited presence suggests a relatively young age for the ABCD full transporters.

The ABCB, ABCC, ABCE and ABCF families were found in all 79 selected eukaryotic genomes (Table S1), suggesting that members (genes) of these families are likely to be involved in conserved functionalities. By contrast, ABCA, ABCD and ABCG families seem to be completely absent in some clades (Table S1) suggesting they have been secondarily lost. Examples include the loss of ABCA family in many fungi, the loss of ABCA and ABCD families in apicomplexans (a major group of obligate parasites belonging to the alveolata), and the loss of ABCG full structure in alveolata, euglenozoa, metazoa and fornicata (Table S1).

In contrast to the gene loss in various genomes, ABC transporters have proliferated massively to more than 100 genes in some organisms such as the ciliate *T. thermophila*, the land plants *P. patens*, *A. thaliana* and *O. sativa* (Table S1). These expansions occurred mainly in groups with both half and full structures, namely ABCA, ABCB, ABCC, and ABCG families (Table S1). In addition, the non-transporter ABCF family also showed a significant expansion in some viridiplantae species (Table S1). The numbers of genes of the half transporter ABCD family (two genes on average) and the non-transporter ABCE family (one gene on average) appeared low and stable across these diverse organisms.

### Phylogeny of ABC transporter superfamily

ABC transporters are an ancient gene superfamily, members of which exist in almost all prokaryotes and eukaryotes. To date, however, the evolutionary origin of the various ABC transporters is still elusive. Considering various gene structures of ABC transporters and NBD sequence characteristics are the main criteria for ABC transporter classification, phylogenetic analyses of the ABC transporter superfamily are typically based on NBD sequences^8^,^14^,^20^,^21^. Therefore, we first extracted and aligned the NBD sequences of all the prokaryotic and eukaryotic genomes included in the present study and performed phylogenetic analyses. Since NBDs between different families have low identities (Table 1), there is a risk of making inaccurate multiple alignments. Therefore, we aligned the NBDs and TMDs sequences to the known HMM profile in the Pfam database (see Materials and Methods), this greatly improved the accuracy of alignment. Fasttree2, which has shown good performance in ABC transporter phylogenetic analysis^22^, was used to build the phylogenetic tree. It calculated the local support values using Shimodaira-Hasegawa test on the three alternate topologies (NNIs) around that split (http://meta.microbesonline.org/fasttree/). Based on the phylogenetic analysis, we inferred some clues as to the ancestral state of the NBD and TMD functional domains and to how the structure of ABC transporter superfamily might have evolved.

#### Eukaryotic ABC transporters fall into three major groups

**Figure 2:**
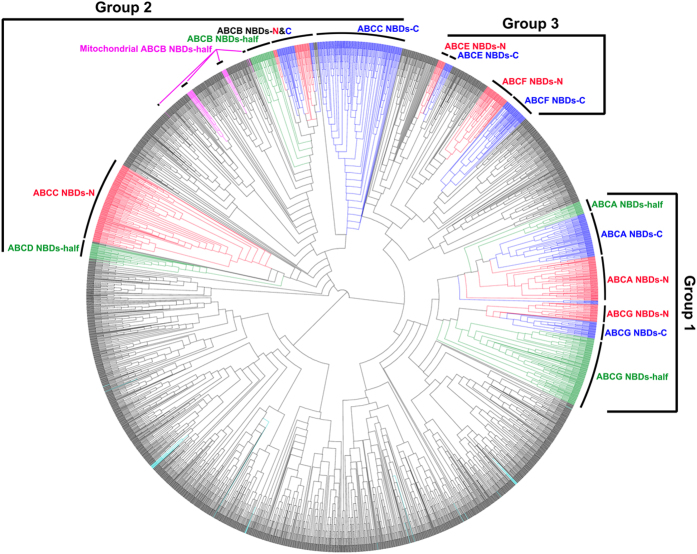
Phylogenetic tree of the NBDs. Green edge, eukaryotes NBDs from half structure transporters; Red edge, N-terminal NBDs from the ABC full or ABC2 structure transporters; Blue edge, C-terminal NBDs from the ABC full or ABC2 structure transporters; Pink edge, NBDs of a specific category of ABCB half structure transporters, may have originated by mitochondrial endosymbiosis; Cyan edge, eukaryotic ABC transporter genes with single NBD structureclustered in the prokaryotic NBD clades; Black edge, prokaryote NBDs. NBDs-half, eukaryote NBDs from half structure transporters; NBDs-N, N-terminal NBDs from the ABC full or ABC2 structure transporters; NBDs-C, C-terminal NBDs from the ABC full or ABC2 structure transporters. Group 1, Group 2 and Group 3 indicate three major clusters for the seven ABC transporter families.

**Figure 3:**
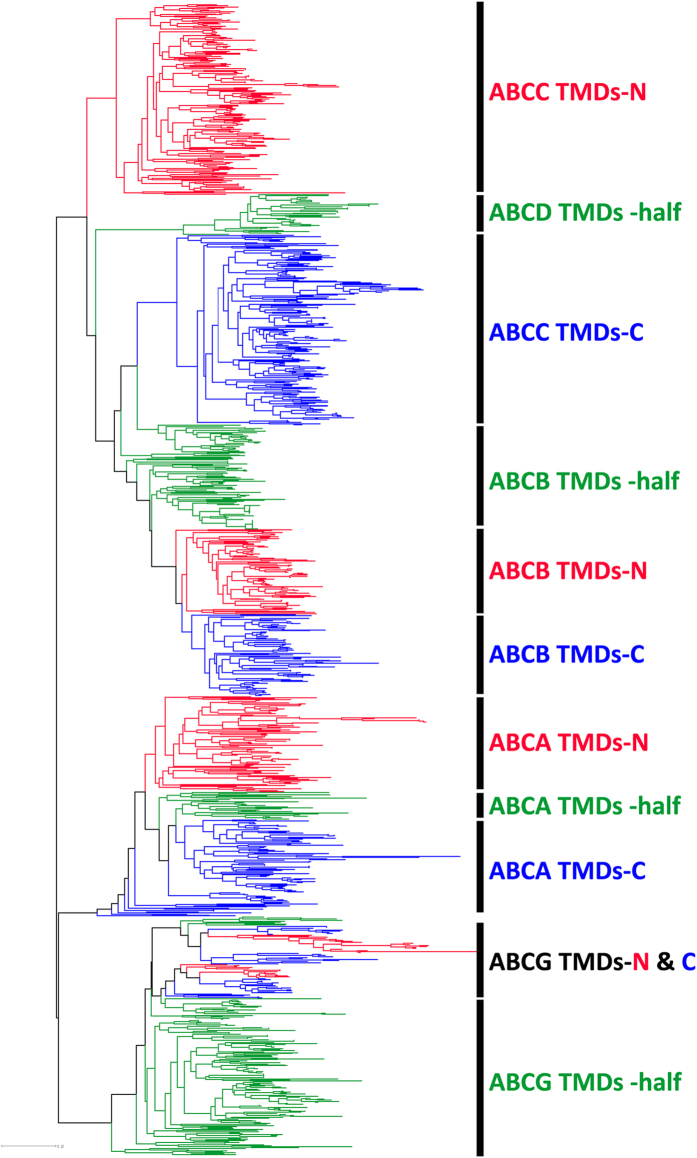
Phylogenetic tree of the eukaryotic TMDs. Green edge, eukaryote TMDs from half structure transporters; Red edge, N-terminal TMDs from the ABC full or ABC2 structure transporters; Blue edge, C-terminal TMDs from the ABC full or ABC2 structure transporters. Only TMDs from five eukaryotic ABC families (ABCA, ABCB, ABCC, ABCD and ABCG) were used to build the tree.

According to the structures shown in Fig. 1, the single and half structures contain one NBD whereas the full and ABC2 structures contain two NBDs at the N- and C-terminal, respectively. Most of the internal nodes, which are close to the root in the NBDs tree, have local support values range from 50% to 90% (see Materials and Methods). The topology of the phylogenetic tree revealed that the eukaryotic ABC transporter NBDs fall into three major groups (Fig. 2), The ABCA and ABCG NBDs are clustered together and form group 1; the ABCB, ABCC and ABCD NBDs, with some prokaryotes NBDs, form group 2; the non-transporters ABCE and ABCF NBDs also clustered with some prokaryotes NBDs forming group 3. Besides the NBD tree, the extracted TMD sequences of eukaryotic ABC proteins were also used to construct a phylogenetic tree. As ABCE and ABCF families do not contain TMD (ABC2 structure), these two families were excluded when constructing the TMD tree. The TMD tree is shown in Fig. 3, and its topology also supports the same three groups determined by the NBD tree. In addition, these three groups are consistent with previous studies that also divided ABC transporters into three classes based on NBD phylogeny^23^.

#### Group 1: ABCA and ABCG families

Compared to other families, the mean NBD sequence similarity is highest between ABCA and ABCG families (26.4%) (Table 1). NBDs and TMDs of these two families clustered together forming group 1 (Figs 2,3 and S1). They are associated with the clades of prokaryotic NBDs that are present at the basal position of group 1 in NBDs tree (Fig. 2 and S1). In addition to the overall conservation of amino acid sites across the entire ABC transporter superfamily, this group has some unique conserved amino acid sites in its NBD domain, such as the Q loop and the Pro (P) site of the Walker B motif (Figure S2). Therefore, the close relationship of these two families indicated the NBDs of these two families shared a common ancestral NBD.

Most of the C-terminal NBDs (NBDs of the C-terminal half of the protein) of the ABCA full structure transporters showed a closer phylogenetic relationship to the half structure NBDs than to their N-terminal NBDs (NBDs of the N-terminal half of the protein) (Fig. 2 and S1). This topology also appeared in their phylogenetic relationship of TMDs (Fig. 3). Analyses of the conserved regions of their NBDs revealed nearly identical protein motifs among the half structure NBDs and the full structure N- and C-terminal NBDs (Figure S2). Interestingly, the topology of the ABCG phylogenetic tree showed a different pattern, in which the tree supports a closer relationship between the N- and C-terminal NBDs or TMDs of ABCG full structure transporters, but both diverge more from the half structure NBDs or TMDs (Figs 2,3 and S1).

#### Group 2: ABCB, ABCC and ABCD families

The NBDs and TMDs in these three families formed a large group in the phylogenetic trees (Figs 2 and 3). Their NBDs sequences shared conserved amino acid sites in their motifs including the Q loop, the Gln (Q) site in the signature motif and the Ala (A) site of the Walker B motif (Figure S2). Similar to Group 1, the NBD sequences showed high similarities among the three families in Group 2 (30% between the ABCB and ABCC families; 25% between the ABCB and ABCD families; 24% between the ABCC and ABCD families. Table 1).

All NBDs of the ABCB transporters formed a single, divergent clade in Group 2 (Fig. 2 and S3) and a similar topology was found in the TMD tree (Fig. 3). The N- and C-terminal NBDs or TMDs of ABCB full structure transporters are clustered together, similar to that of the ABCG family (Figs 2,3 and S3). However, the N- and C-terminal NBDs did not form a clade independently that is different from the ABCG full structure transporters (Figure S3), which is likely due to the high similarity between the N- and C-terminal NBD sequences (Figure S2). The NBDs of ABCB half structure transporters can be divided into two categories. One is a small set of genes located on mitochondrial membranes, the location of which suggests a potential origination from mitochondrial endosymbiosis (Fig. 2 and S3, taxa in pink font; for details, see below). The other contains the majority of the ABCB half transporter genes, and bears a close relationship with the ABCB full structure NBDs (Fig. 2 and S3).

The topology of the ABCC family indicates that the C-terminal NBDs of the ABCC full structure transporters possess a closer relationship to the NBDs or TMDs of the ABCB transporters than to its own N-terminal NBDs or TMDs (Figs 2,3 and S3). Furthermore, the protein motifs between the N- and C-terminal NBDs show some significant differences. In the Walker B motif, for example, the N-terminal NBDs showed conserved sites with a DDPLA pattern, whereas the C-terminal NBDs showed a DEATA pattern (Figure S2). These results suggest a high divergence of the N-terminal NBDs from the C-terminal NBDs in the ABCC family, which strongly implies possible independent origins of the NBDs at the two terminals.

**Figure 4:**
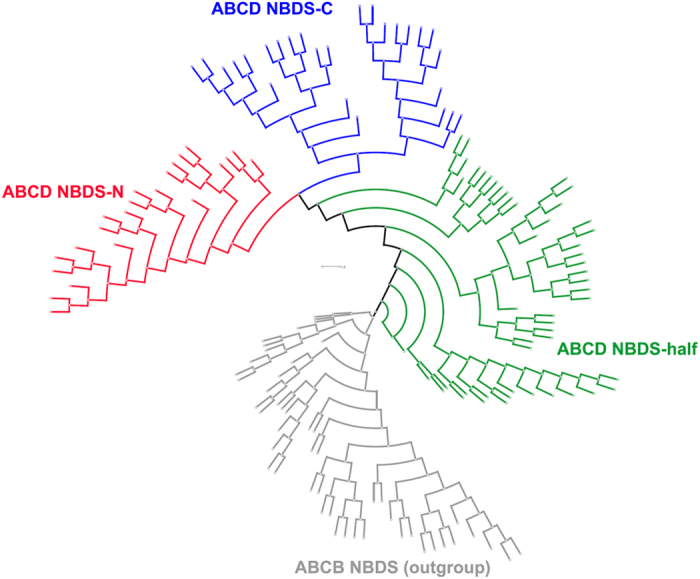
Phylogenetic tree of the plant ABCD NBDs. Phylogenetic tree constructed using MEGA 5.0 using the N-J method with JTT model, 1000 bootstraps. ABCB NBDs were used to as the outgroup. Green, NBDs from half structure ABCD transporters; Red, N-terminal NBDs from the ABCD full structure transporters; Blue, C-terminal NBDs from the ABCD full structure transporters; Gray, ABCB NBDs.

In the ABCD family, the half structure appears to be shared by almost all eukaryotes, however, its full structure was only found in land plants (described above, Table S3). As shown in Figs 2 and S3, the ABCD half structure NBDs form a single branch and are clustered with the ABCC full structure transporters N-terminal NBDs. In contrast to the NBD tree, the ABCD half structure TMDs are clustered with the large clade of ABCB and ABCC C-terminal TMDs in the TMD tree (Fig. 3). However, the locations of the NBDs of the ABCD family in the phylogenetic tree are not always consistent among different studies. Some previous analyses showed that the ABCD NBDs were localized in the basal parts of the ABCB and ABCC clades rather than being clustered with the N-terminal NBDs of the ABCC full structure transporters^8^,^14^. Because only land plants have the ABCD full structure, we analyzed the ABCD family in land plants separately and found that in the ABCD full structure transporters, their N- and C-terminal NBDs showed closer relationships with each other relative to ABCD half structure NBDs (Fig. 4). This topology appears the same as that of ABCG family. We also noticed that there is a small clade of prokaryotic half structure NBDs in each of the basal positions of the ABCB NBD cluster, the ABCC full structure N-terminal NBDs cluster, the ABCC full structure C-terminal NBDs cluster and the ABCD NBD cluster (Fig. 2 and S3).

#### Group 3: ABCE and ABCF families

Members of ABCE and ABCF have an ABC2 structure (Fig. 1), and do not function as transporters. Like the full structure ABC transporters, we also independently extracted the N-terminal and C-terminal NBDs of ABCE and ABCF families for phylogenetic analysis. As shown in Fig 2, the N- and C-terminal NBDs form a separate clade and are clustered with the N- and C-terminal NBDs of archaea ABC2 structure ABC proteins in the ABCE family, respectively (Fig. 2 and S4). In that respect, the ABCE ABC2 structure genes may only exist in archaea but not bacteria, suggesting the ABC2 structure is likely to have originated after the divergence of bacteria and archaea. The ABCF family has a similar topology to the ABCE family. The N- and C-terminal NBDs of the ABCF family appear to be clustered closely with the bacterial ABC2 structure N- and C-terminal NBDs, respectively (Fig. 2 and S4). This topology suggests that this kind of ABC2 structure (ABCF-like) originated in bacteria.

#### Two special gene categories

In the phylogenetic tree, there are two special categories of ABC genes. One comprises some ABCB half structure members (Fig. 2 and S3, taxa in pink font); NBDs of these genes do not cluster with other ABCB NBDs (the ABCB NBDs clade) but instead are nested within the prokaryote half structure NBDs clades which are near the ABCB clade. This topology suggested that these genes have a closer relationship with the prokaryote ABC proteins, and may be initially generated by horizontal gene transfer (HGT) either from prokaryotes to eukaryotes, or from organelles such as mitochondria and chloroplasts that evolved from prokaryote ancestors. We further found that some of these genes were experimentally verified with a mitochondrial membrane localization, such as human ABC transporters ABCB6 and ABCB7^7^,^24^, the yeast protein ATM1^25^, and the *Arabidopsis* Sta1 ABC proteins^13^,^26^ (Table 2). Additionally, using a combination of de novo prediction tools (see Materials and Methods), gene ontology (GO) annotation and BLAST searches, we found that most of these genes show predicted mitochondrial membrane localization (Table 2). Therefore, it seems likely that the rise of the ancestors of these genes accompanied the mitochondrial endosymbiosis event during which genes from the mitochondrion genome were integrated into the nuclear genome^27^. This is also in accordance with a previous hypothesis that eukaryotic organelle NBDs originated through endosymbiosis^28^.

The second special gene category is the single NBD structure ABC proteins in eukaryotes, which could not be assigned to any of the seven main eukaryotic ABC transporter families. As shown in Fig. 2 (taxa in cyan), these NBDs were clustered in the prokaryotic NBDs clades. It is highly likely that such eukaryotic NBDs originated by lateral gene transfer from prokaryotes to eukaryotes.

### TMDs features of ABC transporter superfamily

**Figure 5:**
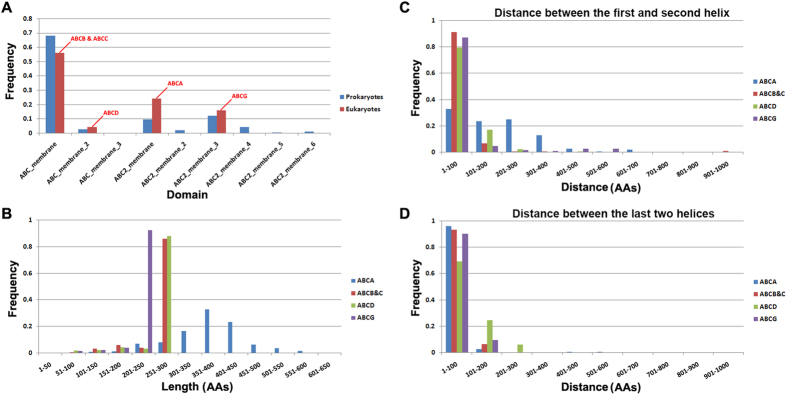
TMD classes and its length and helices characters. A, the classes of TMDs, a total of nine TMD classes, only four found in eukaryotes; B, the length distribution of eukaryotes TMDs; C, distribution of distance between the first and second helix in each ABC transporter family; D, distribution of distance between the last two helices in each ABC transporter family.

Besides NBD, TMD is the other domain of ABC transporters. In the Pfam database^29^, TMDs of ABC transporters are classified into nine classes, including ABC_membrane (PF00664), ABC_membrane_2 (PF06472), ABC_membrane_3 (PF13748), ABC2_membrane(PF01061), ABC2_membrane_2(PF12679), ABC2_membrane_3(PF12698), ABC2_membrane_4(PF12730), ABC2_membrane_5(PF13346), and ABC2_membrane_6(PF06182). In our study, all nine classes were found in prokaryotes (Fig. 5A), the most frequently occurring one being the ABC_membrane class, followed by the ABC2_membrane_3 and ABC2_membrane classes. In eukaryotes, only four classes were found: ABC2_membrane TMDs in the ABCA family, ABC_membrane TMDs in the ABCB and ABCC families, ABC_membrane_2 TMDs in the ABCD family and ABC2_membrane_3 in the ABCG family (Fig. 5A). ABC_membrane also appears most frequently (Fig. 5A). Compared to NBDs in eukaryotes, the sequences of four eukaryotic TMD classes are highly divergent, and show low sequence conservation within and between classes (Figure S5). Such results are likely to correspond to the specific functions of TMDs that need to bind different transporting substrates. Among the four classes of eukaryotic TMDs, the TMDs of the ABCA family are the longest, many having lengths between 301 and 500 amino acids (AAs) (Fig. 5B). The TMDs of the ABCG family are the shortest with lengths between 201 and 250 AAs. The TMDs of the ABCB, ABCC and ABCD families have similar lengths, ranging from 251–300 AAs (Fig. 5B). The TMD domain usually contains several transmembrane helices. A slight difference was found between TMDs of the ABCA family and other families, the former possessing a longer distance between the first and second transmembrane helices (Figure S6).

We analyzed the distances between the first and second transmembrane helices in the TMDs of each eukaryotic ABC family and found in more than 65% of ABCA family TMDs this distance was more than 100 AAs, whereas in more than 80% (nearly 80% of the ABCD family) of other families it was less than 100 AAs (Fig. 5C). Because the ABCG transporter showed a reverse domain organization compared to other families (Fig. 1), we speculate that the transmembrane helices are reversed in ABCG TMDs. Therefore, we analyzed the distance of the last two transmembrane helices in the TMDs of each family. We did not observe any significant difference among these four families in the distance between the last two transmembrane helices, this being less than 100 AAs in the majority (Fig. 5D).

## Discussion

Understanding how genes originate and evolve is crucial to explaining the genetic basis for novel gene function. Gene structural changes are an important aspect of the evolutionary process. It has been shown in many cases that gene duplication (which could supply the raw fusion materials) and subsequent fusion is a main route of origin of new gene structure^30^,^31^,^32^,^33^,^34^. The ABC transporter system catalyzes influx and/or efflux of many substrates using ATP hydrolysis for energy^1^,^2^,^35^,^36^, and thus plays an important role not only in general metabolism but also in specific pathways such as resistance to drugs and toxins^37^. In order to have a transporting function, ABC transporters typically need two NBD subunits and two TMD subunits, which means a single structure gene and a half structure gene should form a tetramer and a dimer, respectively. In a full structure transporter, however, the gene product can possess a full function and therefore does not need a partner to form a tetramer. Thus, a full structure transporter should have advantages in terms of efficiency of transcription and translation, as well as transport quantity control. Prokaryotic ABC proteins are mainly single and half structures and are expected to function as a tetramer or dimer, and different ABC transporters could form an operon to control and balance the components^38^,^39^,^40^,^41^,^42^. In eukaryotes, ABC transporter proteins are mainly half and full structures, and thus similar to the operon in prokaryotes. From simple gene structures in prokaryotes to diverse and complex gene structures in eukaryotes, a key step is gene fusion that involves mechanisms such as DNA recombination, DNA mutation, transposition and retroposition^33^. Presumably, a single TMD and NBD can be fused to a half structure transporter, two different NBDs can be fused to the ABC2 structure, and two half structure transporters could be fused to the full structure transporter.

### Clues for ancestral state of ABC transporter superfamily

From our phylogenetic tree (Fig. 2), it is clear that the ancestral NBD emerged in or before the last universal common ancestor. Since there is no outgroup in the NBD phylogenetic tree, the most ancestral state of the NBDs is difficult to resolve. Nonetheless, our present work can shed light on the root of the NBDs.

In our phylogenetic analysis, a specific category of eukaryotic ABCB half structure genes had their NBDs clustered with prokaryotic half structure NBDs. This pattern is different from all others (Fig. 2 and S3). We further found that most genes in this class are localized in the mitochondrial membrane (Table 2). Such results suggest a more ancient origin of NBDs and they may have originated as a consequence of endosymbiosis.

Eukaryotic ABCF genes possess an ABC2 structure and are clustered with prokaryotic ABC2 structure genes (Fig. 2 and S4). These ABCF genes do not function as transporters. Instead, they are involved in translational regulation, for example, the yeast translation elongation factor (EF-3) gene^43^, and GCN20 gene responsible for the activation of eIF-2 alpha kinase^44^. The prokaryotic ABC2 structure genes that clustered with eukaryotic ABCF genes are functionally related to antibiotic resistances (ARE), such as the MsrA and Vga proteins involved in resistance to virginiamycin and erythromycin^45^,^46^, respectively. In addition, few conserved amino acid sites were found in the region between the Walker A and signature motifs of ABCF genes compared to other families (Figure S2). If the overall mutation rate is similar among all the ABC families, ABCF NBDs are likely to have experienced the longest divergence time, and thus may be the most ancestral state of all NBDs.

**Figure 6:**
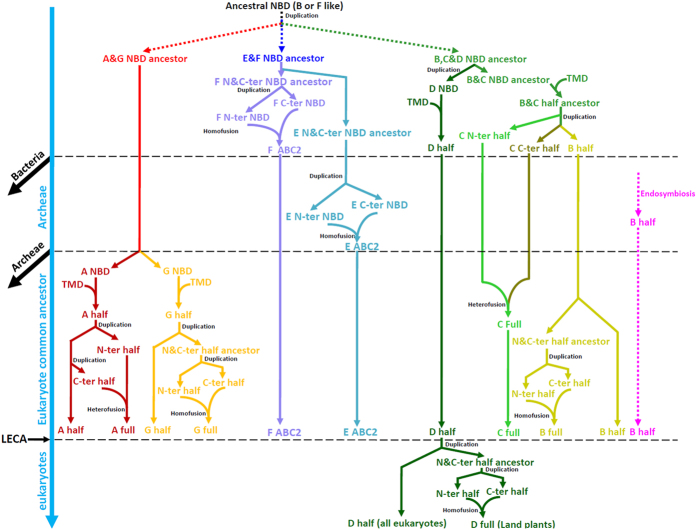
Proposed evolutionary trajectories of the ABC transporter genes. A-G, the seven families of ABC transporters; half, half structure transporter; full, full structure transporter; ABC2, ABC2 structure transporter. C-ter, C-terminal; N-ter, N-terminal.

In short, we suggest the most ancestral state of NBDs is either ABCB- or ABCF-like (Fig. 6). It is clear that the eukaryotic ABC transporters fall into three groups (Figs 2 and 3). These three groups may each represents a NBD ancestor that originated by multiple duplications and divergence from the most ancestral NBD (Fig. 6).

### The origin of ABC half structure transporters: fusions of TMD and NBD

Domains are conserved regions of proteins and independent units of protein structures. In the course of evolution, different combinations or fusion of domains can lead to novel genes and protein structures. ABC transporter genes also show diverse protein structures through various domain combinations. In our study, all of the seven ABC transporter families are present in a diverse set of eukaryotic clades (Table S1), indicating that they originated in the LECA or earlier. It is noteworthy again that eukaryote ABC transporter genes encode multiple domains but most prokaryotic ABC transporter genes encode a single domain (only TMD or NBD). Therefore, it is likely that the single domain genes underwent a series of gene fusion events to form ABC half transporters before the LECA evolved.

#### ABCA and ABCG families

ABCA and ABCG NBDs are clustered together and form Group 1 as shown in Fig. 2, suggesting that NBDs in these two families share a common NBD ancestor (Fig. 6). Unlike others, NBD precedes TMD in ABCG transporters, in a reversed orientation to that of the ABCA-D transporters (Fig. 1). We hypothesize that the ABCG family originated either by a fusion of a single TMD with a NBD or from a copy of the central region of a pre-existing ABCA (the ABCA and ABCG NBDs are clustered together in the NBD tree) full structure transporter^8^. The ABCA family shows a TMD distinct from that of the ABCG family (Fig. 5 and S6) with regard to the following characteristics: most ABCA TMDs are significantly longer than ABCG TMDs and exhibit a transmembrane helix pattern with a distance between the first and second helix that is longer than in the ABCG family (Fig. 5). Therefore, the ABCA and ABCG TMDs are seemingly two different classes (Fig. 5), suggesting the ABCG family TMD is not a copy of ABCA TMD. The disparities between the ABCA and ABCG TMD structures hint that their TMDs may have had different origins. This contrast with the common origin of the NBD and further suggests that ABCA and ABCG half transporters originated through independent fusions of NBDs and TMDs rather than from a copy of the central region of a pre-existing ABCA full structure transporter.

To determine the time of origin of the ABCA and ABCG half structure transporter, we checked the structure of the prokaryotic genes clustered in the basal position of the ABCA and ABCG NBDs (Fig. 2 and S1), and found that the prokaryotic genes have a single structure. This provides evidence that ABCA and ABCG half structure transporters originated after the origin of eukaryotes (Fig. 6).

#### ABCB, ABCC and ABCD families

These three families form Group 2 in the NBD phylogenetic tree (Fig. 2), suggesting NBDs of these families share a common NBD progenitor. Eukaryotic NBDs in this large group can be divided into four clades including ABCB NBDs, ABCC full structure transporter C-terminal NBDs, ABCC full structure transporter N-terminal NBDs, and ABCD NBDs. In contrast to ABCA and ABCG families characterized with the whole clade (ABCA + ABCG) clustering with prokaryotic ABC genes (Figure S1), each of these four clades are clustered with a small set of prokaryotic ABC genes in the basal positions (Figure S3), and these prokaryotic ABC genes are half structure (not shown) rather than single structure transporters. This suggests that the progenitors of these four clades had a half structure, and potentially originated in bacteria. However, there should not be four independent fusion of TMD and NBD because only two classes of TMDs were found in these three families (Fig. 5A), including ABC_membrane TMDs in the ABCB and ABCC families and ABC_membrane_2 TMDs in the ABCD family. The TMDs classes suggest there is an intermediate NBD ancestor of the ABCB and ABCC families. Therefore, the most likely scenario is the ABCD NBDs and the NBD ancestor of the ABCB and ABCC families diverged first, then each fused with the TMD independently (Fig. 6). Then, the NBD ancestor of the ABCB and ABCC families divided into the ABCC N-terminal half, the ABCC C-terminal half and the ABCB half (Fig. 6).

### Full transporters originated by fusion of half transporters

A more typical structure in the ABC transporter gene superfamily is the full transporter that encodes four domains in one gene (Fig. 1). Full structure transporter members of the ABCA, ABCB, ABCC and ABCG families are present in most of the investigated eukaryotic genomes (Table S1); thus, each of the ABC full transporter ancestors of these families originated in or before the LECA (Fig. 6). Because the full structure of the ABCD family was found only in land plants (Table S1 and Table S3), it most likely originated from the last common ancestor of all land plants (Fig. 6).

A characteristic of the gene fusion mechanism is that it can only gain one or more domains at the termini of a gene, which does not lead to domain insertions from one gene to another. Full structure ABC transporters typically contain two half structures. Therefore, the full structure transporter ancestor may be attributed to gene fusions of two half structure transporters. In a functional context, such full structure transporter genes possess advantages for better control of the exchange across a membrane than half structure transporter or single structure ABC transporter genes. In addition, full structure transporters are presumably more tolerant to mutations. Here, based on the topologies of the NBD and TMD trees (Figs 2,3, S1 and S3), we hypothesize that the four eukaryotic full structure transporters could have arisen in two different ways: heterofusion and homofusion.

#### Heterofusion of ABCA and ABCC full structure transporter

Heterofusion defined as one of the N- and C-terminal NBDs in full structure transporters showed firstly clustered with NBDs in half structure transporters. In the ABCA family, the C-terminal NBDs or TMDs of full structure transporters are closer to the half structure NBDs or TMDs, indicating a more distant relationship with the N-terminal NBDs or TMDs of full structure transporters (Figs. 2,3 and S1). This suggests that the ABCA full structure transporter originated by the heterofusion mode (Fig. 6). In the ABCC family, the C-terminal NBDs or TMDs of the ABCC full structure transporters were closer to those of the ABCB family NBDs or TMDs than to its own N-terminal NBDs or TMDs (Figs. 2,3 and S3). Consistent with this result, protein sequence motif analyses also revealed that the C-terminal NBDs of ABCC full structure transporters differ from the N-terminal NBDs, being more similar to the NBDs of ABCB, such as the Walker B motif (Figure S2). Taken together, these results strongly suggest that the N- and C-terminal domains of the ABCC family are distinct from each other, and the ABCC full structure transporters may have originated from fusions of two divergent half transporters (Fig. 6). The heterofusion mode suggests that two rounds of gene duplications of the ABC half structure transporter occurred (Fig. 6), and two half transporters each come from a round of duplication independently accumulated mutations that led to the sequence divergence, and finally fused together (Fig. 6).

#### Homofusion of ABCB and ABCG full structure transporter

Homofusion defined as the N- and C-terminal NBDs in full structure transporters firstly clustered together, and then clustered with NBDs in half structure transporters. In the ABCB family, the phylogenetic trees showed that the N- and C-terminal NBDs or TMDs of the ABCB full structure transporters first clustered together and then clustered with the NBDs or TMDs of ABCB half structure transporters (Figs. 2,3 and S3). Notably, the sequence identities between the N- and C-terminal NBDs of the ABCB were higher than that of any other families and the motifs of the NBDs were almost identical (Figure S2). These results clearly suggest that the ABCB full transporter arose by fusions of two recently duplicated ABCB half transporters (Fig. 6), and yielded high identity between the two NBDs in the progenitor of the ABCB full structure transporter. In the ABCG family, the topologies of the NBDs and TMDs phylogenetic trees suggest a close relationship between the N- and C-terminal NBDs or TMDs of ABCG full structure transporters (Figs. 2,3 and S1). However, the motifs of the N-terminal and C-terminal NBDs of ABCG full structure transporters did not shown high identity like the ABCB family (Figure S2). It is likely that the N-terminal half and the C-terminal half fused after a relatively long time of duplication, and underwent homofusion (Fig. 6). The homofusion mode implies that gene fusion occurred shortly after a round of gene duplication of the ABC half structure transporter. Under that scenario, the two half structures showed high sequence identities in the full structure transporters.

### Origin of non-transporter ABCE and ABCF families: homofusion of two single NBDs

ABCE and ABCF have the ABC2 structure (Fig. 1), and the N-terminal and C-terminal NBDs clustered closely in both families (Fig. 2). It is noteworthy that the NBDs of ABCE and ABCF transporters clustered closely with each other in the phylogenetic tree (Fig. 2), suggesting a common ancestor for these two families (Fig. 6). In addition, the ABCE family NBDs clustered only with NBDs in archaea, whereas the ABCF family NBDs clustered with NBDs of bacteria. Thus, our findings suggested that the ABCE ABC2 structure originated in archaea after it diverged from bacteria, and the ABCF ABC2 structure originated before divergence of bacteria and archaea (Fig. 6). There is a clade of bacterial single structure NBDs that branches basally in ABCE family NBDs, including the archaea ABC2 structure NBDs (Figure S4). This suggests that the eukaryotic ABCE and prokaryotic ABC2 structure NBDs had a bacterial single structure NBD ancestor, and thus the ABCE ABC2 structure gene also originated by homofusion of two duplicated NBDs rather than by duplication of the ABCF ABC2 structure gene (Fig. 6).

## Conclusion

Gene duplication is a major mechanism for producing new genes^47^. It not only contributes to the expansion of gene families, but also supplies resources for new protein structures. DNA mutation frequently causes the divergence of duplicated sequences, which can further provide genetic materials for innovation. Gene fusions with different combinations of domains can also result in novel gene and protein structures and therefore new functions. Here, in order to trace the structural evolution of the eukaryotic ABC transporter gene superfamily, we constructed a model to elucidate potential evolutionary trajectories (Fig. 6). Overall, the most ancestral NBD duplicated and diverged into three NBDs, and these represent the NBD ancestors of three major groups. In the half transporters origin process, there are at least four times the number of fusions with TMD and NBD, and ultimately formed four half transporters. One of the four half structure transporters diverged into ABCC N-terminal, C-terminal and ABCB half structure transporters. The full structure transporters originated by two patterns: heterofusion and homofusion. The ABCA, ABCB, ABCC and ABCG full structure transporters originated before the LECA, and the ABCD family full structure transporter originated in the ancestor of land plants. The ABCF ABC2 structure originated before the divergence of bacteria and archaea, and the ABCE family ABC2 structure originated in archaea. Finally, each family expanded in the eukaryotes and prokaryotes.

## Materials and Methods

### Genomes sources

The organisms were selected by the following principles: 1) fully sequenced genome; 2) representatives from each of the three domains of life. A total of 79 representative eukaryotes from nine categories were chosen in our analyses (shown in Table 1), and the sequences were retrieved from the OrthoMCL database (http://orthomcl.org/orthomcl/) which collected many eukaryote genome and predicted proteome sequences. 2302 prokaryote genomes were retrieved from the NCBI genome FTP site (ftp://ftp.ncbi.nlm.nih.gov/genomes/Bacteria/). To investigate the ABCD genes in land plants, we also retrieved the plant sequences from the NCBI Refseq database (ftp://ftp.ncbi.nih.gov/refseq/release/plant/).

## Identification of ABC transporters

To identify the ABC transporters in a genome, the *Tetrahymena thermophila* ABC transporter sequences identified in our previous study^14^ was used as the query to do the BALSTP searches against the predicted proteomes using an E-value cutoff of 10. This process ensured all the ABC transporter candidates could be retrieved, including some non-ABC proteins, for example the structural maintenance of chromosomes (SMC) family proteins. Then, all the BLASTP candidates were run for another round of profile-based domain search in the Pfam database^29^. Proteins with a significant Pfam NBD hit or TMD hit were regarded as potential ABC transporter family genes. For eukaryotes, classification of the identified ABC proteins was performed by searching the NCBI non-redundant protein database, determined by the annotation of BLASTP high rank hits; the majority of the putative ABC proteins (about 95%) could be assigned to one of the seven families of eukaryote ABC transporters according to BLASTP hits annotations and domain characteristics. It is noteworthy that some of eukaryotic genomes contained alternative splicing protein products, which led to an increase in the number of ABC transporters genes.

## Phylogenetic analysis

We did not include all the genomes in the phylogenetic analyses for the following reasons: 1) only a part of the genomes could represent the structural diversity of the ABC transporters; 2) gene mis-predictions are common in eukaryotes as gene prediction is more difficult than in prokaryotes; 3) although large phylogenetic trees could be built, they are difficult to visualize. Based on these reasons, we selected 16 eukaryotic genomes and 55 prokaryotic genomes to construct the phylogenetic tree (Table S4). The eukaryotes were chosen mainly on the basis of the group to which they belong and the level to which they have been studied, i.e. if they are model organisms. The prokaryotes were chosen mainly by their taxonomy, the 55 prokaryotes representing 55 different higher taxa.

Because the ABC gene varies in length, NBDs and TMDs sequences were used to construct the phylogenetic tree in our analyses. The NBDs sequences were extracted based on the position of the Pfam domain search results using a custom Perl script, and a total of 3818 NBDs and 1579 TMDs were retrieved. To do the alignment, we first extracted the hmm profile of NBDs in the Pfam database and then aligned it using the hmmalign program^48^. The command line of hmmalign was “hmmalign –amino –informat FASTA –outformat PSIBLAST −o output_file NBD.hmm (or TMD.hmm) NBD_sequences.fa”. We also compared the hmmalign results to MAFFT^49^ alignment, and the hmmalign results were significantly better than MAFFT in this large sequence alignment. Because there were nine hmm profiles for TMDs, each class of TMDs was first aligned to its own hmm profile to generate nine alignments, and these nine alignments were then re-aligned using the MUSCLE software^50^. TrimAl^51^ was use to remove those columns with too many gaps with command line –gt option 0.8, thus removing columns comprising more than 20% of gaps. Phylogenetic analyses were constructed using the approximately maximum likelihood based FastTree software (version 2) with command “FastTree −mlacc 2 -slownni alignment_sequences” and default amino acid substitution model. Fasttree can handle large alignments and ran 100–1,000 times faster than PhyML 3.0 or RAxML 7^52^ with comparable accuracy to PhyML 3.0 (http://meta.microbesonline.org/fasttree/#Performance). The “-mlacc and -slownni” made the maximum-likelihood NNIs more exhaustive and may have slightly increased the accuracy (see http://meta.microbesonline.org/fasttree/#Performance). Figure 7 shows the raw tree with local support values (the raw tree Newick files are available upon request).

### Sequences conservation and transmembrane helix analysis

The NBDs sequence identities were calculated based on the alignment using a custom Perl script. Sequence conservation was analyzed using the WebLogo^53^. The WebLogo required aligned sequences, the NBDs alignment for each set was extracted from the whole NBDs alignment, and the TMDs alignments were performed in a way similar to that of the NBDs using hmmalign program (see above). To infer the transmembrane helices, we examined the transmembrane topology of all eukaryote ABC half transporters using the TMHMM Server^54^ and TopPred (http://mobyle.pasteur.fr/cgi-bin/portal.py#forms::toppred).

## Additional Information

**How to cite this article**: Xiong, J. *et al.* Tracing the structural evolution of eukaryotic ATP Binding cassette transporter superfamily. *Sci. Rep.* **5**, 16724; doi: 10.1038/srep16724 (2015).

## Supplementary Material

Supplementary Information

## Figures and Tables

**Table 1:** Mean identities among the NBD sequences of seven ABC transporter families.

Family	ABCA	ABCB	ABCC	ABCD	ABCE	ABCF	ABCG
ABCA	38%	25%	23%	21%	22%	22%	26%
ABCB	–	47%	30%	25%	22%	24%	25%
ABCC	–	–	37%	24%	19%	21%	22%
ABCD	–	–	–	41%	20%	21%	21%
ABCE	–	–	–	–	37%	20%	21%
ABCF	–	–	–	–	–	31%	23%
ABCG	–	–	–	–	–	–	37%

**Table 2:** Subcellular location of the sepecific ABCB gene category.

Species	Category	Gene ID	Description^a^	De novo prediction^b^	GO annotation^c^
*Arabidopsis thaliana*	Viridiplantae	NP_200635.1	ABC transporter of the mitochondrion 3, sta1 gene	mitochondrion	mitochondrion* (Rocio *et al.* 2001)
*Arabidopsis thaliana*	Viridiplantae	NP_194591.1	ABC transporter B family member 24, mitochondrial, AtATM2 gene	mitochondrion	mitochondrion* (Rocio *et al.* 2001)
*Arabidopsis thaliana*	Viridiplantae	NP_567813.1	ABC transporter B family member 23, mitochondrial, AtATM1 gene	mitochondrion	mitochondrion* (Rocio *et al.* 2001)
*Arabidopsis thaliana*	Viridiplantae	NP_196011.1	ABC transporter B family member 29, chloroplastic	chloroplast	chloroplast
*Caenorhabditis elegans*	Metazoa	NP_001022812.1	heavy metal tolerance factor 1,Hmt1	/	mitochondrion
*Caenorhabditis elegans*	Metazoa	NP_001021830.1	ABC7 protein	mitochondrion	mitochondrion
*Danio rerio*	Metazoa	XP_005160347.1	ATP-binding cassette sub-family B member 6, mitochondrial isoform X1	endoplasmic reticulum	mitochondrion
*Danio rerio*	Metazoa	XP_005167575.1	ATP-binding cassette sub-family B member 6, mitochondrial	/	mitochondrion
*Danio rerio*	Metazoa	XP_694879.2	ATP-binding cassette sub-family B member 7, mitochondrial	mitochondrion	mitochondrion
*Dictyostelium discoideum* AX4	Amoebozoa	XP_629496.1	Similar to ATP-binding cassette sub-family B member 7, mitochondrial-like [Oryzias latipes]	mitochondrion	mitochondrion
*Dictyostelium discoideum* AX4	Amoebozoa	XP_639260.1	Similar to metal ABC transporter permease [Achromobacter xylosoxidans]	/	/
*Drosophila melanogaster*	Metazoa	NP_650503.1	heavy metal tolerance factor 1,Hmt1	/	mitochondrion
*Drosophila melanogaster*	Metazoa	NP_728642.2	ABCB7	mitochondrion	/
*Giardia lamblia* ATCC 50803	Fornicata	XP_001710177.1	Multidrug resistance ABC transporter	/	/
*Homo sapiens*	Metazoa	NP_005680.1	ATP-binding cassette sub-family B member 6, mitochondrial	/	mitochondrion* (Mitsuhashi *et al.* 2000)
*Homo sapiens*	Metazoa	NP_004290.2	ATP-binding cassette sub-family B member 7	mitochondrion	mitochondrion* (Csere *et al.* 1998)
*Leishmania major* strain Friedlin	Euglenozoa	XP_001685635.1	Similar to mitochondrial ABC transporter ATM [Angomonas deanei]	mitochondrion	mitochondrion
*Monosiga brevicollis* MX1	Choanoflagellida	XP_001745205.1	Similar to ATP-binding cassette sub-family B member 7, mitochondrial-like [Hydra vulgaris ]	mitochondrion	mitochondrion
*Monosiga brevicollis* MX1	Choanoflagellida	XP_001749749.1	Similar to heavy metal tolerance protein precursor [Brucella sp. BO2]	/	mitochondrion
*Monosiga brevicollis* MX1	Choanoflagellida	XP_001749388.1	Similar to ATP-binding cassette sub-family B member 10, mitochondrial isoform 3 [Dasypus novemcinctus]	endoplasmic reticulum	mitochondrion
*Mus musculus*	Metazoa	NP_076221.1	ATP-binding cassette sub-family B member 6, mitochondrial	/	mitochondrion* (Krishnamurthy *et al.* 2006)
*Mus musculus*	Metazoa	NP_033722.1	ATP-binding cassette sub-family B member 7, mitochondrial	mitochondrion	mitochondrion* (Sakaino *et al.* 2009)
*Plasmodium falciparum* 3D7	Alveolata	XP_001350233.1	heavy metal transporter family	mitochondrion	/
*Plasmodium falciparum* 3D7	Alveolata	XP_001348629.1	heavy metal transport family	/	vacuolar membrane
*Saccharomyces cerevisiae* S288c	Fungi	NP_014030.1	ATP-binding cassette Fe/S cluster precursor transporter ATM1	mitochondrion	mitochondrion* (Kispal *et al.* 1999)
*Schizosaccharomyces pombe*	Fungi	NP_588371.3	vacuolar heavy metal tolerance protein, Hmt1	endoplasmic reticulum	fungal-type vacuole membrane
*Schizosaccharomyces pombe*	Fungi	NP_594288.2	mitochondrial ABC family iron transporter Atm1	mitochondrion	mitochondrion
*Tetrahymena thermophila* SB210	Alveolata	XP_001030428.1	ABC transporter family protein	mitochondrion	mitochondrion
*Trypanosoma brucei*	Euglenozoa	XP_829749.1	Similar to mitochondrial ABC transporter ATM [Angomonas deanei]	mitochondrion	mitochondrion
*Trypanosoma brucei*	Euglenozoa	XP_829749.1	Similar to mitochondrial ABC transporter ATM [Angomonas deanei]	mitochondrion	mitochondrion

^a^Sequence description by BLASTP similarity searches.

^b^Subcellular localization prediction by TargetP, Predotar or WoLF PSORT.

^c^Gene ontology cellular component annotations by Goanna (http://agbase.msstate.edu/cgi-bin/tools/GOanna.cgi).

^*^Localization supported by reference (experimental data).
