# Supplementary material for: Tracing the structural evolution of eukaryotic ATP binding cassette transporter superfamily
Source: Sci Rep. 2015 Nov 18;5:16724. doi: 10.1038/srep16724 (PMC4649718; doi:10.1038/srep16724)
Supplement: Supplementary Information [file srep16724-s1.pdf]

Tracing the structural evolution of eukaryotic ATP Binding cassette transporter superfamily

Jie Xiong[1], Jinmei Feng[1], DongxiaYuan[1], Jun Zhou[2*], Wei Miao[1*]

[1]Key Laboratory of Aquatic Biodiversity and Conservation, Institute of Hydrobiology, Chinese Academy of Sciences, Wuhan, 430072, PR China

[2]Department of Organismic and Evolutionary Biology, Harvard University, MA 02138, USA

[*] Corresponding author:

Jun Zhou

Email: jzhou@oeb.harvard.edu

Tel: +1- 617-496-5540

Wei Miao

Email: miaowei@ihb.ac.cn

Tel: +86-27-68780050

Fax: +86-27-68780050

Running title: Gene structure evolution of ABC transporter

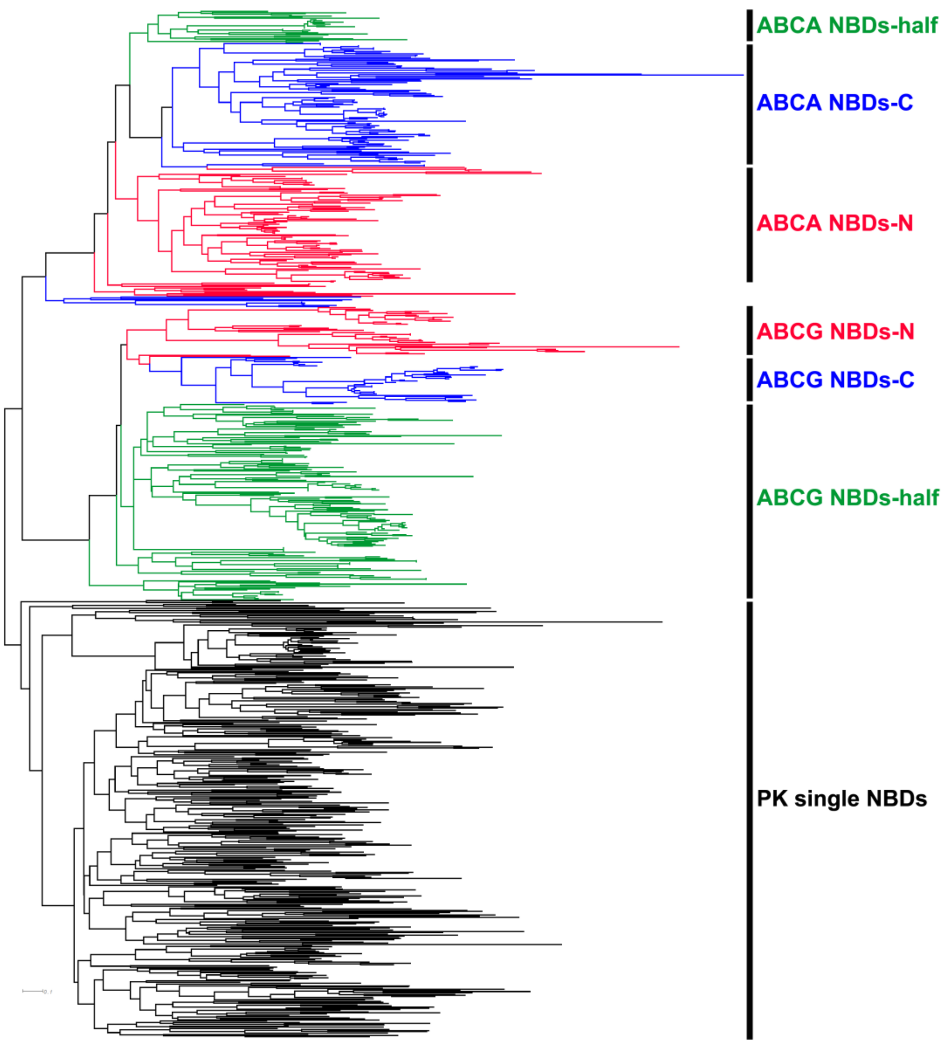

Figure S1. The phylogenetic relationship of ABCA and ABCG family NBDs. PK, prokaryotes; NBDs-half, NBDs from half structure transporters; NBDs-N, N-terminal NBDs from the ABC full or ABC2 structure transporters; NBDs-C, C-terminal NBDs from the ABC full or ABC2 structure transporters.

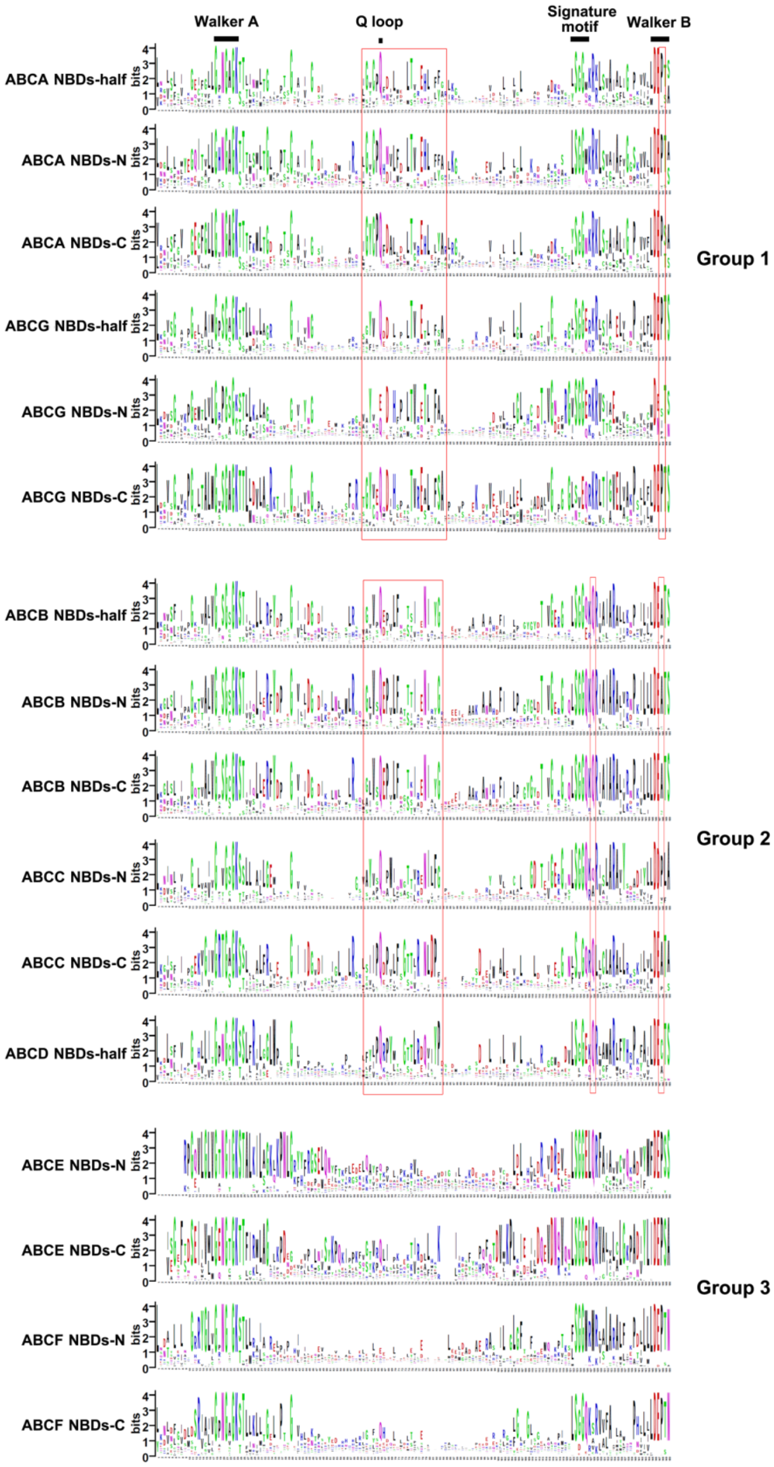

Figure S2. Sequences conservation of NBDs. The Walker A, Q-loop, signature motif and Walker B are conserved motifs of NBD.

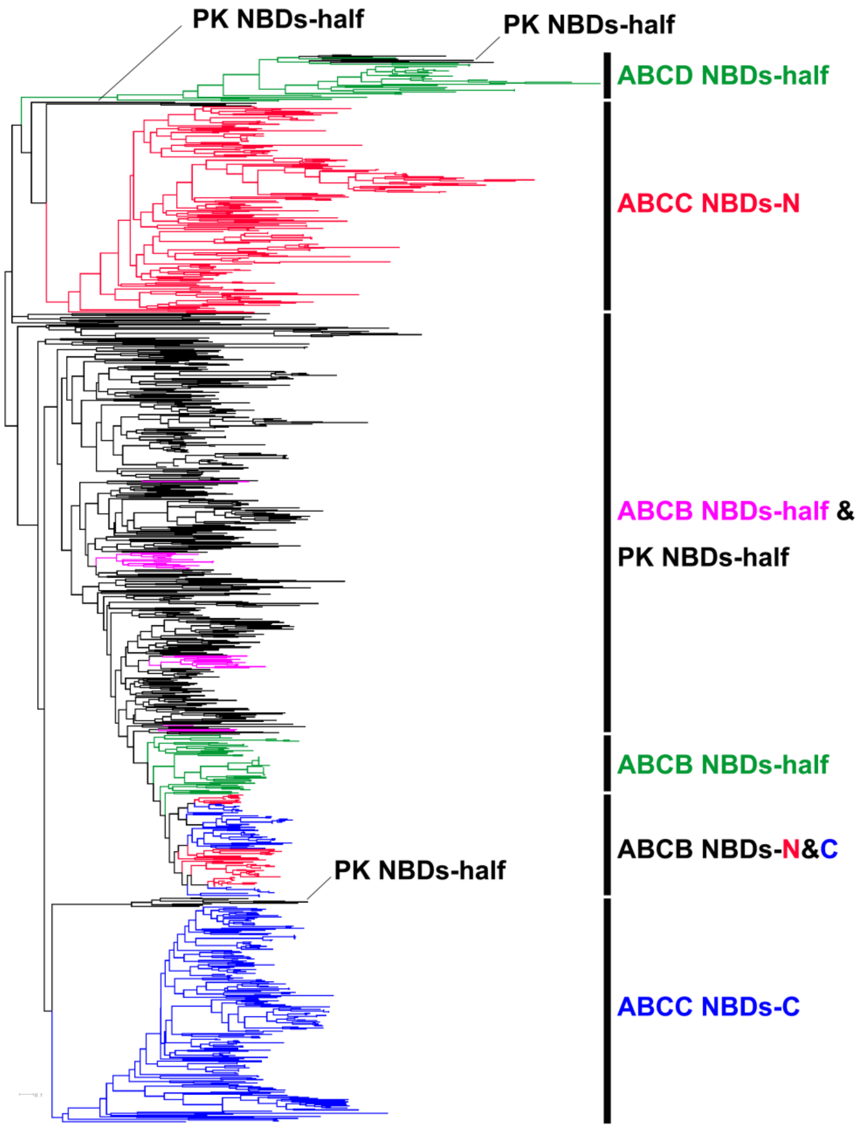

Figure S3. The phylogenetic relationship of ABCB, ABCC and ABCD family NBDs. PK, prokaryotes; NBDs-half, NBDs from half structure transporters; NBDs-N, N-terminal NBDs from the ABC full or ABC2 structure transporters; NBDs-C, C-terminal NBDs from the ABC full or ABC2 structure transporters.

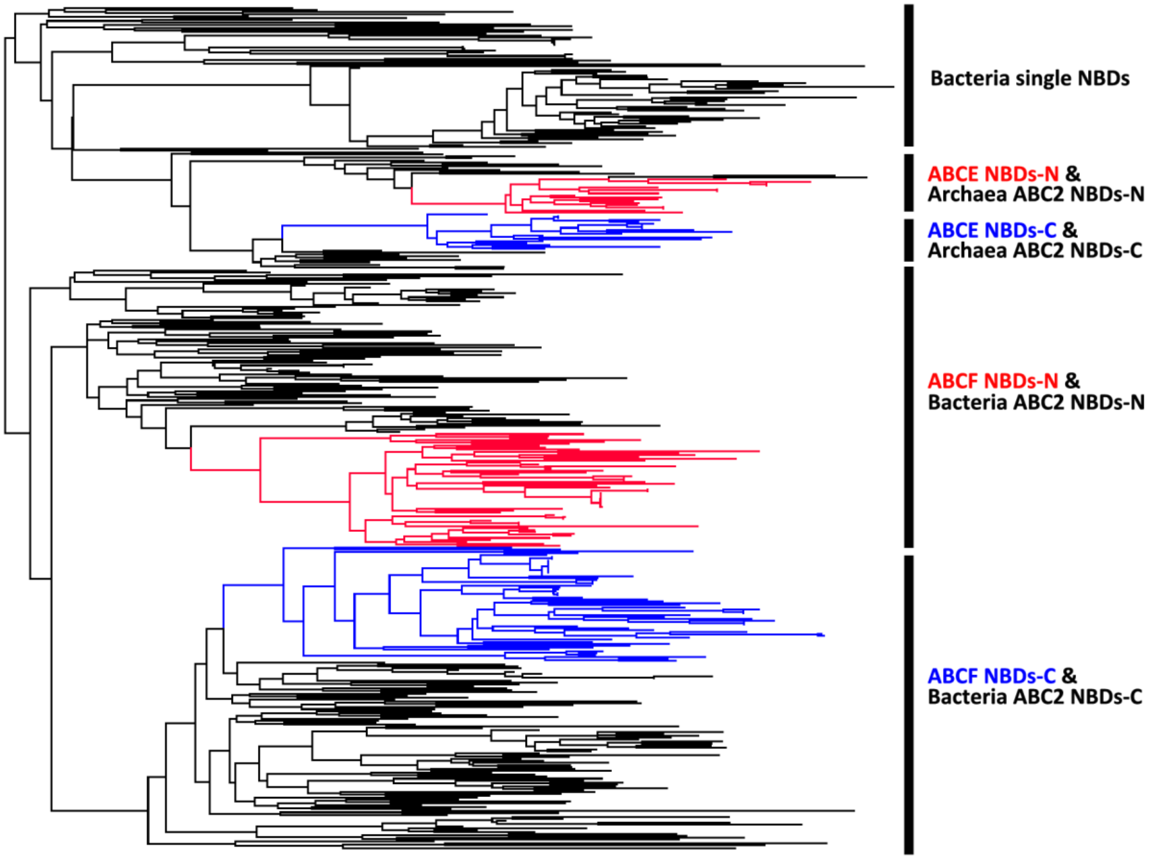

Figure S4. The phylogenetic relationship of ABCE and ABCF family NBDs. NBDs-N, N-terminal NBDs from the ABC2 structure transporters; NBDs-C, C-terminal NBDs from the ABC2 structure transporters.

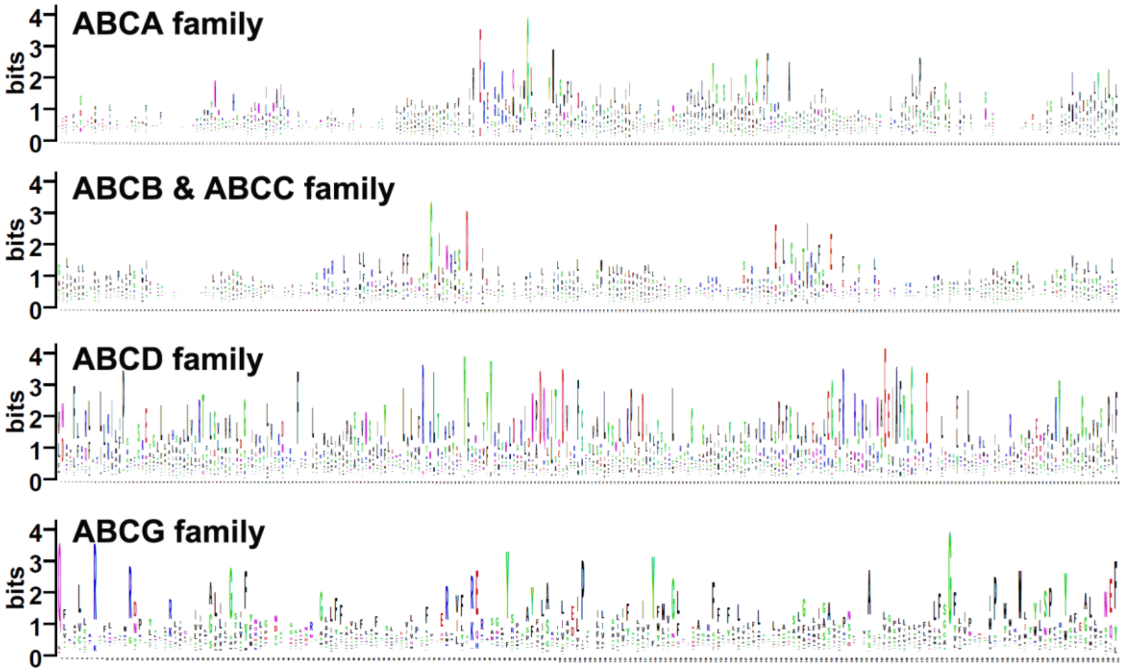

Figure S5. Sequence conservation of eukaryotes TMDs.

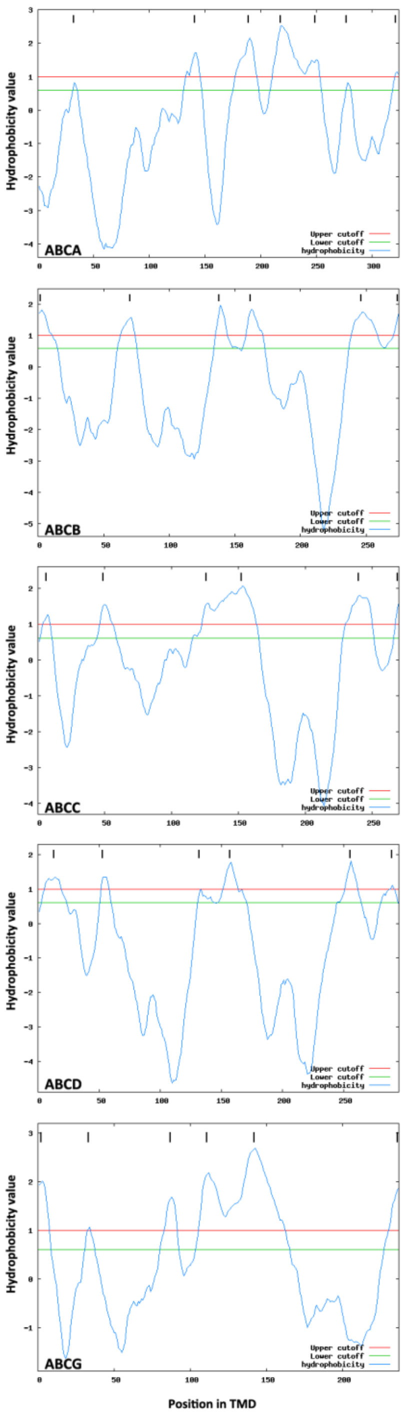

Figure S6. The examples of transmembrane helix patterns. The helices were predicted using the extracted TMDs by TopPred. The X-axis represents the amino acid position in TMDs, and the Y-axis represents the hydrophobicity value. Each black vertical bar represents a transmembrane helix. The human genes ENSP00000359245 (ABCA), ENSP00000265724 (ABCB), ENSP00000303960 (ABCC), ENSP00000349396 (ABCD) and ENSP00000237612 (ABCG) were used as examples.

Figure S7. The raw NBDs tree with local support values. B-PRO, bacteria; A-PRO, archeae; EU, eukaryotes. 1, NBDs-N; 2, NBDs-C.

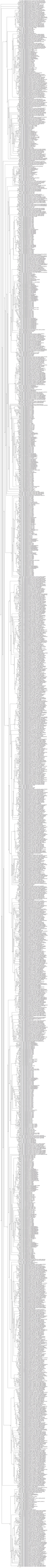

Table S1. The ABC transporter genes in 79 selected eukaryotes.

Species	Category	ABCA		ABCB		ABCC		ABCD		ABCE		ABCF		ABCG	
		half	full	half	full	half or single	full	half	full	single	ABC2	single	ABC2	half	full
Tetrahymena thermophila SB210	Alveolata	4	29	13	13	0	60	2	0	0	1	0	2	36	3
Plasmodium falciparum 3D7	Alveolata	1	0	6	1	0	2	0	0	0	1	0	1	1	0
Plasmodium yoelii yoelii str. 17XNL	Alveolata	0	0	6	1	0	1	0	0	0	1	0	1	1	0
Plasmodium knowlesi strain H	Alveolata	1	0	6	1	0	1	0	0	0	1	0	1	1	0
Plasmodium chabaudi chabaudi	Alveolata	1	1	6	1	0	1	0	0	0	1	0	1	1	0
Plasmodium vivax Sal-1	Alveolata	1	0	6	1	0	2	0	0	0	1	0	2	1	0
Neospora caninum	Alveolata	0	0	6	2	0	3	2	0	0	1	0	1	6	0
Plasmodium berghei str. ANKA	Alveolata	1	0	6	1	0	1	0	0	0	1	0	1	1	0
Babesia bovis T2Bo	Alveolata	0	0	4	0	0	4	0	0	0	1	0	1	0	0
Toxoplasma gondii	Alveolata	0	0	5	2	0	3	1	0	0	1	0	2	6	0
Theileria parva strain Muguga	Alveolata	0	0	3	0	0	8	0	0	0	1g	0	1	0	0
Theileria annulata strain Ankara	Alveolata	0	0	3	0	0	18	0	0	0	1	0	1	0	0
Cryptosporidium muris RN66	Alveolata	0	0	1	1	2c	12	1	0	0	1	0	1	2	0
Cryptosporidium parvum Iowa II	Alveolata	0	0	1	1	3c	11	1	0	0	1	0	1	2	0
Dictyostelium discoideum AX4	Amoebozoa	6	5	9	2	0	13	3	0	0	1	0	3	9	15
Entamoeba dispar SAW760	Amoebozoa	2	0	9	6	1a	8	0	0	1b	1	1b	2	1	0
Entamoeba histolytica HM-1:IMSS	Amoebozoa	2	0	7	6	2a	6	1	0	2f	1	0	2	1	0
Trypanosoma brucei gambiense	Euglenozoa	0	2	5	0	0	2	3	0	0	1	0	3	4	0
Leishmania braziliensis	Euglenozoa	0	9	6	1	0	7	3	0	0	1	0	3	6	0
Leishmania major strain Friedlin	Euglenozoa	0	10	5	2	0	8	3	0	0	1	0	3	7	0
Trypanosoma vivax	Euglenozoa	2	0	5	0	0	3	2	0	0	1	2f	3	3	0
Trypanosoma brucei	Euglenozoa	0	4	10	0	0	6	6	0	0	2	0	6	8	0
Leishmania mexicana	Euglenozoa	2	9	5	2	0	9	3	0	0	1	0	3	6	0
Leishmania infantum	Euglenozoa	2	9	5	2	0	9	3	0	0	1	0	3	7	0
Trypanosoma cruzi strain CL Brener	Euglenozoa	1	4	5	0	4f	1	4	0	0	3	0	2	8	0
Coccidioides posadasii RMSCC 3488	Fungi	0	1	6	6	0	6	2	0	0	1	0	4	2	7

Species	Category	ABCA		ABCB		ABCC		ABCD		ABCE		ABCF		ABCG	
		half	full	half	full	half or single	full	half	full	single	ABC2	single	ABC2	half	full
Scheffersomyces stipitis CBS 6054	Fungi	0	0	3	2	0	10	3	0	0	1g	0	4	2	7
Encephalitozoon intestinalis	Fungi	0	0	6	0	0	0	0	0	0	1	0	1	6	0
Cryptococcus neoformans var. grubii H99	Fungi	0	0	4	2	0	9	2	0	0	1	0	4	2	7
Neurospora crassa OR74A	Fungi	0	1	4	5	0	9	3	0	0	1	0	4	3	4
Eremothecium gossypii	Fungi	0	0	3	1	0	3	2	0	0	1	0	4	1	4
Candida glabrata CBS 138	Fungi	0	0	3	1	0	5	2	0	0	1	0	4	1	6
Kluyveromyces lactis NRRL Y-1140	Fungi	0	0	3	1	0	4	2	0	0	1	0	4	1	5
Coccidioides immitis RS	Fungi	0	1	10	6	0	6	2	0	0	1	0	6	2	7
Magnaporthe oryzae 70-15	Fungi	0	0	1	0	0	0	0	0	0	1g	0	1	0	1
Yarrowia lipolytica CLIB122	Fungi	0	1	2	4	0	10	2	0	0	1	0	4	0	6
Encephalitozoon cuniculi GB-M1	Fungi	0	0	6	0	0	0	0	0	0	1	0	1	5	0
Schizosaccharomyces pombe	Fungi	0	0	3	2	0	4	0	0	0	1	0	5	0	2
Gibberella zeae PH-1	Fungi	0	1	6	10	0	16	2	0	0	1	0	5	3	16
Aspergillus fumigatus Af293	Fungi	0	1	4	10	0	11	2	0	0	1	0	5	2	13
Debaryomyces hansenii CBS767	Fungi	0	0	3	2	1b	4	2	0	0	1	0	5	5	7
Laccaria bicolor S238N-H82	Fungi	0	2	13	5	1b	8	2	0	0	1g	0	4	4	5
Emericella nidulans	Fungi	0	2	3	8	1*	11	3	0	0	1	0	5	2	14
Saccharomyces cerevisiae S288c	Fungi	0	0	3	1	2d	5	2	0	0	1	0	5	1	9
Cryptococcus bacillisporus	Fungi	0	0	4	2	2c	7	2	0	0	1	0	4	2	6
Candida albicans	Fungi	0	0	6	2	2e	8	4	0	0	2	0	8	6	12
Pan troglodytes	Metazoa	0	12	6	5	0	12	4	0	0	1	0	3	5	0
Pediculus humanus	Metazoa	0	2	3	4	0	5	2	0	0	1	0	3	18	0
Caenorhabditis briggsae AF16	Metazoa	1	6	12	17	0	10	5	0	0	1	0	3	12	0
Takifugu rubripes	Metazoa	5	7	9	3	0	13	3	0	0	1	0	3	8	0
Homo sapiens	Metazoa	0	12	21	4	0	12	4	0	0	1	0	9	5	0
Mus musculus	Metazoa	0	15	8	4	0	11	4	0	0	1	0	4	6	0
Caenorhabditis elegans	Metazoa	2	4	10	14	0	9	5	0	0	1	0	3	10	0

Species	Category	ABCA		ABCB		ABCC		ABCD		ABCE		ABCF		ABCG	
		half	full	half	full	half or single	full	half	full	single	ABC2	single	ABC2	half	full
Equus caballus	Metazoa	0	14	7	3	0	14	3	0	0	1	0	2	5	0
Drosophila melanogaster	Metazoa	0	10	4	4	0	14	2	0	0	1	0	3	18	0
Trichoplax adhaerens	Metazoa	0	5	10	1	1c	23	3	0	0	1g	1b	3	1	0
Gallus gallus	Metazoa	1	9	5	4	1c	10	3	0	0	1	0	2	5	0
Apis mellifera	Metazoa	0	3	5	1	1a	8	2	0	0	1	0	3	18	0
Canis lupus familiaris	Metazoa	0	15	7	4	2f	12	4	0	0	1	0	3	5	0
Brugia malayi	Metazoa	0	2	9	3	2f	2	1	0	0	1	0	3	3	0
Culex pipiens	Metazoa	0	10	4	1	2f	14	2	0	0	1	0	3	29	0
Danio rerio	Metazoa	3	6	12	3	3*	13	3	0	0	1	0	4	10	0
Monodelphis domestica	Metazoa	3	13	8	4	3b*	9	4	0	0	1	0	3	5	0
Anopheles gambiae str. PEST	Metazoa	0	9	4	1	3ac	10	2	0	0	1	0	3	19	0
Tetraodon nigroviridis	Metazoa	2	8	10	3	3e	12	4	0	0	1	2be	2	8	0
Rattus norvegicus	Metazoa	0	13	7	4	4e	8	4	0	0	1	0	3	9	0
Macaca mulatta	Metazoa	1	15	18	3	4bf	13	4	0	0	1	0	3	5	0
Aedes aegypti	Metazoa	0	12	4	1	4abc	17	2	0	0	1	0	3	18	0
Giardia lamblia ATCC 50803	Fornicata	8	7	1	0	0	4	0	0	0	1	0	1	0	0
Giardia lamblia P15	Fornicata	7	7	1	0	0	4	0	0	0	1	0	1	1	0
Giardia intestinalis ATCC 50581	Fornicata	8	4	1	0	0	2	1	0	0	1	0	1	1	0
Monosiga brevicollis MX1	Choanoflagellida	3	3	6	1	4f	6	1	0	0	1	0	7	7	2
Thalassiosira pseudonana CCMP1335	Stramenopiles	3	0	10	3	2f	6	3	0	0	1	0	11	8	2
Cyanidioschyzon merolae strain 10D	Viridiplantae	2	0	8	0	0	2	3	0	0	1	0	2	8	0
Physcomitrella patens subsp. patens	Viridiplantae	7	2	11	10	0	14	4	2*	0	1g	0	8	27	15
Ostreococcus tauri	Viridiplantae	1	1	4	1	0	2	2	0	0	1	2b	11	10	1
Micromonas sp. RCC299	Viridiplantae	1	3	9	1	0	2	3	0	0	1	0	15	10	2
Arabidopsis thaliana	Viridiplantae	11	2	10	21	0	17	1	1*	1*	2	0	5	35	15
Oryza sativa Japonica Group	Viridiplantae	5	0	14	9	4cf	14	1	1*	0	3	0	5	38	6

a: Predicted gene located in the short assembled scaffold or the terminal of a scaffold.
b: Predicted gene flanked the genome assembly gap in upstream or downstream (or both).
c: Mis-predicted gene, two adjacent NBDs could be found by scanning the DNA sequence in these regions using six open reading frames.

d: This gene is a half structure in *Saccharomyces cerevisiae S288c*, but a full structure in other *Saccharomyces cerevisiae* strain.
e: Gene predictions without a complete open reading frame, only partial sequence of a real gene.
f: Adjacent genes.
g: Genes have not found in predicted proteome, but found in genome assembly, not included in the phylogenetic analysis.
*: No evidence suggests this gene is mis-predicted.
Note that alternative splicing variants also included if it contained in the whole genome protein annotation.

Table S2. List of the prokaryotes selected to investigate the ABC transporter gene structures.

Selected prokaryotic genomes
Acaryochloris marina MBIC11017 uid58167
Acetobacterium woodii DSM 1030 uid88073
Acetobacter pasteurianus IFO 3283 01 uid59279
Acetobacter pasteurianus IFO 3283 03 uid158373
Acetobacter pasteurianus IFO 3283 07 uid158381
Acetobacter pasteurianus IFO 3283 12 uid158379
Acetobacter pasteurianus IFO 3283 22 uid158383
Acetobacter pasteurianus IFO 3283 26 uid158531
Acetobacter pasteurianus IFO 3283 32 uid158375
Acetohalobium arabaticum DSM 5501 uid51423
Acholeplasma laidlawii PG 8A uid58901
Achromobacter xylosoxidans A8 uid59899
Acidaminococcus fermentans DSM 20731 uid43471
Acidaminococcus intestini RyC MR95 uid74445
Acidianus hospitalis W1 uid66875
Acidilobus saccharovorans 345 15 uid51395
Acidimicrobidae bacterium YM16 304 uid193703
Acidimicrobium ferrooxidans DSM 10331 uid59215
Acidiphilium cryptum JF 5 uid58447
Acidiphilium multivorum AIU301 uid63345
Acidithiobacillus caldus SM 1 uid70791
Acidithiobacillus ferrivorans SS3 uid67387
Acidobacterium capsulatum ATCC 51196 uid59127
Acidobacterium MP5ACTX9 uid50551
Acidothermus cellulolyticus 11B uid58501
Acidovorax avenae ATCC 19860 uid42497

**Selected prokaryotic genomes**
Acidovorax citrulli AAC00 1 uid58429
Acidovorax ebreus TPSY uid59233
Acidovorax JS42 uid58427
Acidovorax KKS102 uid176500
Aciduliprofundum boonei T469 uid43333
Aciduliprofundum MAR08 339 uid184407
Acinetobacter ADP1 uid61597
Acinetobacter baumannii 1656 2 uid158677
Acinetobacter baumannii AB0057 uid59083
Acinetobacter baumannii AB307 0294 uid59271
Acinetobacter baumannii ACICU uid58765
Acinetobacter baumannii ATCC 17978 uid58731
Acinetobacter baumannii AYE uid61637
Acinetobacter baumannii BJAB07104 uid210971
Acinetobacter baumannii BJAB0715 uid210972
Acinetobacter baumannii BJAB0868 uid210973
Acinetobacter baumannii D1279779 uid190222
Acinetobacter baumannii MDR TJ uid162739
Acinetobacter baumannii MDR ZJ06 uid158685
Acinetobacter baumannii SDF uid61601
Acinetobacter baumannii TCDC AB0715 uid158679
Acinetobacter baumannii TYTH 1 uid176498
Acinetobacter calcoaceticus PHEA 2 uid83123
Acinetobacter oleivorans DR1 uid50119
Actinobacillus succinogenes 130Z uid58247
Actinobacillus suis H91 0380 uid176363
Actinoplanes missouriensis 431 uid158169

Selected prokaryotic genomes
Actinoplanes N902 109 uid202219
Actinoplanes SE50 110 uid162333
Actinosynnema mirum DSM 43827 uid58951
Advenella kashmirensis WT001 uid80859
Aequorivita sublithincola DSM 14238 uid168181
Aerococcus urinae ACS 120 V Col10a uid64757
Aeromonas hydrophila ATCC 7966 uid58617
Aeromonas hydrophila ML09 119 uid205540
Aeromonas salmonicida A449 uid58631
Aeromonas veronii B565 uid66323
Aeropyrum pernix K1 uid57757
Aggregatibacter aphrophilus NJ8700 uid59407
Agrobacterium fabrum C58 uid57865
Agrobacterium H13 3 uid63403
Agrobacterium radiobacter K84 uid58269
Agrobacterium vitis S4 uid58249
Agromonas oligotrophica S58 uid192186
Akkermansia muciniphila ATCC BAA 835 uid58985
Alcanivorax borkumensis SK2 uid58169
Alcanivorax dieselolei B5 uid176364
Alicycliphilus denitrificans BC uid49953
Alicycliphilus denitrificans K601 uid66307
Aliivibrio salmonicida LFI1238 uid59251
Alistipes finegoldii DSM 17242 uid168180
Alistipes shahii WAL 8301 uid197175
Alkalilimnicola ehrlichii MLHE 1 uid58467
Alkaliphilus metalliredigens QYMF uid58171

Selected prokaryotic genomes
Alkaliphilus oremlandii OhILAs uid58495
Allochromatium vinosum DSM 180 uid46083
alpha proteobacterium HIMB59 uid175778
alpha proteobacterium HIMB5 uid175779
Alteromonas macleodii AltDE1 uid179068
Alteromonas macleodii ATCC 27126 uid55253
Alteromonas macleodii Black Sea 11 uid176365
Alteromonas macleodii Deep ecotype uid58251
Alteromonas macleodii Ionian Sea U4 uid210780
Alteromonas macleodii Ionian Sea U7 uid210785
Alteromonas macleodii Ionian Sea U8 uid210782
Alteromonas SN2 uid67349
Aminobacterium colombiense DSM 12261 uid47083
Ammonifex degensii KC4 uid41053
Amphibacillus xylanus NBRC 15112 uid176453
Amycolatopsis mediterranei S699 uid158689
Amycolatopsis mediterranei S699 uid171830
Amycolatopsis mediterranei U32 uid50565
Amycolatopsis orientalis HCCB10007 uid203791
Amycolicicoccus subflavus DQS3 9A1 uid67253
Anabaena 90 uid179383
Anabaena cylindrica PCC 7122 uid183339
Anabaena variabilis ATCC 29413 uid58043
Anaerobaculum mobile DSM 13181 uid168323
Anaerococcus prevotii DSM 20548 uid59219
Anaerolinea thermophila UNI 1 uid62245
Anaeromyxobacter dehalogenans 2CP 1 uid58989

Selected prokaryotic genomes
Anaeromyxobacter dehalogenans 2CP C uid58135
Anaeromyxobacter Fw109 5 uid58755
Anaeromyxobacter K uid58953
Anaplasma centrale Israel uid42155
Anaplasma marginale Florida uid58577
Anaplasma marginale Maries uid57629
Anaplasma phagocytophilum Dog2 uid213225
Anaplasma phagocytophilum HZ2 uid213224
Anaplasma phagocytophilum HZ uid57951
Anaplasma phagocytophilum JM uid213223
Anoxybacillus flavithermus WK1 uid59135
Aquifex aeolicus VF5 uid57765
Arcanobacterium haemolyticum DSM 20595 uid49489
Archaeoglobus fulgidus DSM 4304 uid57717
Archaeoglobus profundus DSM 5631 uid43493
Archaeoglobus sulfaticallidus PM70 1 uid201033
Archaeoglobus veneficus SNP6 uid65269
archaeon Mx1201 uid196597
Arcobacter butzleri 7h1h uid200766
Arcobacter butzleri ED 1 uid158699
Arcobacter butzleri RM4018 uid58557
Arcobacter L uid158135
Arcobacter nitrofigilis DSM 7299 uid49001
Aromatoleum aromaticum EbN1 uid58231
Arthrobacter arilaitensis Re117 uid53509
Arthrobacter aurescens TC1 uid58109
Arthrobacter chlorophenolicus A6 uid58969

Selected prokaryotic genomes
Arthrobacter FB24 uid58141
Arthrobacter nitroguajacolicus Rue61a uid174511
Arthrobacter phenanthrenivorans Sphe3 uid63629
Arthrospira platensis NIES 39 uid197171
Asticcacaulis excentricus CB 48 uid55641
Atopobium parvulum DSM 20469 uid59195
Azoarcus BH72 uid61603
Azoarcus KH32C uid193704
Azorhizobium caulinodans ORS 571 uid58905
Azospirillum B510 uid46085
Azospirillum brasilense Sp245 uid162161
Azospirillum lipoferum 4B uid82343
Azotobacter vinelandii CA6 uid198830
Azotobacter vinelandii CA uid198829
Azotobacter vinelandii DJ uid57597
Bacillus 1NLA3E uid81841
Bacillus amyloliquefaciens DSM 7 uid53535
Bacillus amyloliquefaciens FZB42 uid58271
Bacillus amyloliquefaciens IT 45 uid181617
Bacillus amyloliquefaciens LL3 uid158133
Bacillus amyloliquefaciens TA208 uid158701
Bacillus amyloliquefaciens XH7 uid158881
Bacillus amyloliquefaciens Y2 uid165195
Bacillus anthracis A0248 uid59385
Bacillus anthracis Ames Ancestor uid58083
Bacillus anthracis Ames uid57909
Bacillus anthracis CDC 684 uid59303

Selected prokaryotic genomes
Bacillus anthracis H9401 uid162021
Bacillus anthracis Sterne uid58091
Bacillus atrophaeus 1942 uid59887
Bacillus cellulosilyticus DSM 2522 uid43329
Bacillus cereus 03BB102 uid59299
Bacillus cereus AH187 uid58753
Bacillus cereus AH820 uid58751
Bacillus cereus ATCC 10987 uid57673
Bacillus cereus ATCC 14579 uid57975
Bacillus cereus B4264 uid58757
Bacillus cereus biovar anthracis CI uid50615
Bacillus cereus E33L uid58103
Bacillus cereus F837 76 uid83611
Bacillus cereus FRI 35 uid173403
Bacillus cereus G9842 uid58759
Bacillus cereus NC7401 uid82815
Bacillus cereus Q1 uid58529
Bacillus clausii KSM K16 uid58237
Bacillus coagulans 2 6 uid68053
Bacillus coagulans 36D1 uid54335
Bacillus cytotoxicus NVH 391 98 uid58317
Bacillus halodurans C 125 uid57791
Bacillus JS uid162189
Bacillus licheniformis 9945A uid207072
Bacillus licheniformis ATCC 14580 uid58097
Bacillus megaterium DSM319 uid48371
Bacillus megaterium QM B1551 uid15862

Selected prokaryotic genomes
Bacillus megaterium WSH 002 uid159841
Bacillus pseudofirmus OF4 uid45847
Bacillus pumilus SAFR 032 uid59017
Bacillus selenitireducens MLS10 uid49513
Bacillus subtilis 168 uid57675
Bacillus subtilis 6051 HGW uid193706
Bacillus subtilis BAB 1 uid195461
Bacillus subtilis BSn5 uid62463
Bacillus subtilis BSP1 uid184010
Bacillus subtilis natto BEST195 uid183001
Bacillus subtilis QB928 uid173926
Bacillus subtilis RO NN 1 uid158879
Bacillus subtilis spizizenii TU B 10 uid73967
Bacillus subtilis spizizenii W23 uid51879
Bacillus subtilis XF 1 uid189187
Bacillus thuringiensis Al Hakam uid58795
Bacillus thuringiensis BMB171 uid49135
Bacillus thuringiensis Bt407 uid177931
Bacillus thuringiensis HD 771 uid173374
Bacillus thuringiensis HD 789 uid173860
Bacillus thuringiensis MC28 uid176369
Bacillus thuringiensis serovar IS5056 uid190186
Bacillus weihenstephanensis KBAB4 uid58315
Bacteriovorax marinus SJ uid82341
bacterium BT 1 uid184079
Bacteroides fragilis 638R uid84217
Bacteroides fragilis NCTC 9343 uid57639

Selected prokaryotic genomes
Bacteroides fragilis YCH46 uid58195
Bacteroides helcogenes P 36 108 uid62135
Bacteroides salanitronis DSM 18170 uid63269
Bacteroides thetaiotaomicron VPI 5482 uid62913
Bacteroides vulgatus ATCC 8482 uid58253
Bacteroides xylanisolvens XB1A uid197168
Bartonella australis Aust NH1 uid189950
Bartonella bacilliformis KC583 uid58533
Bartonella clarridgeiae 73 uid62131
Bartonella grahamii as4aup uid59405
Bartonella henselae Houston 1 uid57745
Bartonella quintana RM 11 uid174512
Bartonella quintana Toulouse uid57635
Bartonella tribocorum CIP 105476 uid59129
Bartonella vinsonii berkhoffii Winnie uid189951
Bdellovibrio bacteriovorus HD100 uid61595
Bdellovibrio bacteriovorus Tiberius uid182482
Bdellovibrio exovorus JSS uid194119
Beijerinckia indica ATCC 9039 uid59057
Belliella baltica DSM 15883 uid168182
beta proteobacterium CB uid191340
Beutenbergia cavernae DSM 12333 uid59047
Bibersteinia trehalosi 192 uid193709
Bifidobacterium animalis ATCC 25527 uid162513
Bifidobacterium animalis lactis AD011 uid58911
Bifidobacterium animalis lactis B420 uid163691
Bifidobacterium animalis lactis BB 12 uid158871

**Selected prokaryotic genomes**
Bifidobacterium animalis lactis Bi 07 uid163693
Bifidobacterium animalis lactis Bl 04 uid59359
Bifidobacterium animalis lactis Bl12 uid210081
Bifidobacterium animalis lactis BLC1 uid158867
Bifidobacterium animalis lactis V9 uid158865
Bifidobacterium asteroides PRL2011 uid176921
Bifidobacterium bifidum BGN4 uid167988
Bifidobacterium bifidum PRL2010 uid59883
Bifidobacterium bifidum S17 uid59545
Bifidobacterium breve ACS 071 V Sch8b uid158863
Bifidobacterium breve UCC2003 uid193702
Bifidobacterium dentium Bd1 uid43091
Bifidobacterium longum BBMN68 uid60163
Bifidobacterium longum DJO10A uid58833
Bifidobacterium longum F8 uid197184
Bifidobacterium longum infantis 157F uid62693
Bifidobacterium longum JCM 1217 uid62695
Bifidobacterium longum JDM301 uid49131
Bifidobacterium longum KACC 91563 uid158861
Bifidobacterium longum NCC2705 uid57939
Bifidobacterium thermophilum RBL67 uid193770
Blastococcus saxobsidens DD2 uid89391
Blattabacterium Blaberus giganteus uid165873
Bordetella avium 197N uid61563
Bordetella bronchiseptica 253 uid178913
Bordetella bronchiseptica MO149 uid177517
Bordetella bronchiseptica RB50 uid57613

Selected prokaryotic genomes
Bordetella parapertussis 12822 uid57615
Bordetella parapertussis 18323 uid175569
Bordetella parapertussis Bpp5 uid177516
Bordetella pertussis CS uid158859
Bordetella pertussis Tohama I uid57617
Bordetella petrii uid61631
Borrelia afzelii HLJ01 uid177930
Borrelia afzelii PKo uid159867
Borrelia afzelii PKo uid58653
Borrelia bissettii DN127 uid71231
Borrelia burgdorferi B31 uid57581
Borrelia burgdorferi JD1 uid161197
Borrelia burgdorferi N40 uid161241
Borrelia burgdorferi ZS7 uid59429
Borrelia crocidurae Achema uid162335
Borrelia duttonii Ly uid58791
Borrelia garinii BgVir uid162165
Borrelia garinii NMJW1 uid177081
Borrelia garinii PBi uid58125
Borrelia hermsii DAH uid59225
Borrelia recurrentis A1 uid58793
Borrelia turicatae 91E135 uid58311
Borrelia valaisiana VS116 uid54823
Brachybacterium faecium DSM 4810 uid58649
Brachyspira hyodysenteriae WA1 uid59291
Brachyspira intermedia PWS A uid158369
Brachyspira murdochii DSM 12563 uid48819

Selected prokaryotic genomes
Brachyspira pilosicoli 95 1000 uid50609
Brachyspira pilosicoli B2904 uid175255
Brachyspira pilosicoli P43 6 78 uid184077
Brachyspira pilosicoli WesB uid175256
Bradyrhizobium BTAi1 uid58505
Bradyrhizobium japonicum USDA 110 uid57599
Bradyrhizobium japonicum USDA 6 uid158851
Bradyrhizobium ORS 278 uid58941
Bradyrhizobium S23321 uid158167
Brevibacillus brevis NBRC 100599 uid59175
Brevundimonas subvibrioides ATCC 15264 uid42117
Brucella abortus A13334 uid83615
Brucella abortus bv 1 9 941 uid58019
Brucella abortus S19 uid58873
Brucella canis ATCC 23365 uid59009
Brucella canis HSK A52141 uid83613
Brucella melitensis ATCC 23457 uid59241
Brucella melitensis bv 1 16M uid57735
Brucella melitensis M28 uid158857
Brucella melitensis M5 90 uid158855
Brucella melitensis NI uid158853
Brucella microti CCM 4915 uid59319
Brucella ovis ATCC 25840 uid58113
Brucella pinnipedialis B2 94 uid71131
Brucella suis 1330 uid159871
Brucella suis 1330 uid57927
Brucella suis ATCC 23445 uid59015

**Selected prokaryotic genomes**
Brucella suis VBI22 uid83617
Buchnera aphidicola Cc Cinara cedri uid58579
Burkholderia 383 uid58073
Burkholderia ambifaria AMMD uid58303
Burkholderia ambifaria MC40 6 uid58701
Burkholderia CCGE1001 uid42975
Burkholderia CCGE1002 uid42523
Burkholderia CCGE1003 uid46253
Burkholderia cenocepacia AU 1054 uid58371
Burkholderia cenocepacia HI2424 uid58369
Burkholderia cenocepacia J2315 uid57953
Burkholderia cenocepacia MC0 3 uid58769
Burkholderia cepacia GG4 uid173858
Burkholderia gladioli BSR3 uid66301
Burkholderia glumae BGR1 uid59397
Burkholderia KJ006 uid165871
Burkholderia mallei ATCC 23344 uid57725
Burkholderia mallei NCTC 10229 uid58383
Burkholderia mallei NCTC 10247 uid58385
Burkholderia mallei SAVP1 uid58387
Burkholderia multivorans ATCC 17616 uid58697
Burkholderia multivorans ATCC 17616 uid58909
Burkholderia phenoliruptrix BR3459a uid176370
Burkholderia phymatum STM815 uid58699
Burkholderia phytofirmans PsJN uid58729
Burkholderia pseudomallei 1026b uid162511
Burkholderia pseudomallei 1106a uid58515

Selected prokaryotic genomes
Burkholderia pseudomallei 1710b uid58391
Burkholderia pseudomallei 668 uid58389
Burkholderia pseudomallei BPC006 uid174460
Burkholderia pseudomallei K96243 uid57733
Burkholderia pseudomallei MSHR305 uid213227
Burkholderia pseudomallei MSHR346 uid55259
Burkholderia rhizoxinica HKI 454 uid60487
Burkholderia RPE64 uid205541
Burkholderia thailandensis E264 uid58081
Burkholderia thailandensis MSMB121 uid201037
Burkholderia vietnamiensis G4 uid58075
Burkholderia xenovorans LB400 uid57823
Burkholderia YI23 uid81081
butyrate producing bacterium SM4 1 uid197180
butyrate producing bacterium SS3 4 uid197159
butyrate producing bacterium SSC 2 uid197181
Butyrivibrio fibrisolvens uid197155
Butyrivibrio proteoclasticus B316 uid51489
Caldicellulosiruptor bescii DSM 6725 uid59201
Caldicellulosiruptor lactoaceticus 6A uid60575
Caldicellulosiruptor obsidiansis OB47 uid51501
Caldicellulosiruptor owensensis OL uid60165
Caldisericum exile AZM16c01 uid158173
Caldisphaera lagunensis DSM 15908 uid183486
Caldivirga maquilingensis IC 167 uid58711
Calothrix PCC 6303 uid183109
Calothrix PCC 7507 uid182930

Selected prokaryotic genomes
Campylobacter concisus 13826 uid58667
Campylobacter curvus 525 92 uid58669
Campylobacter fetus 82 40 uid58545
Campylobacter hominis ATCC BAA 381 uid58981
Campylobacter jejuni 81116 uid58771
Campylobacter jejuni 81 176 uid58503
Campylobacter jejuni doylei 269 97 uid58671
Campylobacter jejuni IA3902 uid159531
Campylobacter jejuni ICDCCJ07001 uid61249
Campylobacter jejuni M1 uid159535
Campylobacter jejuni NCTC 11168 BN148 uid174152
Campylobacter jejuni PT14 uid176499
Campylobacter jejuni RM1221 uid57899
Campylobacter jejuni S3 uid159533
Campylobacter lari RM2100 uid58115
Candidatus Amoebophilus asiaticus 5a2 uid58963
Candidatus Arthromitus SFB mouse Japan uid71379
Candidatus Arthromitus SFB mouse Yit uid159517
Candidatus Arthromitus SFB rat Yit uid73425
Candidatus Blochmannia floridanus uid57999
Candidatus Blochmannia vafer BVAF uid62083
Candidatus Carsonella ruddii DC uid213383
Candidatus Carsonella ruddii uid58773
Candidatus Hodgkinia cicadicola Dsem uid59311
Candidatus Methylomirabilis oxyfera uid161981
Candidatus Moranella endobia PCIT uid68739
Candidatus Moranella endobia PCVAL uid197215

**Selected prokaryotic genomes**
Candidatus Nitrosopumilus AR2 uid176130
Candidatus Nitrospira defluvii uid51175
Candidatus Pelagibacter IMCC9063 uid66305
Candidatus Phytoplasma australiense uid61641
Candidatus Phytoplasma mali uid59087
Candidatus Portiera aleyrodidarum TV uid195460
Candidatus Riesia pediculicola USDA uid46841
Candidatus Sulcia muelleri CARI uid52535
Candidatus Sulcia muelleri DMIN uid47075
Candidatus Sulcia muelleri GWSS uid58943
Candidatus Sulcia muelleri SMDSEM uid59393
Candidatus Uzinura diaspidicola ASNER uid186740
Candidatus Vesicomyosocius okutanii HA uid59427
Capnocytophaga canimorsus Cc5 uid70727
Capnocytophaga ochracea DSM 7271 uid59197
Carnobacterium 17 4 uid65789
Carnobacterium maltaromaticum LMA28 uid179370
Catenulispora acidiphila DSM 44928 uid59077
Caulobacter crescentus CB15 uid57891
Caulobacter crescentus NA1000 uid59307
Caulobacter K31 uid58551
Caulobacter segnis ATCC 21756 uid41709
Cellulomonas fimi ATCC 484 uid66779
Cellulomonas flavigena DSM 20109 uid48821
Cellulophaga algicola DSM 14237 uid62159
Cellulophaga lytica DSM 7489 uid63401
Cellvibrio gilvus ATCC 13127 uid68143

Selected prokaryotic genomes
Cellvibrio japonicus Ueda107 uid59139
Cenarchaeum symbiosum A uid61411
Chamaesiphon PCC 6605 uid183005
Chelativorans BNC1 uid58069
Chitinophaga pinensis DSM 2588 uid59113
Chlamydia muridarum Nigg uid57785
Chlamydia psittaci 01DC12 uid179070
Chlamydia psittaci 84 55 uid175571
Chlamydia psittaci GR9 uid175572
Chlamydia psittaci M56 uid175576
Chlamydia psittaci MN uid175573
Chlamydia psittaci VS225 uid175574
Chlamydia psittaci WC uid175577
Chlamydia psittaci WS RT E30 uid175575
Chlamydia trachomatis 434 Bu uid61633
Chlamydia trachomatis A2497 uid159863
Chlamydia trachomatis A2497 uid159993
Chlamydia trachomatis A 363 uid196769
Chlamydia trachomatis A 5291 uid196770
Chlamydia trachomatis A HAR 13 uid58333
Chlamydia trachomatis B Jali20 OT uid59351
Chlamydia trachomatis B TZ1A828 OT uid59349
Chlamydia trachomatis D EC uid159881
Chlamydia trachomatis D LC uid159879
Chlamydia trachomatis D SotonD1 uid196772
Chlamydia trachomatis D SotonD5 uid196773
Chlamydia trachomatis D SotonD6 uid196774

Selected prokaryotic genomes
Chlamydia trachomatis D UW 3 CX uid57637
Chlamydia trachomatis E 11023 uid161369
Chlamydia trachomatis E 150 uid161403
Chlamydia trachomatis E Bour uid196775
Chlamydia trachomatis E SotonE8 uid196777
Chlamydia trachomatis E SW3 uid167483
Chlamydia trachomatis F SW4 uid167484
Chlamydia trachomatis F SW5 uid167485
Chlamydia trachomatis G 11074 uid161409
Chlamydia trachomatis G 11222 uid161361
Chlamydia trachomatis G 9301 uid161377
Chlamydia trachomatis G 9768 uid161353
Chlamydia trachomatis G SotonG1 uid196779
Chlamydia trachomatis Ia SotonIa1 uid196780
Chlamydia trachomatis IU824 uid193712
Chlamydia trachomatis IU888 uid193713
Chlamydia trachomatis J 6276tet1 uid213394
Chlamydia trachomatis K SotonK1 uid196782
Chlamydia trachomatis L1 115 uid196784
Chlamydia trachomatis L1 224 uid196785
Chlamydia trachomatis L1 440 LN uid196783
Chlamydia trachomatis L2 25667R uid196786
Chlamydia trachomatis L2 434 Bu f uid198644
Chlamydia trachomatis L2 434 Bu i uid198643
Chlamydia trachomatis L2b 795 uid196791
Chlamydia trachomatis L2b 8200 07 uid196787
Chlamydia trachomatis L2b Ams1 uid196792

Selected prokaryotic genomes
Chlamydia trachomatis L2b Ams2 uid196793
Chlamydia trachomatis L2b Ams3 uid196794
Chlamydia trachomatis L2b Ams4 uid196795
Chlamydia trachomatis L2b Ams5 uid196796
Chlamydia trachomatis L2b Canada1 uid196798
Chlamydia trachomatis L2b Canada2 uid196799
Chlamydia trachomatis L2b CV204 uid196790
Chlamydia trachomatis L2b UCH 2 uid196788
Chlamydia trachomatis L2c uid68843
Chlamydia trachomatis L3 404 LN uid196797
Chlamydia trachomatis RC F 69 uid213385
Chlamydia trachomatis RC F s 342 uid213391
Chlamydia trachomatis RC F s 852 uid213387
Chlamydia trachomatis RC J 943 uid213388
Chlamydia trachomatis RC J 953 uid213389
Chlamydia trachomatis RC J 966 uid213393
Chlamydia trachomatis RC J 971 uid213395
Chlamydia trachomatis RC J s 122 uid213392
Chlamydia trachomatis RC L2 55 uid213396
Chlamydia trachomatis RC L2 s 3 uid213390
Chlamydia trachomatis RC L2 s 46 uid213386
Chlamydia trachomatis Sweden2 uid161995
Chlamydia trachomatis uid196771
Chlamydia trachomatis uid196776
Chlamydia trachomatis uid196778
Chlamydia trachomatis uid196781
Chlamydia trachomatis uid196789

**Selected prokaryotic genomes**
Chlamydia trachomatis uid196800
Chlamydophila abortus S26 3 uid57963
Chlamydophila caviae GPIC uid57783
Chlamydophila felis Fe C 56 uid57971
Chlamydophila pecorum E58 uid66295
Chlamydophila pneumoniae AR39 uid57809
Chlamydophila pneumoniae CWL029 uid57811
Chlamydophila pneumoniae J138 uid57829
Chlamydophila pneumoniae LPCoLN uid159529
Chlamydophila pneumoniae TW 183 uid57997
Chlamydophila psittaci 01DC11 uid159527
Chlamydophila psittaci 02DC15 uid159521
Chlamydophila psittaci 08DC60 uid159525
Chlamydophila psittaci 6BC uid159845
Chlamydophila psittaci 6BC uid63621
Chlamydophila psittaci C19 98 uid159523
Chlamydophila psittaci CP3 uid175578
Chlamydophila psittaci Mat116 uid189026
Chlamydophila psittaci NJ1 uid175579
Chlamydophila psittaci RD1 uid162063
Chlorobaculum parvum NCIB 8327 uid59185
Chlorobium chlorochromatii CaD3 uid58375
Chlorobium limicola DSM 245 uid58127
Chlorobium luteolum DSM 273 uid58175
Chlorobium phaeobacteroides BS1 uid58131
Chlorobium phaeobacteroides DSM 266 uid58133
Chlorobium phaeovibrioides DSM 265 uid58129

Selected prokaryotic genomes
Chlorobium tepidum TLS uid57897
Chloroflexus aggregans DSM 9485 uid58621
Chloroflexus aurantiacus J 10 fl uid57657
Chloroflexus Y 400 fl uid59085
Chloroherpeton thalassium ATCC 35110 uid59187
Chromobacterium violaceum ATCC 12472 uid58001
Chromohalobacter salexigens DSM 3043 uid62921
Chroococcidiopsis thermalis PCC 7203 uid183002
Chthonomonas calidirosea T49 uid208678
Citrobacter koseri ATCC BAA 895 uid58143
Citrobacter rodentium ICC168 uid43089
Clavibacter michiganensis NCPPB 382 uid61625
Clavibacter michiganensis sepedonicus uid61577
Clostridiales genomosp BVAB3 UPII9 5 uid46219
Clostridium acetobutylicum ATCC 824 uid57677
Clostridium acetobutylicum DSM 1731 uid68293
Clostridium acetobutylicum EA 2018 uid159515
Clostridium acidurici 9a uid176126
Clostridium beijerinckii NCIMB 8052 uid58137
Clostridium BNL1100 uid84307
Clostridium botulinum A2 Kyoto uid59229
Clostridium botulinum A3 Loch Maree uid59149
Clostridium botulinum A ATCC 19397 uid58927
Clostridium botulinum A ATCC 3502 uid61579
Clostridium botulinum A Hall uid58931
Clostridium botulinum B1 Okra uid59147
Clostridium botulinum Ba4 657 uid59173

**Selected prokaryotic genomes**
Clostridium botulinum B Eklund 17B uid59159
Clostridium botulinum BKT015925 uid66203
Clostridium botulinum E3 Alaska E43 uid59157
Clostridium botulinum F 230613 uid159513
Clostridium botulinum F Langeland uid58929
Clostridium botulinum H04402 065 uid162091
Clostridium cellulolyticum H10 uid58709
Clostridium cellulovorans 743B uid51503
Clostridium cf saccharolyticum K10 uid197201
Clostridium clariflavum DSM 19732 uid82345
Clostridium difficile 630 uid57679
Clostridium difficile BI1 uid158363
Clostridium difficile CD196 uid41017
Clostridium difficile R20291 uid40921
Clostridium kluyveri DSM 555 uid58885
Clostridium kluyveri NBRC 12016 uid59369
Clostridium lentocellum DSM 5427 uid49117
Clostridium ljungdahlii DSM 13528 uid50583
Clostridium novyi NT uid58643
Clostridium pasteurianum BC1 uid201478
Clostridium perfringens 13 uid57681
Clostridium perfringens ATCC 13124 uid57901
Clostridium perfringens SM101 uid58117
Clostridium phytofermentans ISDg uid58519
Clostridium saccharolyticum WM1 uid51419
Clostridium stercorarium DSM 8532 uid186819
Clostridium stercorarium DSM 8532 uid195569

Selected prokaryotic genomes
Clostridium sticklandii uid59585
Clostridium SY8519 uid68705
Clostridium tetani E88 uid57683
Clostridium thermocellum ATCC 27405 uid57917
Clostridium thermocellum DSM 1313 uid161989
Collimonas fungivorans Ter331 uid70793
Colwellia psychrerythraea 34H uid57855
Comamonas testosteroni CNB 2 uid62961
Conexibacter woesei DSM 14684 uid43467
Coprococcus ART55 1 uid197176
Coprococcus catus GD 7 uid197174
Coraliomargarita akajimensis DSM 45221 uid47079
Corallococcus coralloides DSM 2259 uid157997
Coriobacterium glomerans PW2 uid65787
Corynebacterium callunae DSM 20147 uid193714
Corynebacterium diphtheriae 241 uid83607
Corynebacterium diphtheriae 31A uid84309
Corynebacterium diphtheriae BH8 uid84311
Corynebacterium diphtheriae C7 beta uid84313
Corynebacterium diphtheriae CDCE 8392 uid84295
Corynebacterium diphtheriae HC01 uid84297
Corynebacterium diphtheriae HC02 uid84317
Corynebacterium diphtheriae HC03 uid84299
Corynebacterium diphtheriae HC04 uid84301
Corynebacterium diphtheriae INCA 402 uid83605
Corynebacterium diphtheriae NCTC 13129 uid57691
Corynebacterium diphtheriae PW8 uid84303

Selected prokaryotic genomes
Corynebacterium diphtheriae VA01 uid84305
Corynebacterium efficiens YS 314 uid62905
Corynebacterium glutamicum ATCC 13032 uid193708
Corynebacterium glutamicum ATCC 13032 uid57905
Corynebacterium glutamicum ATCC 13032 uid61611
Corynebacterium glutamicum R uid58897
Corynebacterium glutamicum SCgG1 uid207285
Corynebacterium glutamicum SCgG2 uid207286
Corynebacterium jeikeium K411 uid58399
Corynebacterium pseudotuberculosis 316 uid89381
Corynebacterium pseudotuberculosis 31 uid162167
Corynebacterium resistens DSM 45100 uid50555
Corynebacterium terpenotabidum Y 11 uid210639
Corynebacterium ulcerans 0102 uid169879
Corynebacterium ulcerans 809 uid159659
Corynebacterium ulcerans BR AD22 uid68291
Corynebacterium urealyticum DSM 7109 uid61639
Corynebacterium urealyticum DSM 7111 uid188688
Corynebacterium variabile DSM 44702 uid62003
Coxiella burnetii CbuG Q212 uid58893
Coxiella burnetii CbuK Q154 uid58895
Coxiella burnetii Dugway 5J108 111 uid58629
Coxiella burnetii RSA 331 uid58637
Coxiella burnetii RSA 493 uid57631
Crinalium epipsammum PCC 9333 uid183113
Croceibacter atlanticus HTCC2559 uid49661
Cronobacter sakazakii ATCC BAA 894 uid58145

Selected prokaryotic genomes
Cronobacter sakazakii ES15 uid167045
Cronobacter sakazakii Sp291 uid189241
Cronobacter turicensis z3032 uid40821
Cryptobacterium curtum DSM 15641 uid59041
Cupriavidus metallidurans CH34 uid57815
Cupriavidus necator N 1 uid68689
Cupriavidus taiwanensis LMG 19424 uid61615
Cyanobacterium PCC 10605 uid183340
Cyanobacterium stanieri PCC 7202 uid183337
cyanobacterium UCYN A uid43697
Cyanobium gracile PCC 6307 uid182931
Cyanothece ATCC 51142 uid59013
Cyanothece PCC 7424 uid59025
Cyanothece PCC 7425 uid59435
Cyanothece PCC 7822 uid52547
Cyanothece PCC 8801 uid59027
Cyanothece PCC 8802 uid59143
Cyclobacterium marinum DSM 745 uid71485
Cycloclasticus P1 uid176368
Cylindrospermum stagnale PCC 7417 uid183111
Cytophaga hutchinsonii ATCC 33406 uid57651
Dactylococcopsis salina PCC 8305 uid183341
Dechloromonas aromatica RCB uid58025
Dechlorosoma suillum PS uid81439
Deferribacter desulfuricans SSM1 uid46653
Dehalobacter 11DCA uid177715
Dehalobacter CF uid177714

**Selected prokaryotic genomes**
Dehalococcoides BAV1 uid58477
Dehalococcoides CBDB1 uid58413
Dehalococcoides ethenogenes 195 uid57763
Dehalococcoides GT uid42115
Dehalococcoides mccartyi BTF08 uid190183
Dehalococcoides mccartyi DCMB5 uid190184
Dehalococcoides VS uid42393
Deinococcus deserti VCD115 uid58615
Deinococcus geothermalis DSM 11300 uid58275
Deinococcus gobiensis I 0 uid162509
Deinococcus maricopensis DSM 21211 uid62225
Deinococcus peraridilitoris DSM 19664 uid183485
Deinococcus proteolyticus MRP uid63399
Deinococcus radiodurans R1 uid57665
Delftia acidovorans SPH 1 uid58703
Delftia Cs1 4 uid67319
Denitrovibrio acetiphilus DSM 12809 uid46657
Desulfarculus baarsii DSM 2075 uid51371
Desulfatibacillum alkenivorans AK 01 uid58913
Desulfitobacterium hafniense DCB 2 uid57749
Desulfitobacterium hafniense Y51 uid58605
Desulfobacca acetoxidans DSM 11109 uid65785
Desulfobacterium autotrophicum HRM2 uid59061
Desulfobacula toluolica Tol2 uid175777
Desulfobulbus propionicus DSM 2032 uid62265
Desulfocapsa sulfexigens DSM 10523 uid189952
Desulfococcus oleovorans Hxd3 uid58777

Selected prokaryotic genomes
Desulfohalobium retbaense DSM 5692 uid59183
Desulfomicrobium baculatum DSM 4028 uid59217
Desulfomonile tiedjei DSM 6799 uid168320
Desulfosporosinus acidiphilus SJ4 uid156759
Desulfosporosinus meridiei DSM 13257 uid75097
Desulfosporosinus orientis DSM 765 uid82939
Desulfotalea psychrophila LSv54 uid58153
Desulfotomaculum acetoxidans DSM 771 uid59109
Desulfotomaculum gibsoniae DSM 7213 uid76945
Desulfotomaculum kuznetsovii DSM 6115 uid67357
Desulfotomaculum reducens MI 1 uid58277
Desulfotomaculum ruminis DSM 2154 uid67507
Desulfovibrio aespoeensis Aspo 2 uid42613
Desulfovibrio africanus Walvis Bay uid66847
Desulfovibrio alaskensis G20 uid57941
Desulfovibrio desulfuricans ATCC 27774 uid59213
Desulfovibrio desulfuricans ND132 uid63159
Desulfovibrio magneticus RS 1 uid59309
Desulfovibrio piezophilus C1TLV30 uid190704
Desulfovibrio salexigens DSM 2638 uid59223
Desulfovibrio vulgaris DP4 uid58679
Desulfovibrio vulgaris Hildenborough uid57645
Desulfovibrio vulgaris Miyazaki F uid59089
Desulfovibrio vulgaris RCH1 uid161961
Desulfurispirillum indicum S5 uid45897
Desulfurivibrio alkaliphilus AHT2 uid49487
Desulfurococcus fermentans DSM 16532 uid75119

Selected prokaryotic genomes
Desulfurococcus kamchatkensis 1221n uid59133
Desulfurococcus mucosus DSM 2162 uid62227
Dichelobacter nodosus VCS1703A uid57643
Dickeya dadantii 3937 uid52537
Dickeya dadantii Ech586 uid42519
Dickeya dadantii Ech703 uid59363
Dickeya zeae Ech1591 uid59297
Dictyoglomus thermophilum H 6 12 uid59439
Dictyoglomus turgidum DSM 6724 uid59177
Dinoroseobacter shibae DFL 12 uid58707
Dyadobacter fermentans DSM 18053 uid59049
Echinicola vietnamensis DSM 17526 uid184076
Edwardsiella ictaluri 93 146 uid59403
Edwardsiella tarda C07 087 uid193773
Edwardsiella tarda EIB202 uid41819
Edwardsiella tarda FL6 60 uid159657
Eggerthella lenta DSM 2243 uid59079
Eggerthella YY7918 uid68707
Ehrlichia canis Jake uid58071
Ehrlichia chaffeensis Arkansas uid57933
Ehrlichia ruminantium Gardel uid58245
Ehrlichia ruminantium Welgevonden uid58013
Ehrlichia ruminantium Welgevonden uid58243
Elusimicrobium minutum Pei191 uid58949
Emticicia oligotrophica DSM 17448 uid177079
Enterobacter 638 uid58727
Enterobacter aerogenes EA1509E uid187411

Selected prokaryotic genomes
Enterobacter aerogenes KCTC 2190 uid68103
Enterobacter asburiae LF7a uid72793
Enterobacter cloacae ATCC 13047 uid48363
Enterobacter cloacae dissolvens SDM uid168997
Enterobacter cloacae EcWSU1 uid80739
Enterobacter cloacae ENHKU01 uid172463
Enterobacter cloacae NCTC 9394 uid197202
Enterobacter cloacae SCF1 uid59969
Enterobacteriaceae bacterium FGI 57 uid185181
Enterobacter R4 368 uid208672
Enterococcus 7L76 uid197170
Enterococcus casseliflavus EC20 uid55693
Enterococcus faecalis D32 uid171261
Enterococcus faecalis OG1RF uid54927
Enterococcus faecalis Symbioflor 1 uid183342
Enterococcus faecalis V583 uid57669
Enterococcus faecium Aus0004 uid87025
Enterococcus faecium DO uid55353
Enterococcus faecium NRRL B 2354 uid188477
Enterococcus hirae ATCC 9790 uid70619
Erwinia amylovora ATCC 49946 uid46943
Erwinia amylovora CFBP1430 uid46839
Erwinia billingiae Eb661 uid50547
Erwinia Ejp617 uid159955
Erwinia pyrifoliae DSM 12163 uid159693
Erwinia pyrifoliae Ep1 96 uid40659
Erwinia tasmaniensis Et1 99 uid59029

Selected prokaryotic genomes
Erysipelothrix rhusiopathiae Fujisawa uid68021
Erysipelothrix rhusiopathiae SY1027 uid206518
Erythrobacter litoralis HTCC2594 uid58299
Escherichia blattae DSM 4481 uid165043
Escherichia coli 042 uid161985
Escherichia coli 536 uid58531
Escherichia coli 55989 uid59383
Escherichia coli ABU 83972 uid161975
Escherichia coli APEC O1 uid58623
Escherichia coli APEC O78 uid187277
Escherichia coli ATCC 8739 uid58783
Escherichia coli BL21 DE3 uid161947
Escherichia coli BL21 DE3 uid161949
Escherichia coli B REL606 uid58803
Escherichia coli BW2952 uid59391
Escherichia coli CFT073 uid57915
Escherichia coli clone D i14 uid162049
Escherichia coli clone D i2 uid162047
Escherichia coli DH1 uid161951
Escherichia coli DH1 uid162051
Escherichia coli E24377A uid58395
Escherichia coli ED1a uid59379
Escherichia coli ETEC H10407 uid161993
Escherichia coli HS uid58393
Escherichia coli IAI1 uid59377
Escherichia coli IAI39 uid59381
Escherichia coli IHE3034 uid162007

Selected prokaryotic genomes
Escherichia coli K 12 substr DH10B uid58979
Escherichia coli K 12 substr MDS42 uid193705
Escherichia coli K 12 substr MG1655 uid57779
Escherichia coli K 12 substr W3110 uid161931
Escherichia coli KO11FL uid162099
Escherichia coli KO11FL uid52593
Escherichia coli LF82 uid161965
Escherichia coli NA114 uid162139
Escherichia coli O103 H2 12009 uid41013
Escherichia coli O104 H4 2009EL 2050 uid175905
Escherichia coli O104 H4 2009EL 2071 uid176128
Escherichia coli O104 H4 2011C 3493 uid176127
Escherichia coli O111 H 11128 uid41023
Escherichia coli O127 H6 E2348 69 uid59343
Escherichia coli O157 H7 EC4115 uid59091
Escherichia coli O157 H7 EDL933 uid57831
Escherichia coli O157 H7 Sakai uid57781
Escherichia coli O157 H7 TW14359 uid59235
Escherichia coli O26 H11 11368 uid41021
Escherichia coli O55 H7 CB9615 uid46655
Escherichia coli O55 H7 RM12579 uid162153
Escherichia coli O7 K1 CE10 uid162115
Escherichia coli O83 H1 NRG 857C uid161987
Escherichia coli P12b uid162061
Escherichia coli S88 uid62979
Escherichia coli SE11 uid59425
Escherichia coli SE15 uid161939

**Selected prokaryotic genomes**
Escherichia coli SMS 3 5 uid58919
Escherichia coli UM146 uid162043
Escherichia coli UMN026 uid62981
Escherichia coli UMNK88 uid161991
Escherichia coli UTI89 uid58541
Escherichia coli W uid162011
Escherichia coli W uid162101
Escherichia coli Xuzhou21 uid163995
Escherichia fergusonii ATCC 35469 uid59375
Ethanoligenens harbinense YUAN 3 uid46255
Eubacterium cylindroides T2 87 uid197177
Eubacterium eligens ATCC 27750 uid59171
Eubacterium limosum KIST612 uid59777
Eubacterium rectale ATCC 33656 uid59169
Eubacterium rectale uid197161
Eubacterium rectale uid197162
Eubacterium siraeum uid197160
Eubacterium siraeum V10Sc8a uid197178
Exiguobacterium antarcticum B7 uid176125
Exiguobacterium AT1b uid59093
Exiguobacterium sibiricum 255 15 uid58053
Faecalibacterium prausnitzii L2 6 uid197183
Faecalibacterium prausnitzii uid197157
Ferrimonas balearica DSM 9799 uid53371
Ferroglobus placidus DSM 10642 uid40863
Ferroplasma acidarmanus fer1 uid54095
Fervidicoccus fontis Kam940 uid162201

Selected prokaryotic genomes
Fervidobacterium nodosum Rt17 B1 uid58625
Fervidobacterium pennivorans DSM 9078 uid78143
Fibrella aestuarina BUZ 2 uid178352
Fibrobacter succinogenes S85 uid161919
Fibrobacter succinogenes S85 uid41169
Filifactor alocis ATCC 35896 uid46625
Finegoldia magna ATCC 29328 uid58867
Flavobacteriaceae bacterium 3519 10 uid59413
Flavobacteriales bacterium HTCC2170 uid51877
Flavobacterium branchiophilum FL 15 uid73421
Flavobacterium columnare ATCC 49512 uid80731
Flavobacterium indicum GPTSA100 9 uid157999
Flavobacterium johnsoniae UW101 uid58493
Flavobacterium psychrophilum JIP02 86 uid61627
Flexibacter litoralis DSM 6794 uid168257
Flexistipes sinusarabici DSM 4947 uid68147
Fluviicola taffensis DSM 16823 uid65271
Francisella cf novicida 3523 uid162107
Francisella cf novicida Fx1 uid162105
Francisella novicida U112 uid58499
Francisella philomiragia ATCC 25017 uid59105
Francisella tularensis FSC198 uid58693
Francisella tularensis holarctica F92 uid181998
Francisella tularensis holarctica LVS uid58595
Francisella tularensis NE061598 uid161973
Francisella tularensis SCHU S4 uid57589
Francisella tularensis TI0902 uid89373

Selected prokaryotic genomes
Francisella tularensis TIGB03 uid89379
Francisella tularensis WY96 3418 uid58811
Francisella TX077308 uid68321
Frankia alni ACN14a uid58695
Frankia CcI3 uid58397
Frankia EAN1pec uid58367
Frankia EuI1c uid42615
Frankia symbiont of Datisca glomerata uid46257
Frateuria aurantia DSM 6220 uid81775
Fusobacterium 4 8 uid205051
Fusobacterium nucleatum ATCC 25586 uid57885
Gallibacterium anatis UMN179 uid66567
Gallionella capsiferriformans ES 2 uid51505
gamma proteobacterium HdN1 uid51635
Gardnerella vaginalis 409 05 uid43211
Gardnerella vaginalis ATCC 14019 uid55487
Gardnerella vaginalis HMP9231 uid162045
Geitlerinema PCC 7407 uid183007
Gemmatimonas aurantiaca T 27 uid58813
Geobacillus C56 T3 uid49467
Geobacillus HH01 uid188479
Geobacillus kaustophilus HTA426 uid58227
Geobacillus thermodenitrificans NG80 2 uid58829
Geobacillus WCH70 uid59045
Geobacillus Y412MC52 uid55381
Geobacillus Y412MC61 uid41171
Geobacillus Y4 1MC1 uid55779

Selected prokaryotic genomes
Geobacter bemidjiensis Bem uid58749
Geobacter FRC 32 uid58543
Geobacter lovleyi SZ uid58713
Geobacter M18 uid55771
Geobacter M21 uid59037
Geobacter metallireducens GS 15 uid57731
Geobacter sulfurreducens KN400 uid161977
Geobacter sulfurreducens PCA uid57743
Geobacter uraniireducens Rf4 uid58475
Geodermatophilus obscurus DSM 43160 uid43725
Glaciecola 4H 3 7 YE 5 uid66595
Glaciecola nitratireducens FR1064 uid73759
Glaciecola psychrophila 170 uid193711
Gloeobacter violaceus PCC 7421 uid58011
Gloeocapsa PCC 7428 uid183112
Gluconacetobacter diazotrophicus PAl 5 uid59075
Gluconacetobacter diazotrophicus PAl 5 uid61587
Gluconacetobacter xylinus NBRC 3288 uid46523
Gluconobacter oxydans 621H uid58239
Gluconobacter oxydans H24 uid179202
Gordonia bronchialis DSM 43247 uid41403
Gordonia KTR9 uid174812
Gordonia polyisoprenivorans VH2 uid86651
Gordonibacter pamelaeae 7 10 1 b uid197167
Gramella forsetii KT0803 uid58881
Granulibacter bethesdensis CGDNIH1 uid58661
Granulicella mallensis MP5ACTX8 uid49957

Selected prokaryotic genomes
Haemophilus ducreyi 35000HP uid57625
Haemophilus influenzae 10810 uid86647
Haemophilus influenzae 86 028NP uid58093
Haemophilus influenzae F3031 uid62123
Haemophilus influenzae F3047 uid62097
Haemophilus influenzae PittEE uid58591
Haemophilus influenzae PittGG uid58593
Haemophilus influenzae R2846 uid161921
Haemophilus influenzae R2866 uid161923
Haemophilus influenzae Rd KW20 uid57771
Haemophilus parainfluenzae T3T1 uid72801
Haemophilus parasuis SH0165 uid59273
Haemophilus parasuis ZJ0906 uid209117
Haemophilus somnus 129PT uid57929
Haemophilus somnus 2336 uid57979
Hahella chejuensis KCTC 2396 uid58483
Halalkalicoccus jeotgali B3 uid50305
Halanaerobium hydrogeniformans uid60191
Halanaerobium praevalens DSM 2228 uid161959
Haliangium ochraceum DSM 14365 uid41425
Haliscomenobacter hydrossis DSM 1100 uid66777
Haloarcula hispanica ATCC 33960 uid72475
Haloarcula marismortui ATCC 43049 uid57719
Halobacillus halophilus DSM 2266 uid162033
Halobacterium NRC 1 uid57769
Halobacterium salinarum R1 uid61571
Halobacteroides halobius DSM 5150 uid184862

Selected prokaryotic genomes
Haloferax mediterranei ATCC 33500 uid167315
Haloferax volcanii DS2 uid46845
Halogeometricum borinquense DSM 11551 uid54919
Halomicrobium mukohataei DSM 12286 uid59107
Halomonas elongata DSM 2581 uid52781
halophilic archaeon DL31 uid72619
Halopiger xanaduensis SH 6 uid68105
Haloquadratum walsbyi C23 uid162019
Haloquadratum walsbyi DSM 16790 uid58673
Halorhabdus utahensis DSM 12940 uid59189
Halorhodospira halophila SL1 uid58473
Halorubrum lacusprofundi ATCC 49239 uid58807
Haloterrigena turkmenica DSM 5511 uid43501
Halothece PCC 7418 uid183338
Halothermothrix orenii H 168 uid58585
Halothiobacillus neapolitanus c2 uid41317
Halovivax ruber XH 70 uid184819
Helicobacter acinonychis Sheeba uid58685
Helicobacter bizzozeronii CIII 1 uid68141
Helicobacter cetorum MIT 00 7128 uid162217
Helicobacter cetorum MIT 99 5656 uid162215
Helicobacter cinaedi ATCC BAA 847 uid193765
Helicobacter cinaedi PAGU611 uid162219
Helicobacter felis ATCC 49179 uid61409
Helicobacter heilmannii ASB1 4 uid182935
Helicobacter hepaticus ATCC 51449 uid57737
Helicobacter mustelae 12198 uid46647

Selected prokaryotic genomes
Helicobacter pylori 2017 uid161151
Helicobacter pylori 2018 uid161159
Helicobacter pylori 26695 uid178201
Helicobacter pylori 26695 uid57787
Helicobacter pylori 35A uid49903
Helicobacter pylori 51 uid161925
Helicobacter pylori 83 uid161153
Helicobacter pylori 908 uid159985
Helicobacter pylori Aklavik117 uid182201
Helicobacter pylori Aklavik86 uid182202
Helicobacter pylori B38 uid59415
Helicobacter pylori B8 uid49873
Helicobacter pylori Cuz20 uid159987
Helicobacter pylori ELS37 uid158157
Helicobacter pylori F16 uid161145
Helicobacter pylori F30 uid159991
Helicobacter pylori F32 uid161139
Helicobacter pylori F57 uid161143
Helicobacter pylori G27 uid59305
Helicobacter pylori Gambia94 24 uid159493
Helicobacter pylori HPAG1 uid58517
Helicobacter pylori HUP B14 uid162213
Helicobacter pylori India7 uid161149
Helicobacter pylori J99 uid57789
Helicobacter pylori Lithuania75 uid159491
Helicobacter pylori OK113 uid193715
Helicobacter pylori OK310 uid193716

Selected prokaryotic genomes
Helicobacter pylori P12 uid59327
Helicobacter pylori PeCan18 uid162211
Helicobacter pylori PeCan4 uid53539
Helicobacter pylori Puno120 uid159611
Helicobacter pylori Puno135 uid161157
Helicobacter pylori Rif1 uid178202
Helicobacter pylori Rif2 uid178203
Helicobacter pylori Sat464 uid159467
Helicobacter pylori Shi112 uid162207
Helicobacter pylori Shi169 uid162209
Helicobacter pylori Shi417 uid162205
Helicobacter pylori Shi470 uid59165
Helicobacter pylori SJM180 uid53541
Helicobacter pylori SNT49 uid159615
Helicobacter pylori SouthAfrica7 uid159989
Helicobacter pylori uid159983
Helicobacter pylori UM032 uid203025
Helicobacter pylori UM037 uid203027
Helicobacter pylori UM066 uid203028
Helicobacter pylori UM298 uid213226
Helicobacter pylori UM299 uid203026
Helicobacter pylori v225d uid159639
Helicobacter pylori XZ274 uid165869
Heliobacterium modesticaldum Ice1 uid58279
Herbaspirillum seropedicae SmR1 uid50427
Herminiimonas arsenicoxydans uid58291
Herpetosiphon aurantiacus DSM 785 uid58599

Selected prokaryotic genomes
Hippea maritima DSM 10411 uid65267
Hirschia baltica ATCC 49814 uid59365
Hydrogenobacter thermophilus TK 6 uid159875
Hydrogenobacter thermophilus TK 6 uid45927
Hydrogenobaculum HO uid190882
Hydrogenobaculum SN uid46251
Hydrogenobaculum Y04AAS1 uid58857
Hyperthermus butylicus DSM 5456 uid57755
Hyphomicrobium denitrificans 1NES1 uid179904
Hyphomicrobium MC1 uid68453
Hyphomonas neptunium ATCC 15444 uid58433
Idiomarina loihiensis GSL 199 uid205256
Idiomarina loihiensis L2TR uid58087
Ignavibacterium album JCM 16511 uid162097
Ignicoccus hospitalis KIN4 I uid58365
Ignisphaera aggregans DSM 17230 uid51875
Ilyobacter polytropus DSM 2926 uid59769
Intrasporangium calvum DSM 43043 uid61729
Isoptericola variabilis 225 uid67501
Isosphaera pallida ATCC 43644 uid62207
Jannaschia CCS1 uid58147
Janthinobacterium Marseille uid58603
Jonesia denitrificans DSM 20603 uid59053
Kangiella koreensis DSM 16069 uid59209
Ketogulonicigenium vulgare WSH 001 uid161161
Ketogulonicigenium vulgare Y25 uid59581
Kineococcus radiotolerans SRS30216 uid58067

Selected prokaryotic genomes
Kitasatospora setae KM 6054 uid77027
Klebsiella oxytoca E718 uid170256
Klebsiella oxytoca KCTC 1686 uid83159
Klebsiella pneumoniae 1084 uid174151
Klebsiella pneumoniae 342 uid59145
Klebsiella pneumoniae HS11286 uid84387
Klebsiella pneumoniae KCTC 2242 uid162147
Klebsiella pneumoniae MGH 78578 uid57619
Klebsiella pneumoniae NTUH K2044 uid59073
Klebsiella variicola At 22 uid42113
Kocuria rhizophila DC2201 uid59099
Kosmotoga olearia TBF 19 5 1 uid59205
Kribbella flavida DSM 17836 uid43465
Krokinobacter 4H 3 7 5 uid66593
Kyrpidia tusciae DSM 2912 uid48361
Kytococcus sedentarius DSM 20547 uid59071
Lacinutrix 5H 3 7 4 uid68067
Lactobacillus acidophilus 30SC uid63605
Lactobacillus acidophilus La 14 uid201479
Lactobacillus acidophilus NCFM uid57685
Lactobacillus amylovorus GRL 1112 uid61179
Lactobacillus amylovorus GRL1118 uid160233
Lactobacillus brevis ATCC 367 uid57989
Lactobacillus brevis KB290 uid195560
Lactobacillus buchneri NRRL B 30929 uid66205
Lactobacillus buchneri uid73657
Lactobacillus casei ATCC 334 uid57985

Selected prokaryotic genomes
Lactobacillus casei BD II uid162119
Lactobacillus casei BL23 uid59237
Lactobacillus casei LC2W uid162121
Lactobacillus casei LOCK919 uid210959
Lactobacillus casei W56 uid178736
Lactobacillus casei Zhang uid50673
Lactobacillus crispatus ST1 uid48359
Lactobacillus fermentum CECT 5716 uid162003
Lactobacillus fermentum F6 uid203391
Lactobacillus fermentum IFO 3956 uid58865
Lactobacillus gasseri ATCC 33323 uid57687
Lactobacillus helveticus CNRZ32 uid212302
Lactobacillus helveticus DPC 4571 uid58761
Lactobacillus helveticus H10 uid162017
Lactobacillus helveticus R0052 uid174439
Lactobacillus johnsonii DPC 6026 uid162057
Lactobacillus johnsonii FI9785 uid41735
Lactobacillus johnsonii NCC 533 uid58029
Lactobacillus kefiranofaciens ZW3 uid67985
Lactobacillus plantarum 16 uid209042
Lactobacillus plantarum JDM1 uid59361
Lactobacillus plantarum P8 uid203333
Lactobacillus plantarum ST III uid53537
Lactobacillus plantarum WCFS1 uid62911
Lactobacillus plantarum ZJ316 uid188689
Lactobacillus reuteri DSM 20016 uid58471
Lactobacillus reuteri I5007 uid208677

Selected prokaryotic genomes
Lactobacillus reuteri JCM 1112 uid58875
Lactobacillus reuteri SD2112 uid55357
Lactobacillus reuteri TD1 uid213089
Lactobacillus rhamnosus ATCC 8530 uid162169
Lactobacillus rhamnosus GG uid161983
Lactobacillus rhamnosus GG uid59313
Lactobacillus rhamnosus Lc 705 uid59315
Lactobacillus rhamnosus LOCK900 uid210957
Lactobacillus rhamnosus LOCK908 uid210958
Lactobacillus ruminis ATCC 27782 uid73417
Lactobacillus sakei 23K uid58281
Lactobacillus salivarius CECT 5713 uid162005
Lactobacillus salivarius UCC118 uid58233
Lactococcus garvieae ATCC 49156 uid73413
Lactococcus garvieae Lg2 uid161935
Lactococcus lactis cremoris A76 uid160937
Lactococcus lactis cremoris MG1363 uid58837
Lactococcus lactis cremoris NZ9000 uid167481
Lactococcus lactis cremoris SK11 uid57983
Lactococcus lactis cremoris UC509 9 uid179384
Lactococcus lactis CV56 uid160253
Lactococcus lactis Il1403 uid57671
Lactococcus lactis IO 1 uid192185
Lactococcus lactis KF147 uid42831
Laribacter hongkongensis HLHK9 uid59265
Lawsonia intracellularis N343 uid186598
Lawsonia intracellularis PHE MN1 00 uid61575

Selected prokaryotic genomes
Leadbetterella byssophila DSM 17132 uid60161
Legionella longbeachae NSW150 uid46099
Legionella pneumophila 2300 99 Alcoy uid48801
Legionella pneumophila ATCC 43290 uid86885
Legionella pneumophila Corby uid58733
Legionella pneumophila HL06041035 uid170534
Legionella pneumophila Lens uid58209
Legionella pneumophila Lorraine uid170535
Legionella pneumophila Paris uid58211
Legionella pneumophila Philadelphia 1 uid193710
Legionella pneumophila Philadelphia 1 uid57609
Legionella pneumophila Thunder Bay uid206517
Leifsonia xyli CTCB07 uid57759
Leptolyngbya PCC 7376 uid182928
Leptospirillum ferriphilum ML 04 uid175904
Leptospirillum ferrooxidans C2 3 uid158171
Leptothrix cholodnii SP 6 uid58971
Leptotrichia buccalis C 1013 b uid59211
Leuconostoc C2 uid68743
Leuconostoc carnosum JB16 uid176371
Leuconostoc citreum KM20 uid58481
Leuconostoc gasicomitatum LMG 18811 uid50385
Leuconostoc gelidum JB7 uid175682
Leuconostoc kimchii IMSNU 11154 uid48589
Leuconostoc mesenteroides ATCC 8293 uid57919
Leuconostoc mesenteroides J18 uid84337
Listeria innocua Clip11262 uid61567

Selected prokaryotic genomes
Listeria ivanovii PAM 55 uid73473
Listeria monocytogenes 07PF0776 uid162185
Listeria monocytogenes 08 5923 uid43727
Listeria monocytogenes 10403S uid54461
Listeria monocytogenes ATCC 19117 uid175109
Listeria monocytogenes Clip80459 uid59317
Listeria monocytogenes EGD e uid61583
Listeria monocytogenes Finland 1998 uid54443
Listeria monocytogenes FSL R2 561 uid54441
Listeria monocytogenes HCC23 uid59203
Listeria monocytogenes J0161 uid54459
Listeria monocytogenes J1 220 uid179735
Listeria monocytogenes L312 uid175768
Listeria monocytogenes La111 uid193768
Listeria monocytogenes M7 uid162131
Listeria monocytogenes N53 1 uid193767
Listeria monocytogenes SLCC2372 uid174872
Listeria monocytogenes SLCC2376 uid175111
Listeria monocytogenes SLCC2378 uid175105
Listeria monocytogenes SLCC2479 uid175108
Listeria monocytogenes SLCC2540 uid175106
Listeria monocytogenes SLCC5850 uid175110
Listeria monocytogenes SLCC7179 uid175107
Listeria monocytogenes uid212977
Listeria monocytogenes uid43671
Lysinibacillus sphaericus C3 41 uid58945
Macrococcus caseolyticus JCSC5402 uid59003

**Selected prokaryotic genomes**
Magnetococcus MC 1 uid57833
Magnetospirillum magneticum AMB 1 uid58527
Mahella australiensis 50 1 BON uid66917
Mannheimia haemolytica D153 uid212303
Mannheimia haemolytica D171 uid212304
Mannheimia haemolytica D174 uid212305
Mannheimia haemolytica M42548 uid198769
Mannheimia haemolytica USMARC 2286 uid213228
Mannheimia succiniciproducens MBEL55E uid58197
Maricaulis maris MCS10 uid58689
Marinithermus hydrothermalis DSM 14884 uid65783
Marinitoga piezophila KA3 uid81629
Marinobacter adhaerens HP15 uid162009
Marinobacter aquaeolei VT8 uid59419
Marinobacter BSs20148 uid171995
Marinomonas mediterranea MMB 1 uid64753
Marinomonas MWYL1 uid58715
Marinomonas posidonica IVIA Po 181 uid67323
Marivirga tractuosa DSM 4126 uid60837
Megamonas hypermegale uid197163
Megasphaera elsdenii DSM 20460 uid71135
Meiothermus ruber DSM 1279 uid198526
Meiothermus ruber DSM 1279 uid46661
Meiothermus silvanus DSM 9946 uid49485
Melioribacter roseus P3M uid170941
Melissococcus plutonius ATCC 35311 uid66803
Melissococcus plutonius DAT561 uid89371

Selected prokaryotic genomes
Mesoplasma florum L1 uid58055
Mesorhizobium australicum WSM2073 uid75101
Mesorhizobium loti MAFF303099 uid57601
Mesorhizobium opportunistum WSM2075 uid40861
Mesotoga prima MesG1 Ag 4 2 uid52599
Metallosphaera cuprina Ar 4 uid66329
Metallosphaera sedula DSM 5348 uid58717
Methanobacterium AL 21 uid63623
Methanobacterium SWAN 1 uid67359
Methanobrevibacter AbM4 uid206516
Methanobrevibacter ruminantium M1 uid45857
Methanobrevibacter smithii ATCC 35061 uid58827
Methanocaldococcus fervens AG86 uid59347
Methanocaldococcus FS406 22 uid42499
Methanocaldococcus infernus ME uid48803
Methanocaldococcus jannaschii DSM 2661 uid57713
Methanocaldococcus vulcanius M7 uid41131
Methanocella arvoryzae MRE50 uid61623
Methanocella conradii HZ254 uid157911
Methanocella paludicola SANAE uid42887
Methanococcoides burtonii DSM 6242 uid58023
Methanococcus aeolicus Nankai 3 uid58823
Methanococcus maripaludis C5 uid58741
Methanococcus maripaludis C6 uid58947
Methanococcus maripaludis C7 uid58847
Methanococcus maripaludis S2 uid58035
Methanococcus maripaludis X1 uid70729

Selected prokaryotic genomes
Methanococcus vannielii SB uid58767
Methanococcus voltae A3 uid49529
Methanocorpusculum labreanum Z uid58785
Methanoculleus bourgensis MS2 uid171377
Methanoculleus marisnigri JR1 uid58561
Methanohalobium evestigatum Z 7303 uid49857
Methanohalophilus mahii DSM 5219 uid47313
Methanolobus psychrophilus R15 uid177925
Methanomassiliicoccus Mx1 Issoire uid207287
Methanoplanus petrolearius DSM 11571 uid52695
Methanopyrus kandleri AV19 uid57883
Methanoregula boonei 6A8 uid58815
Methanoregula formicicum SMSP uid184406
Methanosaeta concilii GP6 uid66207
Methanosaeta harundinacea 6Ac uid81199
Methanosaeta thermophila PT uid58469
Methanosalsum zhilinae DSM 4017 uid68249
Methanosarcina acetivorans C2A uid57879
Methanosarcina barkeri Fusaro uid57715
Methanosarcina mazei Go1 uid57893
Methanosarcina mazei Tuc01 uid190185
Methanosphaera stadtmanae DSM 3091 uid58407
Methanosphaerula palustris E1 9c uid59193
Methanospirillum hungatei JF 1 uid58181
Methanothermococcus okinawensis IH1 uid51535
Methanothermus fervidus DSM 2088 uid60167
Methanotorris igneus Kol 5 uid67321

Selected prokaryotic genomes
Methylacidiphilum infernorum V4 uid59161
Methylibium petroleiphilum PM1 uid58085
Methylobacillus flagellatus KT uid58049
Methylobacterium 4 46 uid58843
Methylobacterium chloromethanicum CM4 uid58933
Methylobacterium extorquens AM1 uid57605
Methylobacterium extorquens DM4 uid61617
Methylobacterium extorquens PA1 uid58821
Methylobacterium nodulans ORS 2060 uid59023
Methylobacterium populi BJ001 uid58937
Methylocella silvestris BL2 uid59433
Methylococcus capsulatus Bath uid57607
Methylocystis SC2 uid174072
Methylomicrobium alcaliphilum uid77119
Methylomonas methanica MC09 uid67363
Methylophaga JAM1 uid162947
Methylophaga JAM7 uid162949
Methylotenera 301 uid49469
Methylotenera mobilis JLW8 uid59373
Methylovorus glucosetrophus SIP3 4 uid59367
Methylovorus MP688 uid60723
Micavibrio aeruginosavorus ARL 13 uid73585
Micavibrio EPB uid194120
Microbacterium testaceum StLB037 uid62789
Micrococcus luteus NCTC 2665 uid59033
Microcoleus PCC 7113 uid183114
Microcystis aeruginosa NIES 843 uid59101

Selected prokaryotic genomes
Microlunatus phosphovorus NM 1 uid68055
Micromonospora aurantiaca ATCC 27029 uid42501
Micromonospora L5 uid45895
Mobiluncus curtisii ATCC 43063 uid49695
Modestobacter marinus uid167487
Moorella thermoacetica ATCC 39073 uid58051
Moraxella catarrhalis BBH18 uid48809
Morganella morganii KT uid180867
Muricauda ruestringensis DSM 13258 uid72479
Mycobacterium abscessus uid61613
Mycobacterium africanum GM041182 uid68839
Mycobacterium avium 104 uid57693
Mycobacterium bovis AF2122 97 uid57695
Mycobacterium bovis BCG Korea 1168P uid189029
Mycobacterium bovis BCG Mexico uid86889
Mycobacterium bovis BCG Pasteur 1173P2 uid58781
Mycobacterium bovis BCG Tokyo 172 uid59281
Mycobacterium canettii CIPT 140010059 uid70731
Mycobacterium canettii CIPT 140060008 uid184829
Mycobacterium canettii CIPT 140070008 uid184832
Mycobacterium canettii CIPT 140070010 uid184828
Mycobacterium canettii CIPT 140070017 uid184830
Mycobacterium chubuense NBB4 uid168322
Mycobacterium gilvum PYR GCK uid59421
Mycobacterium gilvum Spyr1 uid61403
Mycobacterium intracellulare MOTT 02 uid89387
Mycobacterium intracellulare MOTT 64 uid89385

Selected prokaryotic genomes
Mycobacterium JDM601 uid67369
Mycobacterium JLS uid58489
Mycobacterium KMS uid58491
Mycobacterium leprae Br4923 uid59293
Mycobacterium leprae TN uid57697
Mycobacterium liflandii 128FXT uid59005
Mycobacterium marinum M uid59423
Mycobacterium massiliense GO 06 uid170732
Mycobacterium MCS uid58465
Mycobacterium MOTT36Y uid164001
Mycobacterium rhodesiae NBB3 uid75107
Mycobacterium smegmatis JS623 uid184820
Mycobacterium smegmatis MC2 155 uid171958
Mycobacterium smegmatis MC2 155 uid57701
Mycobacterium tuberculosis CCDC5079 uid161943
Mycobacterium tuberculosis CCDC5079 uid203790
Mycobacterium tuberculosis CCDC5180 uid161941
Mycobacterium tuberculosis CDC1551 uid57775
Mycobacterium tuberculosis CTRI 2 uid161997
Mycobacterium tuberculosis EAI5 uid212307
Mycobacterium tuberculosis F11 uid58417
Mycobacterium tuberculosis H37Ra uid58853
Mycobacterium tuberculosis H37Rv uid170532
Mycobacterium tuberculosis H37Rv uid57777
Mycobacterium tuberculosis KZN 1435 uid59069
Mycobacterium tuberculosis KZN 4207 uid83619
Mycobacterium tuberculosis KZN 605 uid54947

Selected prokaryotic genomes
Mycobacterium tuberculosis RGTB327 uid157907
Mycobacterium tuberculosis RGTB423 uid162179
Mycobacterium tuberculosis uid185758
Mycobacterium tuberculosis UT205 uid162183
Mycobacterium ulcerans Agy99 uid62939
Mycobacterium vanbaalenii PYR 1 uid58463
Mycobacterium yongonense 05 1390 uid189649
Mycoplasma agalactiae PG2 uid61619
Mycoplasma agalactiae uid46679
Mycoplasma arthritidis 158L3 1 uid58005
Mycoplasma bovis HB0801 uid168665
Mycoplasma bovis Hubei 1 uid68691
Mycoplasma bovis PG45 uid60859
Mycoplasma capricolum ATCC 27343 uid58525
Mycoplasma conjunctivae uid59325
Mycoplasma crocodyli MP145 uid47087
Mycoplasma cynos C142 uid184824
Mycoplasma fermentans JER uid53543
Mycoplasma fermentans M64 uid62099
Mycoplasma fermentans PG18 uid197154
Mycoplasma gallisepticum F uid162001
Mycoplasma gallisepticum R high uid161999
Mycoplasma gallisepticum R low uid57993
Mycoplasma genitalium G37 uid57707
Mycoplasma genitalium M2288 uid173372
Mycoplasma genitalium M2321 uid173373
Mycoplasma genitalium M6282 uid173371

Selected prokaryotic genomes
Mycoplasma genitalium M6320 uid173370
Mycoplasma haemocanis Illinois uid82367
Mycoplasma haemofelis Langford 1 uid62461
Mycoplasma haemofelis Ohio2 uid162029
Mycoplasma hominis ATCC 23114 uid41875
Mycoplasma hyopneumoniae 168 L uid205052
Mycoplasma hyopneumoniae 168 uid162053
Mycoplasma hyopneumoniae 232 uid58205
Mycoplasma hyopneumoniae 7422 uid212968
Mycoplasma hyopneumoniae 7448 uid58039
Mycoplasma hyopneumoniae J uid58059
Mycoplasma hyorhinis GDL 1 uid87003
Mycoplasma hyorhinis HUB 1 uid51695
Mycoplasma hyorhinis MCLD uid162087
Mycoplasma hyorhinis SK76 uid181997
Mycoplasma leachii 99 014 6 uid162031
Mycoplasma leachii PG50 uid60849
Mycoplasma mobile 163K uid58077
Mycoplasma mycoides capri LC 95010 uid66189
Mycoplasma mycoides SC Gladysdale uid197153
Mycoplasma mycoides SC PG1 uid58031
Mycoplasma penetrans HF 2 uid57729
Mycoplasma pneumoniae 309 uid85495
Mycoplasma pneumoniae FH uid162027
Mycoplasma pneumoniae M129 B7 uid185759
Mycoplasma pneumoniae M129 uid57709
Mycoplasma pulmonis UAB CTIP uid61569

Selected prokaryotic genomes
Mycoplasma putrefaciens KS1 uid72481
Mycoplasma putrefaciens Mput9231 uid198525
Mycoplasma suis Illinois uid61897
Mycoplasma suis KI3806 uid63665
Mycoplasma synoviae 53 uid58061
Mycoplasma wenyonii Massachusetts uid170731
Myxococcus fulvus HW 1 uid68443
Myxococcus stipitatus DSM 14675 uid186549
Myxococcus xanthus DK 1622 uid58003
Nakamurella multipartita DSM 44233 uid59221
Nanoarchaeum equitans Kin4 M uid58009
Natrialba magadii ATCC 43099 uid46245
Natrinema J7 uid171337
Natrinema pellirubrum DSM 15624 uid74437
Natronobacterium gregoryi SP2 uid74439
Natronococcus occultus SP4 uid184863
Natronomonas moolapensis 8 8 11 uid190182
Natronomonas pharaonis DSM 2160 uid58435
Nautilia profundicola AmH uid59345
Neisseria gonorrhoeae FA 1090 uid57611
Neisseria gonorrhoeae NCCP11945 uid59191
Neisseria gonorrhoeae TCDC NG08107 uid161097
Neisseria lactamica 020 06 uid60851
Neisseria meningitidis 053442 uid58587
Neisseria meningitidis 8013 uid161967
Neisseria meningitidis alpha14 uid61649
Neisseria meningitidis alpha710 uid161971

Selected prokaryotic genomes
Neisseria meningitidis FAM18 uid57825
Neisseria meningitidis G2136 uid162085
Neisseria meningitidis H44 76 uid162083
Neisseria meningitidis M01 240149 uid162079
Neisseria meningitidis M01 240355 uid162075
Neisseria meningitidis M04 240196 uid162081
Neisseria meningitidis MC58 uid57817
Neisseria meningitidis NZ 05 33 uid162077
Neisseria meningitidis WUE 2594 uid162093
Neisseria meningitidis Z2491 uid57819
Neorickettsia risticii Illinois uid58889
Neorickettsia sennetsu Miyayama uid57965
Niastella koreensis GR20 10 uid83125
Nitratifractor salsuginis DSM 16511 uid62183
Nitratiruptor SB155 2 uid58861
Nitrobacter hamburgensis X14 uid58293
Nitrobacter winogradskyi Nb 255 uid58295
Nitrosococcus halophilus Nc4 uid46803
Nitrosococcus oceani ATCC 19707 uid58403
Nitrosococcus watsonii C 113 uid50331
Nitrosomonas AL212 uid55727
Nitrosomonas europaea ATCC 19718 uid57647
Nitrosomonas eutropha C91 uid58363
Nitrosomonas Is79A3 uid68745
Nitrosopumilus maritimus SCM1 uid58903
Nitrosospira multiformis ATCC 25196 uid58361
Nocardia brasiliensis ATCC 700358 uid86913

Selected prokaryotic genomes
Nocardia cyriacigeorgica GUH 2 uid89395
Nocardia farcinica IFM 10152 uid58203
Nocardioides JS614 uid58149
Nocardiopsis alba ATCC BAA 2165 uid174334
Nocardiopsis dassonvillei DSM 43111 uid49483
Nostoc azollae 0708 uid49725
Nostoc PCC 7107 uid182932
Nostoc PCC 7120 uid57803
Nostoc PCC 7524 uid182933
Nostoc punctiforme PCC 73102 uid57767
Novosphingobium PP1Y uid67383
Oceanimonas GK1 uid81627
Oceanithermus profundus DSM 14977 uid60855
Oceanobacillus iheyensis HTE831 uid57867
Ochrobactrum anthropi ATCC 49188 uid58921
Octadecabacter antarcticus 307 uid54701
Octadecabacter arcticus 238 uid54699
Odoribacter splanchnicus DSM 20712 uid63397
Oenococcus oeni PSU 1 uid59417
Oligotropha carboxidovorans OM4 uid162135
Oligotropha carboxidovorans OM5 uid59155
Oligotropha carboxidovorans OM5 uid72795
Olsenella uli DSM 7084 uid51367
Onion yellows phytoplasma OY M uid58015
Opitutus terrae PB90 1 uid58965
Orientia tsutsugamushi Boryong uid61621
Orientia tsutsugamushi Ikeda uid58869

Selected prokaryotic genomes
Oscillatoria acuminata PCC 6304 uid183003
Oscillatoria PCC 7112 uid183110
Oscillibacter valericigenes uid73895
Owenweeksia hongkongensis DSM 17368 uid82951
Paenibacillus JDR 2 uid59021
Paenibacillus mucilaginosus 3016 uid89377
Paenibacillus mucilaginosus K02 uid162117
Paenibacillus mucilaginosus KNP414 uid68311
Paenibacillus polymyxa E681 uid53477
Paenibacillus polymyxa M1 uid162159
Paenibacillus polymyxa SC2 uid59583
Paenibacillus terrae HPL 003 uid82371
Paenibacillus Y412MC10 uid41127
Paludibacter propionicigenes WB4 uid60725
Pantoea ananatis AJ13355 uid162073
Pantoea ananatis LMG 20103 uid46807
Pantoea ananatis PA13 uid162181
Pantoea ananatis uid86861
Pantoea At 9b uid55845
Pantoea vagans C9 1 uid49871
Parabacteroides distasonis ATCC 8503 uid58301
Parachlamydia acanthamoebae UV7 uid68335
Paracoccus denitrificans PD1222 uid58187
Parvibaculum lavamentivorans DS 1 uid58739
Parvularcula bermudensis HTCC2503 uid51641
Pasteurella multocida 3480 uid161955
Pasteurella multocida 36950 uid86887

**Selected prokaryotic genomes**
Pasteurella multocida HN06 uid156881
Pasteurella multocida Pm70 uid57627
Pectobacterium atrosepticum SCRI1043 uid57957
Pectobacterium carotovorum PC1 uid59295
Pectobacterium carotovorum PCC21 uid174335
Pectobacterium SCC3193 uid193707
Pectobacterium wasabiae WPP163 uid41297
Pediococcus claussenii ATCC BAA 344 uid81103
Pediococcus pentosaceus ATCC 25745 uid57981
Pedobacter heparinus DSM 2366 uid59111
Pedobacter saltans DSM 12145 uid61349
Pelagibacterium halotolerans B2 uid74393
Pelobacter carbinolicus DSM 2380 uid58241
Pelobacter propionicus DSM 2379 uid58255
Pelodictyon phaeoclathratiforme BU 1 uid58173
Pelotomaculum thermopropionicum SI uid58877
Persephonella marina EX H1 uid58119
Persicivirga dokdonensis DSW 6 uid186842
Petrotoga mobilis SJ95 uid58747
Phaeobacter gallaeciensis 2 10 uid54715
Phaeobacter gallaeciensis DSM 17395 uid54717
Phenylobacterium zucineum HLK1 uid58959
Photobacterium profundum SS9 uid62923
Photorhabdus asymbiotica ATCC 43949 uid59243
Phycisphaera mikurensis NBRC 102666 uid157331
Picrophilus torridus DSM 9790 uid58041
Pirellula staleyi DSM 6068 uid43209

Selected prokaryotic genomes
Planctomyces brasiliensis DSM 5305 uid60583
Planctomyces limnophilus DSM 3776 uid48643
Pleurocapsa PCC 7327 uid183006
Polaribacter MED152 uid54207
Polaromonas JS666 uid58207
Polaromonas naphthalenivorans CJ2 uid58273
Polymorphum gilvum SL003B 26A1 uid65447
Polynucleobacter necessarius STIR1 uid58967
Porphyromonas gingivalis ATCC 33277 uid58879
Porphyromonas gingivalis TDC60 uid67407
Porphyromonas gingivalis W83 uid57641
Prevotella dentalis DSM 3688 uid184818
Prevotella denticola F0289 uid65091
Prevotella intermedia 17 uid163151
Prevotella melaninogenica ATCC 25845 uid51377
Prevotella ruminicola 23 uid47507
Prochlorococcus marinus AS9601 uid58307
Prochlorococcus marinus CCMP1375 uid57995
Prochlorococcus marinus MIT 9211 uid58309
Prochlorococcus marinus MIT 9215 uid58819
Prochlorococcus marinus MIT 9301 uid58437
Prochlorococcus marinus MIT 9303 uid58305
Prochlorococcus marinus MIT 9312 uid58357
Prochlorococcus marinus MIT 9313 uid57773
Prochlorococcus marinus MIT 9515 uid58313
Prochlorococcus marinus NATL1A uid58423
Prochlorococcus marinus NATL2A uid58359

Selected prokaryotic genomes
Propionibacterium acnes 266 uid162059
Propionibacterium acnes 6609 uid162137
Propionibacterium acnes ATCC 11828 uid162177
Propionibacterium acnes C1 uid176501
Propionibacterium acnes HL096PA1 uid198524
Propionibacterium acnes KPA171202 uid58101
Propionibacterium acnes SK137 uid48071
Propionibacterium avidum 44067 uid197361
Propionibacterium propionicum F0230a uid170533
Prosthecochloris aestuarii DSM 271 uid58151
Proteus mirabilis HI4320 uid61599
Providencia stuartii MRSN 2154 uid162193
Pseudanabaena PCC 7367 uid183004
Pseudoalteromonas atlantica T6c uid58283
Pseudoalteromonas haloplanktis TAC125 uid58431
Pseudoalteromonas SM9913 uid61247
Pseudogulbenkiania NH8B uid73423
Pseudomonas aeruginosa B136 33 uid196598
Pseudomonas aeruginosa DK2 uid168996
Pseudomonas aeruginosa LESB58 uid59275
Pseudomonas aeruginosa M18 uid162089
Pseudomonas aeruginosa NCGM2 S1 uid162173
Pseudomonas aeruginosa PA7 uid58627
Pseudomonas aeruginosa PAO1 uid57945
Pseudomonas aeruginosa RP73 uid209328
Pseudomonas aeruginosa UCBPP PA14 uid57977
Pseudomonas brassicacearum NFM421 uid66303

Selected prokaryotic genomes
Pseudomonas denitrificans ATCC 13867 uid195459
Pseudomonas entomophila L48 uid58639
Pseudomonas fluorescens A506 uid165185
Pseudomonas fluorescens CHA0 uid203393
Pseudomonas fluorescens F113 uid87037
Pseudomonas fluorescens Pf0 1 uid57591
Pseudomonas fluorescens Pf 5 uid57937
Pseudomonas fluorescens SBW25 uid158693
Pseudomonas fulva 12 X uid67351
Pseudomonas mendocina NK 01 uid66299
Pseudomonas mendocina ymp uid58723
Pseudomonas ND6 uid167583
Pseudomonas poae RE 1 1 14 uid188480
Pseudomonas putida BIRD 1 uid162055
Pseudomonas putida DOT T1E uid171260
Pseudomonas putida F1 uid58355
Pseudomonas putida GB 1 uid58735
Pseudomonas putida H8234 uid208673
Pseudomonas putida HB3267 uid184078
Pseudomonas putida KT2440 uid57843
Pseudomonas putida NBRC 14164 uid208670
Pseudomonas putida S16 uid68747
Pseudomonas putida UW4 uid182733
Pseudomonas putida W619 uid58651
Pseudomonas resinovorans NBRC 106553 uid208671
Pseudomonas stutzeri A1501 uid58641
Pseudomonas stutzeri CCUG 29243 uid168379

Selected prokaryotic genomes
Pseudomonas stutzeri DSM 10701 uid170940
Pseudomonas stutzeri DSM 4166 uid162113
Pseudomonas stutzeri RCH2 uid184342
Pseudomonas syringae B728a uid57931
Pseudomonas syringae tomato DC3000 uid57967
Pseudonocardia dioxanivorans CB1190 uid65087
Pseudovibrio FO BEG1 uid82373
Pseudoxanthomonas spadix BD a59 uid75113
Pseudoxanthomonas suwonensis 11 1 uid62105
Psychrobacter arcticus 273 4 uid58021
Psychrobacter cryohalolentis K5 uid58373
Psychrobacter G uid210641
Psychrobacter PRwf 1 uid58459
Psychroflexus torquis ATCC 700755 uid54205
Psychromonas CNPT3 uid54249
Psychromonas ingrahamii 37 uid58521
Pusillimonas T7 7 uid66391
Pyrobaculum 1860 uid82379
Pyrobaculum aerophilum IM2 uid57727
Pyrobaculum arsenaticum DSM 13514 uid58409
Pyrobaculum calidifontis JCM 11548 uid58787
Pyrobaculum islandicum DSM 4184 uid58635
Pyrobaculum neutrophilum V24Sta uid58421
Pyrobaculum oguniense TE7 uid84411
Pyrococcus abyssi GE5 uid62903
Pyrococcus furiosus COM1 uid169620
Pyrococcus furiosus DSM 3638 uid57873

Selected prokaryotic genomes
Pyrococcus horikoshii OT3 uid57753
Pyrococcus NA2 uid66551
Pyrococcus ST04 uid167261
Pyrococcus yayanosii CH1 uid68281
Pyrolobus fumarii 1A uid73415
Rahnella aquatilis HX2 uid158049
Rahnella Y9602 uid62715
Ralstonia eutropha H16 uid62925
Ralstonia eutropha JMP134 uid58047
Ralstonia pickettii 12D uid58859
Ralstonia pickettii 12J uid58737
Ralstonia solanacearum CFBP2957 uid50545
Ralstonia solanacearum FQY 4 f uid194089
Ralstonia solanacearum GMI1000 uid57593
Ralstonia solanacearum Po82 uid162133
Ralstonia solanacearum PSI07 uid50539
Ralstonia solanacearum uid178852
Ramlibacter tataouinensis TTB310 uid68279
Raoultella ornithinolytica B6 uid198431
Renibacterium salmoninarum ATCC 33209 uid58899
Rhizobium etli bv mimosae Mim1 uid213896
Rhizobium etli CFN 42 uid58377
Rhizobium etli CIAT 652 uid59115
Rhizobium NGR234 uid59081
Rhizobium tropici CIAT 899 uid185179
Rhodanobacter 2APBS1 uid74431
Rhodobacter capsulatus SB 1003 uid47509

Selected prokaryotic genomes
Rhodobacter sphaeroides 2 4 1 uid57653
Rhodobacter sphaeroides ATCC 17025 uid58451
Rhodobacter sphaeroides ATCC 17029 uid58449
Rhodobacter sphaeroides KD131 uid59277
Rhodococcus equi 103S uid60171
Rhodococcus erythropolis PR4 uid59019
Rhodococcus jostii RHA1 uid58325
Rhodococcus opacus B4 uid13791
Rhodoferax ferrireducens T118 uid58353
Rhodomicrobium vannielii ATCC 17100 uid43247
Rhodopirellula baltica SH 1 uid61589
Rhodopseudomonas palustris BisA53 uid58445
Rhodopseudomonas palustris BisB18 uid58443
Rhodopseudomonas palustris BisB5 uid58441
Rhodopseudomonas palustris CGA009 uid62901
Rhodopseudomonas palustris DX 1 uid43327
Rhodopseudomonas palustris HaA2 uid58439
Rhodopseudomonas palustris TIE 1 uid58995
Rhodospirillum centenum SW uid58805
Rhodospirillum photometricum uid159003
Rhodospirillum rubrum ATCC 11170 uid57655
Rhodospirillum rubrum F11 uid162149
Rhodothermus marinus DSM 4252 uid41729
Rhodothermus marinus SG0 5JP17 172 uid72767
Rickettsia africae ESF 5 uid58799
Rickettsia akari Hartford uid58161
Rickettsia australis Cutlack uid158039

Selected prokaryotic genomes
Rickettsia bellii OSU 85 389 uid58681
Rickettsia bellii RML369 C uid58405
Rickettsia canadensis CA410 uid88063
Rickettsia canadensis McKiel uid58159
Rickettsia conorii Malish 7 uid57633
Rickettsia felis URRWXCal2 uid58331
Rickettsia heilongjiangensis 054 uid70839
Rickettsia japonica YH uid73963
Rickettsia massiliae AZT80 uid86751
Rickettsia massiliae MTU5 uid58801
Rickettsia montanensis OSU 85 930 uid158043
Rickettsia parkeri Portsmouth uid158045
Rickettsia peacockii Rustic uid59301
Rickettsia philipii 364D uid89383
Rickettsia prowazekii Breinl uid196851
Rickettsia prowazekii BuV67 CWPP uid158063
Rickettsia prowazekii Chernikova uid158053
Rickettsia prowazekii Dachau uid158057
Rickettsia prowazekii GvV257 uid158051
Rickettsia prowazekii Katsinyian uid158055
Rickettsia prowazekii Madrid E uid61565
Rickettsia prowazekii NMRC Madrid E uid196850
Rickettsia prowazekii Rp22 uid161945
Rickettsia prowazekii RpGvF24 uid158065
Rickettsia rickettsii Arizona uid86655
Rickettsia rickettsii Brazil uid88069
Rickettsia rickettsii Colombia uid86653

**Selected prokaryotic genomes**
Rickettsia rickettsii Hauke uid86659
Rickettsia rickettsii Hino uid86657
Rickettsia rickettsii Hlp 2 uid88067
Rickettsia rickettsii Iowa uid58961
Rickettsia rickettsii Sheila Smith uid58027
Rickettsia slovaca 13 B uid82369
Rickettsia slovaca D CWPP uid158159
Rickettsia typhi B9991CWPP uid158357
Rickettsia typhi TH1527 uid158161
Rickettsia typhi Wilmington uid58063
Riemerella anatipestifer RA CH 1 uid175469
Riemerella anatipestifer RA CH 2 uid186548
Riemerella anatipestifer RA GD uid162013
Rivularia PCC 7116 uid182929
Robiginitalea biformata HTCC2501 uid58285
Roseburia hominis A2 183 uid73419
Roseburia intestinalis uid197164
Roseburia intestinalis XB6B4 uid197179
Roseiflexus castenholzii DSM 13941 uid58287
Roseiflexus RS 1 uid58523
Roseobacter denitrificans OCh 114 uid58597
Roseobacter litoralis Och 149 uid54719
Rothia dentocariosa ATCC 17931 uid49331
Rothia mucilaginosa uid43093
Rubrivivax gelatinosus IL144 uid158163
Rubrobacter xylanophilus DSM 9941 uid58057
Ruegeria pomeroyi DSS 3 uid57863

Selected prokaryotic genomes
Ruegeria TM1040 uid58193
Ruminococcus albus 7 uid51721
Ruminococcus bromii uid197158
Ruminococcus champanellensis 18P13 uid197169
Ruminococcus obeum uid197165
Ruminococcus torques uid197166
Ruminococcus uid197156
Runella slithyformis DSM 19594 uid68317
Saccharomonospora viridis DSM 43017 uid59055
Saccharophagus degradans 2 40 uid57921
Saccharopolyspora erythraea NRRL 2338 uid62947
Saccharothrix espanaensis DSM 44229 uid184826
Salinarchaeum laminariae Harcht Bsk1 uid207001
Salinibacter ruber DSM 13855 uid58513
Salinibacter ruber M8 uid47323
Salinispora arenicola CNS 205 uid58659
Salinispora tropica CNB 440 uid58565
Salmonella bongori NCTC 12419 uid70155
Salmonella bongori Sbon 167 uid213088
Salmonella enterica serovar Typhi CT18 uid57793
Salmonella enterica serovar Typhi Ty2 uid57973
Sanguibacter keddieii DSM 10542 uid40845
Saprospira grandis Lewin uid89375
Sebaldella termitidis ATCC 33386 uid41865
Segniliparus rotundus DSM 44985 uid49049
Selenomonas sputigena ATCC 35185 uid55329
Serratia AS12 uid67315

Selected prokaryotic genomes
Serratia AS13 uid162065
Serratia liquefaciens ATCC 27592 uid212306
Serratia marcescens FGI94 uid185180
Serratia marcescens WW4 uid188478
Serratia odorifera 4Rx13 uid42253
Serratia plymuthica AS9 uid67313
Serratia plymuthica S13 uid210642
Serratia proteamaculans 568 uid58725
Serratia symbiotica Cinara cedri uid82363
Shewanella amazonensis SB2B uid58257
Shewanella ANA 3 uid58347
Shewanella baltica BA175 uid52601
Shewanella baltica OS117 uid162025
Shewanella baltica OS155 uid58259
Shewanella baltica OS185 uid58743
Shewanella baltica OS195 uid58261
Shewanella baltica OS223 uid58775
Shewanella baltica OS678 uid50553
Shewanella denitrificans OS217 uid58263
Shewanella frigidimarina NCIMB 400 uid58265
Shewanella halifaxensis HAW EB4 uid59007
Shewanella loihica PV 4 uid58349
Shewanella MR 4 uid58345
Shewanella MR 7 uid58343
Shewanella oneidensis MR 1 uid57949
Shewanella pealeana ATCC 700345 uid58705
Shewanella piezotolerans WP3 uid58745

Selected prokaryotic genomes
Shewanella putrefaciens 200 uid161927
Shewanella putrefaciens CN 32 uid58267
Shewanella sediminis HAW EB3 uid58835
Shewanella violacea DSS12 uid47085
Shewanella W3 18 1 uid58341
Shewanella woodyi ATCC 51908 uid58721
Shigella boydii CDC 3083 94 uid58415
Shigella boydii Sb227 uid58215
Shigella dysenteriae Sd197 uid58213
Shigella flexneri 2002017 uid159233
Shigella flexneri 2a 2457T uid57991
Shigella flexneri 2a 301 uid62907
Shigella flexneri 5 8401 uid58583
Shigella sonnei 53G uid84383
Shigella sonnei Ss046 uid58217
Sideroxydans lithotrophicus ES 1 uid46801
Simiduia agarivorans SA1 uid177713
Simkania negevensis Z uid68451
Singulisphaera acidiphila DSM 18658 uid81777
Sinorhizobium fredii HH103 uid86865
Sinorhizobium fredii USDA 257 uid168059
Sinorhizobium medicae WSM419 uid58549
Sinorhizobium meliloti 1021 uid57603
Sinorhizobium meliloti 2011 uid193772
Sinorhizobium meliloti AK83 uid52607
Sinorhizobium meliloti BL225C uid52605
Sinorhizobium meliloti GR4 uid184823

Selected prokaryotic genomes
Sinorhizobium meliloti Rm41 uid176372
Sinorhizobium meliloti SM11 uid159685
Slackia heliotrinireducens DSM 20476 uid59051
Sodalis glossinidius morsitans uid58553
Solibacillus silvestris StLB046 uid168516
Solitalea canadensis DSM 3403 uid81783
Sorangium cellulosum So0157 2 uid210741
Sorangium cellulosum So ce 56 uid61629
Sphaerobacter thermophilus DSM 20745 uid41997
Sphaerochaeta pleomorpha Grapes uid82365
Sphingobacterium 21 uid64755
Sphingobium chlorophenolicum L 1 uid52597
Sphingobium japonicum UT26S uid47077
Sphingobium SYK 6 uid73353
Sphingomonas MM 1 uid193771
Sphingomonas wittichii RW1 uid58691
Sphingopyxis alaskensis RB2256 uid58351
Spirochaeta africana DSM 8902 uid81779
Spirochaeta Buddy uid63633
Spirochaeta caldaria DSM 7334 uid68753
Spirochaeta coccoides DSM 17374 uid66331
Spirochaeta smaragdinae DSM 11293 uid51369
Spirochaeta thermophila DSM 6192 uid53037
Spirochaeta thermophila DSM 6578 uid162041
Spiroplasma chrysopicola DF 1 uid205053
Spiroplasma diminutum CUAS 1 uid212976
Spiroplasma syrphidicola EA 1 uid205054

Selected prokaryotic genomes
Spiroplasma taiwanense CT 1 uid212975
Spirosoma linguale DSM 74 uid43413
Stackebrandtia nassauensis DSM 44728 uid46663
Stanieria cyanosphaera PCC 7437 uid183115
Staphylococcus aureus 04 02981 uid161969
Staphylococcus aureus 08BA02176 uid175257
Staphylococcus aureus 11819 97 uid159981
Staphylococcus aureus 71193 uid162141
Staphylococcus aureus Bmb9393 uid210640
Staphylococcus aureus CC45 uid209174
Staphylococcus aureus COL uid57797
Staphylococcus aureus ECT R 2 uid159389
Staphylococcus aureus ED133 uid159689
Staphylococcus aureus ED98 uid41455
Staphylococcus aureus HO 5096 0412 uid162163
Staphylococcus aureus JH1 uid58457
Staphylococcus aureus JH9 uid58455
Staphylococcus aureus JKD6008 uid159855
Staphylococcus aureus JKD6159 uid159691
Staphylococcus aureus LGA251 uid159391
Staphylococcus aureus M013 uid88065
Staphylococcus aureus M1 uid197263
Staphylococcus aureus MRSA252 uid57839
Staphylococcus aureus MSHR1132 uid89393
Staphylococcus aureus MSSA476 uid57841
Staphylococcus aureus Mu3 uid58817
Staphylococcus aureus Mu50 uid57835

Selected prokaryotic genomes
Staphylococcus aureus MW2 uid57903
Staphylococcus aureus N315 uid57837
Staphylococcus aureus NCTC 8325 uid57795
Staphylococcus aureus Newman uid58839
Staphylococcus aureus RF122 uid57661
Staphylococcus aureus ST228 10388 uid193754
Staphylococcus aureus ST228 10497 uid193755
Staphylococcus aureus ST228 15532 uid193756
Staphylococcus aureus ST228 16035 uid193757
Staphylococcus aureus ST228 18412 uid193760
Staphylococcus aureus ST398 uid159247
Staphylococcus aureus T0131 uid159861
Staphylococcus aureus TCH60 uid159859
Staphylococcus aureus TW20 uid159241
Staphylococcus aureus uid193758
Staphylococcus aureus uid193759
Staphylococcus aureus uid193761
Staphylococcus aureus USA300 FPR3757 uid58555
Staphylococcus aureus USA300 TCH1516 uid58925
Staphylococcus aureus VC40 uid88071
Staphylococcus carnosus TM300 uid59401
Staphylococcus epidermidis ATCC 12228 uid57861
Staphylococcus epidermidis RP62A uid57663
Staphylococcus haemolyticus JCSC1435 uid62919
Staphylococcus lugdunensis HKU09 01 uid46233
Staphylococcus lugdunensis N920143 uid162143
Staphylococcus pseudintermedius ED99 uid162109

Selected prokaryotic genomes
Staphylococcus saprophyticus ATCC 15305 uid58411
Staphylococcus warneri SG1 uid187059
Staphylothermus hellenicus DSM 12710 uid45893
Staphylothermus marinus F1 uid58719
Starkeya novella DSM 506 uid48815
Stenotrophomonas maltophilia D457 uid162199
Stenotrophomonas maltophilia JV3 uid72473
Stenotrophomonas maltophilia K279a uid61647
Stenotrophomonas maltophilia R551 3 uid58657
Stigmatella aurantiaca DW4 3 1 uid158509
Streptobacillus moniliformis DSM 12112 uid41863
Streptococcus agalactiae 09mas018883 uid208674
Streptococcus agalactiae 2 22 uid202215
Streptococcus agalactiae 2603V R uid57943
Streptococcus agalactiae A909 uid57935
Streptococcus agalactiae GD201008 001 uid175780
Streptococcus agalactiae ILRI005 uid208676
Streptococcus agalactiae ILRI112 uid208675
Streptococcus agalactiae NEM316 uid61585
Streptococcus agalactiae SA20 06 uid178722
Streptococcus equi 4047 uid59259
Streptococcus equi zooepidemicus uid59261
Streptococcus gallolyticus ATCC 43143 uid162103
Streptococcus gallolyticus UCN34 uid46061
Streptococcus infantarius CJ18 uid87033
Streptococcus iniae SF1 uid206041
Streptococcus intermedius JTH08 uid168614

Selected prokaryotic genomes
Streptococcus lutetiensis 033 uid213397
Streptococcus macedonicus ACA DC 198 uid81631
Streptococcus mitis B6 uid46097
Streptococcus mutans GS 5 uid169223
Streptococcus mutans LJ23 uid162197
Streptococcus mutans NN2025 uid46353
Streptococcus mutans UA159 uid57947
Streptococcus oralis Uo5 uid65449
Streptococcus parasanguinis ATCC 15912 uid49313
Streptococcus parasanguinis FW213 uid163997
Streptococcus parauberis KCTC 11537 uid67355
Streptococcus pasteurianus ATCC 43144 uid68019
Streptococcus pneumoniae 670 6B uid52533
Streptococcus pneumoniae 70585 uid59125
Streptococcus pneumoniae AP200 uid52453
Streptococcus pneumoniae ATCC 700669 uid59287
Streptococcus pneumoniae CGSP14 uid59181
Streptococcus pneumoniae D39 uid58581
Streptococcus pneumoniae G54 uid59167
Streptococcus pneumoniae gamPNI0373 uid175861
Streptococcus pneumoniae Hungary19A 6 uid59117
Streptococcus pneumoniae INV104 uid162039
Streptococcus pneumoniae INV200 uid162035
Streptococcus pneumoniae JJA uid59121
Streptococcus pneumoniae OXC141 uid162037
Streptococcus pneumoniae P1031 uid59123
Streptococcus pneumoniae R6 uid57859

**Selected prokaryotic genomes**
Streptococcus pneumoniae SPN034156 uid197185
Streptococcus pneumoniae SPN034183 uid197186
Streptococcus pneumoniae SPN994038 uid197187
Streptococcus pneumoniae SPN994039 uid197188
Streptococcus pneumoniae SPNA45 uid174986
Streptococcus pneumoniae ST556 uid162191
Streptococcus pneumoniae Taiwan19F 14 uid59119
Streptococcus pneumoniae TCH8431 19A uid49735
Streptococcus pneumoniae TIGR4 uid57857
Streptococcus pseudopneumoniae IS7493 uid71153
Streptococcus pyogenes A20 uid178106
Streptococcus pyogenes Alab49 uid162171
Streptococcus pyogenes HSC5 uid212978
Streptococcus pyogenes M1 476 uid193766
Streptococcus pyogenes M1 GAS uid57845
Streptococcus pyogenes Manfredo uid57847
Streptococcus pyogenes MGAS10270 uid58571
Streptococcus pyogenes MGAS10394 uid58105
Streptococcus pyogenes MGAS10750 uid58575
Streptococcus pyogenes MGAS15252 uid158037
Streptococcus pyogenes MGAS1882 uid158061
Streptococcus pyogenes MGAS2096 uid58573
Streptococcus pyogenes MGAS315 uid57911
Streptococcus pyogenes MGAS5005 uid58337
Streptococcus pyogenes MGAS6180 uid58335
Streptococcus pyogenes MGAS8232 uid57871
Streptococcus pyogenes MGAS9429 uid58569

Selected prokaryotic genomes
Streptococcus pyogenes NZ131 uid59035
Streptococcus pyogenes SSI 1 uid57895
Streptococcus salivarius 57 I uid162151
Streptococcus salivarius CCHSS3 uid70481
Streptococcus salivarius JIM8777 uid162145
Streptococcus sanguinis SK36 uid58381
Streptococcus suis 05ZYH33 uid58663
Streptococcus suis 98HAH33 uid58665
Streptococcus suis A7 uid162111
Streptococcus suis BM407 uid59321
Streptococcus suis D12 uid162127
Streptococcus suis D9 uid162125
Streptococcus suis GZ1 uid161937
Streptococcus suis JS14 uid162095
Streptococcus suis P1 7 uid32235
Streptococcus suis S735 uid174333
Streptococcus suis SC070731 uid193769
Streptococcus suis SC84 uid59323
Streptococcus suis SS12 uid162123
Streptococcus suis ST1 uid167482
Streptococcus suis ST3 uid66327
Streptococcus suis TL13 uid203123
Streptococcus thermophilus CNRZ1066 uid58221
Streptococcus thermophilus JIM 8232 uid162157
Streptococcus thermophilus LMD 9 uid58327
Streptococcus thermophilus LMG 18311 uid58219
Streptococcus thermophilus MN ZLW 002 uid166827

**Selected prokaryotic genomes**
Streptococcus thermophilus ND03 uid162015
Streptococcus uberis 0140J uid57959
Streptomyces albus J1074 uid196849
Streptomyces avermitilis MA 4680 uid57739
Streptomyces bingchenggensis BCW 1 uid82931
Streptomyces cattleya NRRL 8057 uid77117
Streptomyces coelicolor A3 2 uid57801
Streptomyces davawensis JCM 4913 uid193657
Streptomyces flavogriseus ATCC 33331 uid40839
Streptomyces fulvissimus DSM 40593 uid201038
Streptomyces griseus NBRC 13350 uid58983
Streptomyces PAMC26508 uid197217
Streptomyces scabiei 87 22 uid46531
Streptomyces SirexAA E uid72627
Streptomyces venezuelae ATCC 10712 uid177080
Streptomyces violaceusniger Tu 4113 uid52609
Streptosporangium roseum DSM 43021 uid42521
Sulfobacillus acidophilus DSM 10332 uid88061
Sulfobacillus acidophilus TPY uid68841
Sulfolobus acidocaldarius DSM 639 uid58379
Sulfolobus acidocaldarius N8 uid189027
Sulfolobus acidocaldarius Ron12 I uid189028
Sulfolobus islandicus HVE10 4 uid162067
Sulfolobus islandicus LAL14 1 uid197216
Sulfolobus islandicus L D 8 5 uid43679
Sulfolobus islandicus L S 2 15 uid58871
Sulfolobus islandicus M 14 25 uid58849

Selected prokaryotic genomes
Sulfolobus islandicus M 16 27 uid58851
Sulfolobus islandicus M 16 4 uid58841
Sulfolobus islandicus REY15A uid162071
Sulfolobus islandicus Y G 57 14 uid58923
Sulfolobus islandicus Y N 15 51 uid58825
Sulfolobus solfataricus 98 2 uid167998
Sulfolobus solfataricus P2 uid57721
Sulfolobus tokodaii 7 uid57807
Sulfuricurvum kujiense DSM 16994 uid60789
Sulfurihydrogenibium azorense Az Fu1 uid58121
Sulfurihydrogenibium YO3AOP1 uid58855
Sulfurimonas autotrophica DSM 16294 uid53043
Sulfurimonas denitrificans DSM 1251 uid58185
Sulfurospirillum barnesii SES 3 uid168117
Sulfurospirillum deleyianum DSM 6946 uid41861
Sulfurovum NBC37 1 uid58863
Symbiobacterium thermophilum IAM 14863 uid58165
Synechococcus CC9311 uid58123
Synechococcus CC9605 uid58319
Synechococcus CC9902 uid58323
Synechococcus elongatus PCC 6301 uid58235
Synechococcus elongatus PCC 7942 uid58045
Synechococcus JA 2 3B a 2 13 uid58537
Synechococcus JA 3 3Ab uid58535
Synechococcus PCC 6312 uid182934
Synechococcus PCC 7002 uid59137
Synechococcus PCC 7502 uid183008

Selected prokaryotic genomes
Synechococcus RCC307 uid61609
Synechococcus WH 7803 uid61607
Synechococcus WH 8102 uid61581
Synechocystis PCC 6803 substr GT I uid157913
Synechocystis PCC 6803 substr GT I uid158059
Synechocystis PCC 6803 substr PCC N uid159835
Synechocystis PCC 6803 uid159873
Synechocystis PCC 6803 uid189748
Synechocystis PCC 6803 uid57659
Synergistetes bacterium SGP1 uid197182
Syntrophobacter fumaroxidans MPOB uid58177
Syntrophobotulus glycolicus DSM 8271 uid63343
Syntrophomonas wolfei Goettingen uid58179
Syntrophothermus lipocalidus DSM 12680 uid49527
Syntrophus aciditrophicus SB uid58539
Tannerella forsythia ATCC 43037 uid83157
Taylorella asinigenitalis 14 45 uid197194
Taylorella asinigenitalis MCE3 uid73771
Taylorella equigenitalis 14 56 uid197193
Taylorella equigenitalis ATCC 35865 uid170255
Taylorella equigenitalis MCE9 uid62103
Tepidanaerobacter acetatoxydans Re1 uid184827
Tepidanaerobacter Re1 uid66873
Teredinibacter turnerae T7901 uid59267
Terriglobus roseus DSM 18391 uid168183
Terriglobus saanensis SP1PR4 uid53251
Tetragenococcus halophilus uid74441

Selected prokaryotic genomes
Thalassobaculum L2 uid182483
Thalassolituus oleivorans MIL 1 uid195604
Thauera MZ1T uid58987
Thermacetogenium phaeum DSM 12270 uid177811
Thermaerobacter marianensis DSM 12885 uid61727
Thermincola potens JR uid48823
Thermoanaerobacter italicus Ab9 uid46241
Thermoanaerobacter mathranii A3 uid49481
Thermoanaerobacter tengcongensis MB4 uid57813
Thermoanaerobacter wiegelii Rt8 B1 uid52581
Thermoanaerobacter X513 uid53065
Thermoanaerobacter X514 uid58589
Thermobacillus composti KWC4 uid74021
Thermobaculum terrenum ATCC BAA 798 uid42011
Thermobifida fusca YX uid57703
Thermobispora bispora DSM 43833 uid48999
Thermococcus 4557 uid70841
Thermococcus AM4 uid54735
Thermococcus barophilus MP uid54733
Thermococcus CL1 uid168259
Thermococcus gammatolerans EJ3 uid59389
Thermococcus kodakarensis KOD1 uid58225
Thermococcus onnurineus NA1 uid59043
Thermococcus sibiricus MM 739 uid59399
Thermocrinis albus DSM 14484 uid46231
Thermodesulfatator indicus DSM 15286 uid68285
Thermodesulfobacterium OPB45 uid68283

Selected prokaryotic genomes
Thermodesulfobium narugense DSM 14796 uid66601
Thermofilum pendens Hrk 5 uid58563
Thermogladius 1633 uid167488
Thermomicrobium roseum DSM 5159 uid59341
Thermomonospora curvata DSM 43183 uid41885
Thermoplasma acidophilum DSM 1728 uid61573
Thermoplasmatales archaeon BRNA1 uid195930
Thermoplasma volcanium GSS1 uid57751
Thermoproteus tenax Kra 1 uid74443
Thermoproteus uzoniensis 768 20 uid65089
Thermosediminibacter oceani DSM 16646 uid51421
Thermosipho africanus TCF52B uid59095
Thermosipho melanesiensis BI429 uid58683
Thermosphaera aggregans DSM 11486 uid48993
Thermosynechococcus elongatus BP 1 uid57907
Thermotoga lettingae TMO uid58419
Thermotoga maritima MSB8 uid202924
Thermotoga maritima MSB8 uid57723
Thermotoga naphthophila RKU 10 uid42777
Thermotoga neapolitana DSM 4359 uid59065
Thermotoga petrophila RKU 1 uid58655
Thermotoga RQ2 uid58935
Thermotoga thermarum DSM 5069 uid68449
Thermovibrio ammonificans HB 1 uid62095
Thermovirga lienii DSM 17291 uid77129
Thermus CCB US3 UF1 uid81197
Thermus oshimai JL 2 uid178948

**Selected prokaryotic genomes**
Thermus scotoductus SA 01 uid62273
Thermus thermophilus HB27 uid58033
Thermus thermophilus HB8 uid58223
Thermus thermophilus JL 18 uid162129
Thermus thermophilus SG0 5JP17 16 uid159537
Thioalkalimicrobium cyclicum ALM1 uid67391
Thioalkalivibrio K90mix uid46181
Thiobacillus denitrificans ATCC 25259 uid58189
Thiocystis violascens DSM 198 uid74025
Thioflavicoccus mobilis 8321 uid184343
Thiomicrospira crunogena XCL 2 uid58183
Thiomonas 3As uid178369
Thiomonas intermedia K12 uid48825
Tistrella mobilis KA081020 065 uid167486
Tolumonas auensis DSM 9187 uid59395
Treponema azotonutricium ZAS 9 uid67365
Treponema brennaborense DSM 12168 uid66607
Treponema denticola ATCC 35405 uid57583
Treponema pallidum Chicago uid159543
Treponema pallidum DAL 1 uid87065
Treponema pallidum Fribourg Blanc uid201428
Treponema pallidum Mexico A uid176920
Treponema pallidum Nichols uid208669
Treponema pallidum Nichols uid57585
Treponema pallidum pertenue CDC2 uid87051
Treponema pallidum pertenue Gauthier uid87067
Treponema pallidum pertenue SamoaD uid87069

Selected prokaryotic genomes
Treponema pallidum SS14 uid58977
Treponema paraluiscuniculi Cuniculi A uid68447
Treponema primitia ZAS 2 uid67367
Treponema succinifaciens DSM 2489 uid65781
Trichodesmium erythraeum IMS101 uid57925
Tropheryma whipplei TW08 27 uid57961
Tropheryma whipplei Twist uid57705
Truepera radiovictrix DSM 17093 uid49533
Tsukamurella paurometabola DSM 20162 uid48829
Turneriella parva DSM 21527 uid168321
uncultured Sulfuricurvum RIFRC 1 uid193658
Ureaplasma parvum serovar 3 ATCC 27815 uid58887
Variovorax paradoxus EPS uid62107
Variovorax paradoxus S110 uid59437
Veillonella parvula DSM 2008 uid41927
Verminephrobacter eiseniae EF01 2 uid58675
Verrucosispora maris AB 18 032 uid66297
Vibrio anguillarum 775 uid68057
Vibrio cholerae IEC224 uid89389
Vibrio cholerae LMA3984 4 uid159541
Vibrio cholerae M66 2 uid59355
Vibrio cholerae MJ 1236 uid59387
Vibrio cholerae O1 2010EL 1786 uid78933
Vibrio cholerae O395 uid159869
Vibrio cholerae O395 uid58425
Vibrio EJY3 uid83161
Vibrio Ex25 uid41601

Selected prokaryotic genomes
Vibrio fischeri ES114 uid58163
Vibrio fischeri MJ11 uid58907
Vibrio furnissii NCTC 11218 uid82347
Vibrio harveyi ATCC BAA 1116 uid58957
Vibrio parahaemolyticus BB22OP uid184822
Vibrio parahaemolyticus RIMD 2210633 uid57969
Vibrio splendidus LGP32 uid59353
Vibrio vulnificus CMCP6 uid62909
Vibrio vulnificus MO6 24 O uid62243
Vibrio vulnificus YJ016 uid58007
Vulcanisaeta distributa DSM 14429 uid52827
Vulcanisaeta moutnovskia 768 28 uid63631
Waddlia chondrophila WSU 86 1044 uid49531
Weeksella virosa DSM 16922 uid63627
Weissella koreensis KACC 15510 uid68837
Wolbachia wRi uid59371
Wolinella succinogenes DSM 1740 uid61591
Xanthobacter autotrophicus Py2 uid58453
Xanthomonas albilineans GPE PC73 uid43163
Xanthomonas axonopodis citri 306 uid57889
Xanthomonas axonopodis citrumelo F1 uid73179
Xanthomonas axonopodis Xac29 1 uid193774
Xanthomonas campestris 8004 uid57595
Xanthomonas campestris ATCC 33913 uid57887
Xanthomonas campestris raphani 756C uid159539
Xanthomonas campestris uid61643
Xanthomonas citri Aw12879 uid194444

Selected prokaryotic genomes
Xanthomonas oryzae KACC 10331 uid58155
Xanthomonas oryzae MAFF 311018 uid58547
Xanthomonas oryzae oryzicola BLS256 uid54411
Xanthomonas oryzae PXO99A uid59131
Xenorhabdus bovienii SS 2004 uid46345
Xenorhabdus nematophila ATCC 19061 uid49133
Xylanimonas cellulosilytica DSM 15894 uid41935
Xylella fastidiosa 9a5c uid57849
Xylella fastidiosa GB514 uid162023
Xylella fastidiosa M12 uid58763
Xylella fastidiosa M23 uid58809
Xylella fastidiosa Temecula1 uid57869
Yersinia enterocolitica 8081 uid57741
Yersinia pestis A1122 uid158119
Yersinia pestis Angola uid58485
Yersinia pestis Antiqua uid58607
Yersinia pestis biovar Microtus 91001 uid58037
Yersinia pestis CO92 uid57621
Yersinia pestis D106004 uid158071
Yersinia pestis D182038 uid158073
Yersinia pestis KIM 10 uid57875
Yersinia pestis Nepal516 uid58609
Yersinia pestis Pestoides F uid58619
Yersinia pestis Z176003 uid47317
Yersinia pseudotuberculosis IP 31758 uid58487
Yersinia pseudotuberculosis IP 32953 uid58157
Yersinia pseudotuberculosis PB1 uid59153

Selected prokaryotic genomes
Yersinia pseudotuberculosis YPIII uid59151
Zobellia galactanivorans uid70621
Zunongwangia profunda SM A87 uid48073
Zymomonas mobilis ATCC 10988 uid55403
Zymomonas mobilis ATCC 29191 uid170612
Zymomonas mobilis NCIMB 11163 uid41019
Zymomonas mobilis pomaceae ATCC 29192 uid68445
Zymomonas mobilis ZM4 uid58095

Table S3. Distribution of ABCD transporters in Viridiplantae.

Species	half	full	Category
Cyanidioschyzon merolae strain 10D	3	0	Red algae
Chlamydomonas reinhardtii	2	0	Green algae
Micromonas pusilla	4	0	Green algae
Micromonas sp.	3	0	Green algae
Ostreococcus lucimarinus	3	0	Green algae
Ostreococcus tauri	2	0	Green algae
Volvox carteri	3	0	Green algae
Arabidopsis lyrata	1	1	Land plants
Arabidopsis thaliana	1	1	Land plants
Brachypodium distachyon	1	1	Land plants
Cicer arietinum	1	1	Land plants
Cucumis sativus	2	1	Land plants
Fragaria vesca	1	1	Land plants
Glycine max	0	4	Land plants
Medicago truncatula	1	1	Land plants
Oryza sativa Japonica Group	1	1	Land plants
Physcomitrella patens subsp. patens	4	2	Land plants
Populus trichocarpa	1	1	Land plants
Ricinus communis	2	1	Land plants
Selaginella moellendorffii	12	2	Land plants
Setaria italica	0	2	Land plants
Solanum lycopersicum	1	1	Land plants
Sorghum bicolor	2	2	Land plants
Vitis vinifera	1	0	Land plants
Zea mays	3	0	Land plants

Table S4. The genomes used to construct the phylogenetic tree.

Species	Kingdom	Category
Caldivirga maquilingensis IC 167 uid58711	Archaea	Thermoprotei
Methanococcus aeolicus Nankai 3 uid58823	Archaea	Methanococci
Archaeoglobus fulgidus DSM 4304 uid57717	Archaea	Archaeoglobi
Halalkalicoccus jeotgali B3 uid50305	Archaea	Halobacteria
Methanothermus fervidus DSM 2088 uid60167	Archaea	Methanobacteria
Methanococcoides burtonii DSM 6242 uid58023	Archaea	Methanomicrobia
Methanopyrus kandleri AV19 uid57883	Archaea	Methanopyri
Pyrococcus abyssi GE5 uid62903	Archaea	Thermococci
Thermoplasma acidophilum DSM 1728 uid61573	Archaea	Thermoplasmata
Nanoarchaeum equitans Kin4 M uid58009	Archaea	Nanoarchaeum
Cenarchaeum symbiosum A uid61411	Archaea	Cenarchaeales
Nitrosopumilus maritimus SCM1 uid58903	Archaea	Nitrosopumilales
Rubrobacter xylanophilus DSM 9941 uid58057	Bacteria	Actinobacteria
Persephonella marina EX H1 uid58119	Bacteria	Aquificae
Chthonomonas calidirosea T49 uid208678	Bacteria	Chthonomonadetes
Pedobacter heparinus DSM 2366 uid59111	Bacteria	Bacteroidetes
Chlorobium chlorochromatii CaD3 uid58375	Bacteria	Chlorobi
Ignavibacterium album JCM 16511 uid162097	Bacteria	Ignavibacteria
Caldisericum exile AZM16c01 uid158173	Bacteria	Caldisericia
Waddlia chondrophila WSU 86 1044 uid49531	Bacteria	Chlamydiae
Akkermansia muciniphila ATCC BAA 835 uid58985	Bacteria	Verrucomicrobia
Anaerolinea thermophila UNI 1 uid62245	Bacteria	Anaerolineae
Herpetosiphon aurantiacus DSM 785 uid58599	Bacteria	Chloroflexi
Dehalococcoides BAV1 uid58477	Bacteria	Dehalococcoidia
Thermomicrobium roseum DSM 5159 uid59341	Bacteria	Thermomicrobia
Desulfurispirillum indicum S5 uid45897	Bacteria	Chrysiogenetes
Gloeobacter violaceus PCC 7421 uid58011	Bacteria	Gloeobacteria

Species	Kingdom	Category
Calothrix PCC 6303 uid183109	Bacteria	Nostocales
Arthrospira platensis NIES 39 uid197171	Bacteria	Oscillatoriophycideae
Chroococcidiopsis thermalis PCC 7203 uid183002	Bacteria	Pleurocapsales
Prochlorococcus marinus AS9601 uid58307	Bacteria	Prochlorales
Deferribacter desulfuricans SSM1 uid46653	Bacteria	Deferribacteres
Marinithermus hydrothermalis DSM 14884 uid65783	Bacteria	Deinococci
Dictyoglomus thermophilum H 6 12 uid59439	Bacteria	Dictyoglomia
Elusimicrobium minutum Pei191 uid58949	Bacteria	Elusimicrobia
Acidobacterium capsulatum ATCC 51196 uid59127	Bacteria	Acidobacteria
Fibrobacter succinogenes S85 uid161919	Bacteria	Fibrobacteres
Lactococcus garvieae ATCC 49156 uid73413	Bacteria	Bacilli
Thermodesulfobium narugense DSM 14796 uid66601	Bacteria	Clostridia
Erysipelothrix rhusiopathiae Fujisawa uid68021	Bacteria	Erysipelotrichi
Megamonas hypermegale uid197163	Bacteria	Negativicutes
Leptotrichia buccalis C 1013 b uid59211	Bacteria	Fusobacteriia
Gemmatimonas aurantiaca T 27 uid58813	Bacteria	Gemmatimonadetes
Leptospirillum ferriphilum ML 04 uid175904	Bacteria	Nitrospira
Phycisphaera mikurensis NBRC 102666 uid157331	Bacteria	Phycisphaerae
Isosphaera pallida ATCC 43644 uid62207	Bacteria	Planctomycetia
Novosphingobium PP1Y uid67383	Bacteria	Alphaproteobacteria
Aromatoleum aromaticum EbN1 uid58231	Bacteria	Betaproteobacteria
Nautilia profundicola AmH uid59345	Bacteria	delta/epsilonsubdivisions
Escherichia coli K 12 substr DH10B uid58979	Bacteria	Gammaproteobacteria
Borrelia afzelii HLJ01 uid177930	Bacteria	Spirochaetia
Aminobacterium colombiense DSM 12261 uid47083	Bacteria	Synergistia
Mycoplasma agalactiae PG2 uid61619	Bacteria	Mollicutes
Thermodesulfatator indicus DSM 15286 uid68285	Bacteria	Thermodesulfobacteria
Fervidobacterium nodosum Rt17 B1 uid58625	Bacteria	Thermotogae

Species	Kingdom	Category
Tetrahymena thermophila SB210	Eukaryote	Alveolata
Plasmodium falciparum 3D7	Eukaryote	Alveolata
Dictyostelium discoideum AX4	Eukaryote	Amoebozoa
Entamoeba histolytica HM-1:IMSS	Eukaryote	Amoebozoa
Leishmania major strain Friedlin	Eukaryote	Euglenozoa
Trypanosoma brucei	Eukaryote	Euglenozoa
Schizosaccharomyces pombe	Eukaryote	Fungi
Saccharomyces cerevisiae S288c	Eukaryote	Fungi
Homo sapiens	Eukaryote	Metazoa
Mus musculus	Eukaryote	Metazoa
Caenorhabditis elegans	Eukaryote	Metazoa
Drosophila melanogaster	Eukaryote	Metazoa
Danio rerio	Eukaryote	Metazoa
Giardia lamblia ATCC 50803	Eukaryote	Fornicata
Monosiga brevicollis MX1	Eukaryote	Choanoflagellida
Arabidopsis thaliana	Eukaryote	Viridiplantae
